# Supplementary material for: Chemical tools for epichaperome-mediated interactome dysfunctions of the central nervous system
Source: Nat Commun. 2021 Aug 3;12:4669. doi: 10.1038/s41467-021-24821-2 (PMC8333062; doi:10.1038/s41467-021-24821-2)
Supplement: Supplementary file 1 — Supplementary Information [file 41467_2021_24821_MOESM1_ESM.pdf]

Supplementary Information

Chemical tools for epichaperome-mediated interactome dysfunctions of the central nervous system

Bolaender et al.

Contains:

Supplementary Tables 1 through 12

Supplementary Figures 1 through 20

Supplementary Note 1: synthetic procedures

Supplementary Note 2: clinical study protocol, NCT03371420

Supplementary Table 1.

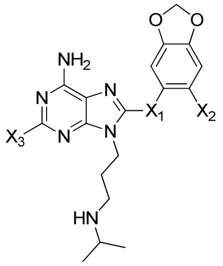

Compound name	Compound code	X_1	X_2	X_3	[a]mol_MW	[a]PSA	[a]$QPlogP_{o/w}$	FP relative affinity (nM)[b]	PAMPA-BBB (10^{-6} cms^{-1})[b]
PU-H71	**1**	S	I	H	512.367	90.689	2.914	11±4	7.3±0.5
PU-H64	**3**	S	Br	H	465.367	90.48	2.857	122±7	6.1±0.2
PU-WS5	**5**	S	CN	H	411.481	120.099	1.884	285±13	4.8±0.4
PU-DZ4-22-3	**6**	S	$COCH_3$	H	428.508	116.444	1.922	103±7	1.5±0.3
PU-HT65	**7**	S	Vinyl	H	412.509	94.346	3.083	213±7	6.1±0.1
PU-HT64	**8**	S	Allyl	H	426.535	96.162	3.181	122±4	6.7±0.7
PU-DZ2-388	**9**	S	Phenyl	H	462.568	93.549	3.851	270±10	9.9±0.5
PU-DZ3-6	**10**	S	4-Bromophenyl	H	541.464	93.83	4.42	732±13	11.5±0.4
PU-DZ2-390	**11**	S	4-*t*-Butylphenyl	H	518.676	95.832	4.73	2,600±83	12.5±0.6
PU-DZ2-392	**12**	S	4-Dimethyl-aminophenyl	H	505.637	97.73	4.154	1,388±44	9.2±0.3
PU-DZ8	**17**	CH_2	I	F	512.325	95.24	3.109	85±10	6.2±1.2
PU-DZ10	**18**	CH_2	Br	F	465.324	96.416	2.83	191±6	5.9±0.6
PU-WS3	**20**	CH_2	CN	F	411.438	120.735	1.922	219±19	1.5±0.2
PU-HT133	**21**	CH_2	Ethyl	F	414.482	94.656	3.228	813±56	5.9±0.6
PU-HT70	**22**	CH_2	Vinyl	F	412.466	89.093	2.683	146±9	5.6±0.2
PU-HT78	**23**	CH_2	Allyl	F	426.493	95.703	3.547	>5,000	4.6±0.6

[a] The molecular weight (mol_MW), polar surface area (PSA) and octanol/water partition coefficient ($QPlogP_{o/w}$) were calculated using QikProp.

[b] FP and PAMPA-BBB values, mean±SD, n = 3 replicates.

Supplementary Table 2.

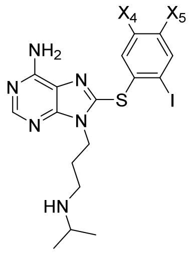

**Compound name**	**Compound code**	**X_4,X_5**	**[a]mol_MW**	**[a]PSA**	**[a]$QPlogP_{o/w}$**	**FP relative affinity (nM) [b]**	**PAMPA-BBB (10^{-6} cms^{-1}) [b]**
PU-RK11	**28**	OCH_2CH_2O	526.394	93.454	3.048	34.5±1.7	6.6±0.6
PU-DZ3-117	**35**	$OCH_2CH_2CH_2O$	540.421	92.078	3.78	232±10	10.4±0.3
PU-WS4	**40**	OCH_2CH_2	510.395	85.795	3.479	8.0±0.4	7.7±0.8
PU-WS10	**45**	CH_2CH_2O	510.395	84.977	3.624	132.9±5.8	7.2±0.1
PU-WS29	**53**	$CH_2CH_2CH_2$	508.422	76.28	3.788	4.5±0.5	15.1±0.7
PU-DZ4-52-N9	**58**	CHCHCHCH	518.417	76.611	4.341	97±3	10.8±0.2

[a] Calculated using QikProp.

[b] FP and PAMPA-BBB values, mean±SD, n = 3 replicates.

Supplementary Table 3.

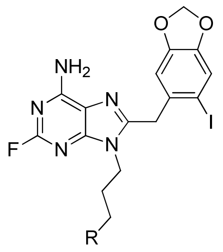

Compound name	Compound code	R	[a]mol_MW	[a]PSA	[a]$QPlogP_{o/w}$	FP relative affinity (nM) [b]	PAMPA-BBB (10^{-6} cms^{-1}) [b]
PU-DZ4-80	**59**	*tert*-butylamine	526.351	92.481	3.295	12.7±1.6	13.3±1.4
PU-BSI12	**60**	N-isopropylmethylamine	526.351	87.965	3.197	39±2	5.8±1.3
PU-BSI8	**61**	N-ethylisopropylamine	540.378	87.373	3.502	30±13	7.5±0.2
PU-BSI13	**62**	N-ethylmethylamine	512.325	88.934	3.044	45±19	7.5±0.1
PU-BSI14	**63**	N-(cyclopropylmethyl)propan-1-amine	566.416	85.344	3.909	43±4	8.4±2.1
PU-BSI11	**64**	N-methylpropargylamine	522.32	88.293	3.121	30±6	11.1±0.1
PU-BSI7	**65**	N-allylmethylamine	524.336	88.251	3.429	44±4	11.3±1.9
PU-BSI10	**66**	2-methylaziridine	510.309	90.809	4.262	105±5	5.8±1.5
PU-BSI15	**67**	Piperidine	538.362	86.657	2.966	112±6	6.9±0.2
PU-BSI16	**68**	Morpholine	540.335	97.849	2.629	202±7	6.3±1.1
PU-BSI6	**69**	2-(isoproylamino)ethanol	556.378	112.151	2.384	61±2	1.1±0.1
PU-BSI5	**70**	N-cyclohexylethanolamine	596.442	111.203	3.379	24±2	8.5±0.1
PU-BSI3	**71**	1-(2-hydroxyethyl)piperazine	583.403	117.76	1.664	200±5	4.7±0.5
PU-BSI4	**72**	Trimethylamine	ND	ND	ND	155±5	1.3±0.1

[a] Calculated using QikProp.

[b] FP and PAMPA-BBB values, mean±SD, n = 3 replicates.

Supplementary Table 4.

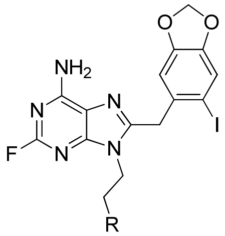

Compound name	Compound code	R	^{a}mol_MW	^{a}PSA	^{a}QPlogP$_{o/w}$	FP relative affinity (nM) [b]	PAMPA-BBB (10^{-6} cms^{-1}) [b]
PU-DZ12	**74**	Isopropylamine	498.298	93.959	2.85	219±5	5.2±2.6
PU-DZ4-81	**75**	*tert*-butylamine	512.325	94.802	3.311	198±2	13.5±1.5
PU-DZ13	**76**	Isobutylamine	512.325	95.719	3.208	7.2±1.2	12.3±1.5
PU-DZ14	**77**	Neopentylamine	526.351	94.284	3.531	7.8±1.1	13.1±0.3
PU-DZ16	**78**	Cyclopropylmethylamine	510.309	96.563	3.01	36±5	8.6±0.3
PU-DZ15	**79**	*N*-ethylmethylamine	498.298	86.73	2.555	166±5	11.2±1.7
PU-DZ17	**80**	*N*-isobutylmethylamine	526.351	87.778	3.33	92±16	10.1±1.0
PU-DZ18	**81**	N-methylpropargylamine	508.293	88.667	2.931	107±18	9.7±1.2

[a] Calculated using QikProp.
[b] FP and PAMPA-BBB values, mean±SD, n = 3 replicates.

Supplementary Table 5.

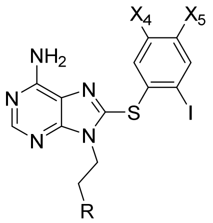

Compound name	Compound code	R	X4,X5	[a]mol_MW	[a]PSA	[a]QPlogP$_{o/w}$	[b]FP relative affinity (nM)	[b]PAMPA-BBB (10^{-6} cms^{-1})
PU-HZ150	**84**	Isobutylamine	OCH_2O	512.367	95.221	3.089	14±2	8.9±0.5
PU-HZ151	**85**	Neopentylamine	OCH_2O	526.394	92.27	3.165	5±1	15.4±2.6
PU-WS9	**86**	Isobutylamine	OCH_2CH_2	510.395	85.935	3.654	5.5±0.6	9.3±0.2
PU-WS21	**87**	Neopentylamine	OCH_2CH_2	524.422	84.435	3.98	10.8±0.6	13.5±0.7

[a] Calculated using QikProp.

[b] FP and PAMPA-BBB values, mean±SD, n = 3 replicates.

Supplementary Table 6.

Compd code	[a]logS	[a]AlogP	[b]QPlogP	[c]ClogD$_{7.4}$	[b]PSA	[b]mol_MW	[b]HBD	CpKa_Epik	pKa_ChemDraw	PAMPA_BBB	[d]CNS MPO Scores		
											A	B	C
**1**	-3.87	2.61	2.914	1.21	90.689	512.37	3	10.09	9.494	7.3±0.5	3.1	3.1	3.4
**3**	-4.02	2.49	2.857	1.01	90.48	465.37	3	10.09	9.495	6.1±0.2	3.4	3.4	3.7
**5**	-3.34	1.57	1.884	0.03	120.1	411.48	3	10.09	9.488	4.8±0.4	2.8	2.8	3.1
**6**	-3.57	1.59	1.922	-0.47	116.44	428.51	3	10.09	9.492	1.5±0.3	2.8	2.8	3
**7**	-3.81	2.32	3.083	1.11	94.346	412.51	3	10.09	9.495	6.1±0.1	3.6	3.6	3.9
**8**	-4.02	2.66	3.181	1.26	96.162	426.54	3	10.09	9.498	6.7±0.7	3.5	3.4	3.6
**9**	-4.39	3.18	3.851	1.9	93.549	462.57	3	10.09	9.494	9.9±0.5	3.2	2.9	3.1
**10**	-4.93	3.81	4.42	2.69	93.83	541.46	3	10.09	9.494	11.5±0.4	2.3	2	2.2
**11**	-5.02	4.58	4.73	3.53	95.832	518.68	3	10.09	9.493	12.5±0.6	1.4	1.3	1.6
**12**	-4.44	3.76	4.154	2.16	97.73	505.64	3	10.09	9.495	9.2±0.3	2.4	2.3	2.5
**17**	-4.11	2.93	3.109	0.5	95.24	512.33	3	10.09	9.486	6.2±1.2	3	2.9	3.2
**18**	-4.12	3.14	2.83	0.3	96.416	465.32	3	10.09	9.487	5.9±0.6	3.1	3.2	3.5
**20**	-3.52	2.27	1.922	-0.68	120.74	411.44	3	10.09	9.481	1.5±0.2	2.8	2.8	3.1
**21**	-3.97	2.99	3.228	0.37	94.656	414.48	3	10.09	9.49	5.9±0.6	3.6	3.5	3.8
**22**	-4.02	2.88	2.683	0.4	89.093	412.47	3	10.09	9.488	5.6±0.2	3.8	3.8	4
**23**	-4.18	3.08	3.547	0.55	95.703	426.49	3	10.09	9.489	4.6±0.6	3.5	3.2	3.5
**28**	-3.99	2.82	3.048	0.84	93.454	526.39	3	10.09	9.505	6.6±0.6	3.1	3	3.3
**35**	-4.15	3.08	3.78	0.89	92.078	540.42	3	10.09	9.505	10.4±0.3	3.1	2.7	3
**44**	-4.17	3.13	3.479	1.25	85.795	510.4	3	10.09	9.49	7.7±0.8	3.1	2.9	3.2
**45**	-4.17	3.11	3.624	1.25	84.977	510.4	3	10.09	9.495	7.2±0.1	3.1	2.9	3.1
**53**	-4.42	3.72	3.788	2.35	76.28	508.42	3	10.09	9.491	15.1±0.7	2.6	2.6	2.9
**58**	-4.52	4.25	4.341	2.52	76.611	518.42	3	10.09	9.49	10.8±0.2	2.3	2.2	2.5
**59**	-4.33	3.58	3.295	0.51	92.481	526.35	3	10.09	9.532	13.3±1.4	2.8	2.9	3.2
**60**	-4.09	3.67	3.197	1.1	87.965	526.35	2	9.21	9.435	5.8±1.3	3.6	3.8	3.7
**61**	-4.23	4.05	3.502	1.2	87.373	540.38	2	9.21	9.465	7.5±0.2	3.4	3.6	3.5
**62**	-3.91	3.34	3.044	0.95	88.934	512.33	2	9.21	9.37	7.5±0.1	3.7	3.9	3.8
**63**	-4.58	4.59	3.909	1.37	85.344	566.42	2	9.64	9.266	8.4±2.1	2.9	3.2	3.4
**64**	-4.30	3.12	3.121	1.98	88.293	522.32	2	6.38	8.015	11.1±0.1	4.4	4.4	4.4
**65**	-4.22	3.54	3.429	1.96	88.251	524.34	2	8.49	8.999	11.3±1.9	4	4	3.8
**66**	-3.72	3.32	4.262	3.3	90.809	510.31	2	8.78	11.187	5.8±1.5	3.3	2.8	2.2
**67**	-4.29	3.73	2.966	1.64	86.657	538.36	2	9.53	9.198	6.9±0.2	3.4	3.7	3.9
**68**	-3.84	2.77	2.629	2.46	97.849	540.34	2	7.1	7.164	6.3±1.1	4	4	4
**69**	-4.00	3.04	2.384	0.61	112.15	556.38	3	8.49	8.629	1.1±0.1	3.2	3.2	3.1
**70**	-4.40	3.80	3.379	1	111.2	596.44	3	9.76	8.563	8.5±0.1	2.2	2.4	3
**71**	-3.52	2.26	1.664	1.31	117.76	583.4	3	4	3.399	4.7±0.5	3.2	3.2	3.2
**72**	ND	ND	ND	ND	ND	ND	ND	ND	ND	1.3±0.1	ND	ND	ND
**74**	-3.97	2.61	2.85	0.75	93.959	498.3	3	9.47	8.758	5.2±2.6	3.3	3.3	3.7
**75**	-4.15	3.19	3.311	0.75	94.802	512.33	3	9.47	8.804	13.5±1.5	3.2	3.1	3.4
**76**	-4.13	3.11	3.208	1.04	95.719	512.33	3	9.72	8.708	12.3±1.5	3.1	3	3.5
**77**	-4.34	3.48	3.531	1.63	94.284	526.35	3	9.8	8.749	13.1±0.3	2.9	2.9	3.4
**78**	-4.09	2.87	3.01	0.63	96.563	510.31	3	9.72	8.547	8.6±0.3	3.1	3.1	3.7
**79**	-3.73	3.18	2.555	1.48	86.73	498.3	2	8.49	8.643	11.2±1.7	4.2	4.3	4.2
**80**	-4.09	3.59	3.33	1.86	87.778	526.35	2	8.79	8.662	10.1±1.0	3.8	3.9	4
**81**	-4.24	2.94	2.931	2.48	88.667	508.29	2	5.65	7.289	9.7±1.2	4.3	4.3	4.3
**84**	-3.87	2.59	3.089	1.78	95.221	512.37	3	9.72	8.669	8.9±0.5	3.1	3.1	3.6
**85**	-4.01	3.00	3.165	2.37	92.27	526.39	3	9.8	8.711	15.4±2.6	3	2.9	3.5
**86**	-4.17	3.08	3.654	1.83	85.935	510.4	3	9.72	8.665	9.3±0.2	3.3	3	3.5
**87**	-4.29	3.40	3.98	2.41	84.435	524.42	3	9.8	8.706	13.5±0.7	2.9	2.6	3.1

[a] logS and AlogP were calculated using ALOGPS 2.1 (VCCLAB)

[b] QPlogP, PSA, mol_MW and HBD were calculated using Qikprop.

[c] ClogD$_{7.4}$ was calculated using MarvinSketch 20.19.

[d] CNS MPO Scores based on:

A: AlogP, ClogD$_{7.4}$, PSA, mol_MW, HBD and Cp*K*a_Epik

B: QPlogP, ClogD$_{7.4}$, PSA, mol_MW, HBD and Cp*K*a_Epik

C: QPlogP, ClogD$_{7.4}$, PSA, mol_MW, HBD and pKa_ChemDraw

Calculated molecular properties and experimentally determined PAMPA-BBB values.

Supplementary Table 7.

Receptor	Percent Inhibition (Average; N= 2) 1.0E-5 M	Receptor	Percent Inhibition (Average; N= 2) 1.0E-5 M
**NEUROTRANSMITTER RELATED**		**NEUROTRANSMITTER RELATED**	
Serotonin Transporter	5.96%	Adenosine Transporter (h)	-3.54%
Serotonin, 5HT1A (h)	-16.30%	Adenosine, A1	14.00%
Serotonin, 5HT1D	5.13%	Adenosine, A2A (h)	0.79%
Serotonin, 5HT2A	8.50%	Adrenergic, Alpha 1A	12.17%
Serotonin, 5HT2C	12.77%	Adrenergic, Alpha 1B	27.43%
Serotonin, 5HT3	6.61%	Adrenergic, Alpha 2A (h)	-1.92%
Serotonin, 5HT4	36.08%	Adrenergic, Alpha 2B	10.67%
Serotonin, 5HT5A (h)	11.09%	Adrenergic, Alpha 2C (h)	15.07%
Serotonin, 5HT6 (h)	17.73%	Adrenergic, Beta 1 (h)	-0.96%
Serotonin, 5HT7 (h)	14.36%	Adrenergic, Beta 2 (h)	3.05%
Sigma 1		Dopamine Transporter	-12.87%
Sigma 2	-0.71%	Dopamine, D1 (h)	8.03%
**ION CHANNELS**		Dopamine, D2s (h)	20.57%
Calcium Channel, Type L (Dihydropyridine)	-11.28%	Dopamine, D3	-6.23%
Calcium Channel, Type N	-11.15%	Dopamine, D4.4 (h)	10.85%
GABA, Chloride, TBOB Site	-28.46%	GABA A, Agonist Site	33.91%
Potassium Channel, ATP-Sensitive	-1.01%	GABA A, BDZ, alpha 1 site	18.61%
Potassium Channel, Ca2+ Act., VI	18.66%	GABA-B	18.61%
Potassium Channel, I[Kr] (hERG) (h)	-17.96%	Glutamate, AMPA Site (Ionotropic)	8.62%
Sodium, Site 2	14.53%	Glutamate, Kainate Site (Ionotropic)	-7.63%
**SECOND MESSENGERS**		Glutamate, MK-801 Site (Ionotropic)	-0.50%
Nitric Oxide, NOS (Neuronal-Binding)	-1.73%	Glutamate, NMDA Agonist Site (Ionotropic)	23.69%
**PROSTAGLANDINS**		Glutamate, NMDA, Phencyclidine Site (Ionotropic)	6.26%
Leukotriene, LTB4 (BLT)	22.06%	Glutamate,NMDA,Glycine (Stry-insens Site) (Ionot	-0.97%
Leukotriene, LTD4 (CysLT1)	28.77%	Glycine, Strychnine-sensitive	-19.46%
Thromboxane A2 (h)	20.84%	Histamine, H1	5.94%
**BRAIN/GUT PEPTIDES**		Histamine, H2	40.98%
Angiotensin II, AT1 (h)	0.36%	Histamine, H3	22.25%
Bradykinin, BK2	10.14%	Muscarinic, M1 (hr)	26.84%
Endothelin, ET-A (h)	13.54%	Muscarinic, M2 (h)	45.44%
Neurokinin, NK1	20.40%	**Muscarinic, M3 (h)**	**50.76%**
Neuropeptide, NPY2 (h)	28.79%	Muscarinic, M4 (h)	27.60%
**ENZYMES**		**Muscarinic, M5 (h)**	**69.89%**
Esterase, Acetylcholine	6.63%	Nicotinic, Neuronal (a-BnTx insensitive)	-17.51%
Phosphodiesterase, PDE4A1A (h)	14.00%	Norepinephrine Transporter	5.73%
Phosphodiesterase, PDE5A1 (h)	-35.00%	Opioid, Delta 2 (h)	20.37%
**ENZYMES, KINASES**		**Opioid, Mu (h)**	**56.70%**
Kinase, Protein, PKA (h)	0.09%		
Kinase, Protein, PKCa (h)	11.20%		

Values are expressed as the percent inhibition of specific binding and represent the average of replicate tubes at each of the concentrations tested. Bolded values represent inhibition of 50% or greater.

PU-HZ151 shows little to no off-target activity *in vitro* on proteins in the General SEP II panel. PU-HZ151 (at 10 μM) was tested against 70 key proteins, including neurotransmitter receptors, ion channels, ion pumps, synthetic enzymes, and transporter proteins comprising the diversity panel screen (General SEP II). Test was performed by Caliper Life Sciences. Binding was expressed as a mean percent of the reference control (n = 2 measurements) collected at a single, high (10 μM) concentration of PU-HZ151. See also Methods.

Supplementary Table 8.

		N	CYPP450<br>ABT	CYP1A2<br>F	CYP3A4<br>K	CYP3A4<br>Td	CYP2A6<br>Tp	CYP2B6<br>O	CYP2C8<br>M	CYP2C9<br>S	CYP2D6<br>Q	CYP2C19<br>B	CYP inhibitor
PU-HZ151	15 min	52.7	90.5	65.9	76.5	79.8	57.2	62	59.7	70.5	62.9	59.2	% remaining
	30 min	34.7	83.9	47.3	63.5	66.9	36.6	42.7	42.1	54.1	43.9	39.3	
	60 min	17.2	79.6	26.3	50.6	50.9	18.4	25.6	22.4	36.5	26.6	20.2	

N: No inhibitor
ABT: 1-Aminobenzotriazole (a general P450 inhibitor)
F: Furafylline (CYP1A2 inhibitor)
K: Ketoconazole (CYP3A4 inhibitor)
Td: Troleandomycin (CYP3A4 inhibitor)
Tp: Tranylcypromine (CYP2A6 inhibitor)
O: Orphenadrine (CYP2B6 inhibitor)
M: Montelukast (CYP2C8 inhibitor)
S: Sulfaphenazole (CYP2C9 inhibitor)
Q: Quinidine (CYP2D6 inhibitor)
B: Benzylnirvanol (CYP2C19 inhibitor)

Cytochrome P450 Profiling of PU-HZ151 in Human Liver Microsomes. The cytochrome P450 enzymes responsible for the metabolism of PU-HZ151 were identified by measuring the inhibition of PU-HZ151 metabolism in human liver microsomes by specific inhibitors of various P450 isoforms (CYP1A2 inhibitor: furafylline; CYP2A6 inhibitor: tranylcypromine; CYP3A inhibitor: ketoconazole; CYP3A4 inhibitor: troleandomycin; CYP2B6 inhibitor: orphenadrine; CYP2C8 inhibitor: montelukast; CYP2C9 inhibitor: sulfaphenazole; CYP2C19 inhibitor: benzylnirvanol; CYP2D6 inhibitor: quinidine; non-specific P450 inhibitor: 1-aminobenzotriazole). In the absence of any inhibitor, 17.2% of PU-HZ151 remained after 60 min. The metabolism of PU-HZ151 was significantly inhibited (75%) in the presence of the non-specific inhibitor 1-aminobenzotriazole. The metabolism of PU-HZ151 was also significantly inhibited (40%) in the presence of ketoconazole (CYP3A inhibitor) or troleandomycin (CYP3A4 inhibitor), and to a lesser extent in the presence of the CYP2C9 inhibitor sulfaphenazole (23%). The metabolism of PU-HZ151 was only slightly inhibited in the presence of furafylline (CYP1A2 inhibitor; 11%), quinidine (CYP2D6 inhibitor; 11%), orphenadrine (CYP2B6 inhibitor; 10%), montelukast (CYP2C8 inhibitor; 6%), and benzylnirvanol (CYP2C19 inhibitor; 4%). There was essentially no effect on metabolism of PU-HZ151 in the presence of tranylcypromine (CYP2A6 inhibitor).

Supplementary Table 9.

Peak ID/Human	Proposed identity	R.T. (min)	Peak area#1	% Peak area of metabolite	R.T. (min)	Peak area#2	% Peak area of metabolite
M5	Desneopentyl + Desmethylene PU-HZ151	4.41	4.75E+05	2.5	4.40	5.05E+05	2.9
M1	Desneopentyl PU-HZ151	4.98	1.02E+07	53.0	4.98	1.03E+07	59.1
M2	Desmethylene PU-HZ151	5.37	2.50E+06	13.0	5.38	1.57E+06	9.0
M3a	PU-HZ151+ Oxygen	5.55	4.44E+06	23.1	5.57	3.59E+06	20.6
M3b	PU-HZ151+ Oxygen	5.67	1.11E+06	5.8	5.69	1.02E+06	5.8
PU-HZ151	PU-HZ151	5.94	9.32E+07	-	5.98	7.20E+07	-
M4	PU-HZ151 + 14 amu	6.86	5.03E+05	2.6	6.93	4.37E+05	2.5

Total peak area of metabolites: 1.92E+07 Total peak area of metabolites: 1.74E+07

Peak ID/Mouse	Proposed identity	R.T. (min)	Peak area#1	% Peak area of metabolite	R.T. (min)	Peak area#2	% Peak area of metabolite
M5	Desneopentyl + Desmethylene PU-HZ151	4.42	3.68E+06	11.6	4.40	4.81E+06	11.7
M1	Desneopentyl PU-HZ151	5.00	6.16E+06	19.5	4.98	8.66E+06	21.0
M2	Desmethylene PU-HZ151	5.40	2.18E+07	68.9	5.38	2.78E+07	67.4
M3a	PU-HZ151+ Oxygen	NP	0	0	NP	0	0
M3b	PU-HZ151+ Oxygen	NP	0	0	NP	0	0
PU-HZ151	PU-HZ151	6.01	5.74E+06	-	5.98	5.92E+06	-
M4	PU-HZ151 + 14 amu	NP	0	0	NP	0	0

NP= not present Total peak area of metabolites: 3.16E+07 Total peak area of metabolites: 4.13E+07

Peak ID/Rat	Proposed identity	R.T. (min)	Peak area#1	% Peak area of metabolite	R.T. (min)	Peak area#2	% Peak area of metabolite
M5	Desneopentyl + Desmethylene PU-HZ151	4.38	2.26E+06	9.5	4.41	2.33E+06	8.3
M1	Desneopentyl PU-HZ151	4.97	7.36E+06	31.0	5.00	8.02E+06	28.7
M2	Desmethylene PU-HZ151	5.40	5.71E+06	24.0	5.39	7.38E+06	26.4
M3a	PU-HZ151+ Oxygen	5.59	7.61E+06	32.0	5.59	9.17E+06	32.8
M3b	PU-HZ151+ Oxygen	5.72	6.69E+05	2.8	5.72	8.85E+05	3.2
PU-HZ151	PU-HZ151	6.01	1.23E+07	-	6.02	1.51E+07	-
M4	PU-HZ151+ 14 amu	6.92	1.64E+05	0.7	6.89	1.54E+05	0.5

Total peak area of metabolites: 2.38E+07 Total peak area of metabolites: 2.79E+07

Peak ID/Dog	Proposed identity	R.T. (min)	Peak area#1	% Peak area of metabolite	R.T. (min)	Peak area#2	% Peak area of metabolite
M5	Desneopentyl + Desmethylene PU-HZ151	4.42	3.49E+05	0.9	4.41	2.18E+05	0.6
M1	Desneopentyl PU-HZ151	5.01	1.72E+07	46.5	5.00	1.76E+07	47.5
M2	Desmethylene PU-HZ151	5.42	1.28E+07	34.7	5.41	1.23E+07	33.2
M3a	PU-HZ151+ Oxygen	5.61	3.34E+05	0.9	5.60	3.52E+05	1.0
M3b	PU-HZ151+ Oxygen	5.73	4.98E+06	13.5	5.73	5.29E+06	14.3
PU-HZ151	PU-HZ151	6.02	7.39E+07	-	6.92	7.61E+07	-
M4	PU-HZ151+ 14 amu	6.93	1.25E+06	3.4	6.02	1.27E+06	3.4

Total peak area of metabolites: 3.69E+07 Total peak area of metabolites: 3.71E+07

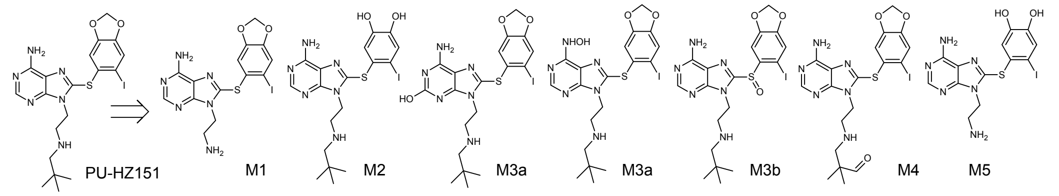

Metabolite characterization of samples collected from 10 μM PU-HZ151 incubations after 60 min in human microsomes revealed the presence of six metabolites. The putative identities are as follows: M1: desneopentyl PU-HZ151 (-70 amu), M2: desmethylene PU-HZ151 (-12 amu), M3a and M3b: PU-HZ151 + Oxygen (+16 amu), M4: aldehyde form of PU-HZ151 (+14 amu), and M5: desneopentyl + desmethylene PU-HZ151 (-82 amu). These six metabolites were also observed in rat and dog microsomes. Only three metabolites were observed in mouse microsomes; M1: desneopentyl PU-HZ151 (-70 amu), M2: desmethylene PU-HZ151 (-12 amu), and M5: desneopentyl + desmethylene PU-HZ151 (-82 amu). Table: Percent peak area of metabolite versus Total peak area of metabolites in human, mouse, rat, and dog microsomal samples at 60 min in replicate samples, n = 2. Peak area#1, replicate 1; Peak area#2, replicate 2. The proposed structure of metabolites is also shown.

Supplementary Table 10.

		Patient#1	Patient#2	Patient#3	Mean	SD	
Bi-exponential Fit to Plasma Time-Activity Data							
1st Component	Intercept:	0.0203	0.0283	0.00856	0.01905	0.00994	%ID g^{-1}
	Clearance Constant:	16.0	20.3	14.0	16.8	3.21	h^{-1}
	Clearance Half-time:	0.0434	0.0342	0.0495	0.0424	0.00772	h
		2.60	2.05	2.97	2.54	0.463	min
2nd Component	Intercept2:	0.00787	0.00432	0.00380	0.00533	0.00222	%ID g^{-1}
	Clearance Constant:	0.370	0.2332	0.2691	0.291	0.0708	h^{-1}
	Half-time:	1.87	2.97	2.21	2.35	0.563	h
		112	178	132	141	33.8	min
	1. Pharmacokinetic profile of [^{124}I]-PU-HZ151: area under the curve (AUC) [Time Frame: 1 week]	0.0225	0.0199	0.0127	0.0184	0.00509	(%ID g^{-1})h
	2. Pharmacokinetic profile of [^{124}I]-PU-HZ151: maximum plasma concentration (C_{max}) [Time Frame: 1 week]	0.0281	0.0326	0.0124	0.0244	0.0107	%ID g^{-1}
	3. Pharmacokinetic profile of [^{124}I]-PU-HZ151: trough plasma concentration (C_{min}) [Time Frame: 1 week]	8.20E-30	4.16E-20	8.84E-23	1.39E-20	2.40E-20	%ID g^{-1}
		0	0	0	0		
	4. Pharmacokinetic profile of [^{124}I]-PU-HZ151: plasma half-life ($T_{1/2}$) [Time Frame: 1 week]						
	1st Component	0.0434	0.0342	0.0495	0.0424	0.00772	h
	2nd Component	1.87	2.97	2.21	2.35	0.563	h
	5. Pharmacokinetic profile of [^{124}I]-PU-HZ151: time to maximum plasma concentration (T_{max}) [Time Frame: 1 week]	0	0	0	0	0	h

Pharmacokinetic profile of [^{124}I]-PU-HZ151 in human patients. Primary outcome measures from NCT03371420, PET Imaging of Subjects Using 124I-PU-AD, a first in-human, open-label pilot (microdose) study of the positron-emitting agent [^{124}I]-PU-HZ151 (clinical name 124I-PU-AD) in subjects with specific cancer types (solid malignancy, lymphoma, and/or myeloma) and/or Alzheimer's disease. Plasma clearance of [^{124}I]-PU-HZ151was best-fit by a biexponential clearance model with rapid initial clearance (2.5 +/- 0.5 minutes) and slow terminal phase clearance (141.0 +/- 33.8 minutes), with AUC 0.0184 +/- 0.00509 %ID g^{-1} h, C_{max} of t = 0 (bolus intravenous injection) and plasma concentrations becoming negligible (C_{min} = 0 %ID mL^{-1}) within study time-frame.

Supplementary Table 11.

ID	Histology	Subtype	IHC	Cytogenetics	EGFRvIII	MGMT
#1	Rec/res HG glioma*	Proneural				
#2	Rec/res GBM	Mesenchymal		EGFR amp (-)		(-)
#3	GBM	Proneural		EGFR amp (-)	(-)	(+)
#4	Rec/res HG glioma*			EGFR amp (+)		
#5	GBM			EGFR amp (+)	(-)	
#6	GS			EGFR amp (-)	(+)	(-)
#7	GBM			EGFR amp (+)	(-/+)	(+)
#8	GBM	Proneural				
#9	GBM	Mesenchymal		EGFR amp (-)	(-)	(-)
#10	GBM with OD component			EGFR amp (-), 1p19q del (-)		(+)
#11	Rec/res HG glioma*					
#12	GS	Mesenchymal		EGFR amp (-)	(-)	(-)
#13	GBM			EGFR amp (-)	(-)	(-)
#14	Rec/res HG glioma*					
#15	GBM	Mesenchymal		EGFR amp (-)	(-)	(-)
#16	GBM	Classical		EGFR amp (+)	(-)	(-)
#17	GBM	Proneural		EGFR amp (-)	(-)	(-)
#18	GBM	Mesenchymal		EGFR amp (-)	(-)	(-)
#19	GBM	Mesenchymal		EGFR amp (-)	(-)	(+)
#20	Rec/res HG glioma	Proneural	IDH1_R132H (-)	EGFR amp (-)	(-)	
#21	GBM	Mesenchymal		EGFR amp (-)	(-)	(-)
#23	GBM	Proneural		EGFR amp (-)	(-)	(-)
#24	GBM	Proneural				
#25	GBM	Classical		EGFR amp (+)	(+)	(+)
#26	GBM		PTEN (-), IDH1_R132H (-)	EGFR amp (+)	(-)	(+)
#27	Rec/res GBM*	Classical	IDH1_R132H (-)			
#28	Anaplastic OD		IDH1_R132H (+)	1p19q del (+)		
#29	GBM		IDH1_R132H (-)	EGFR amp (-), 1p del (-), 19q del (+)	(-)	(+)
#30	GBM				(+)	(-)
#31	GBM	Classical	PTEN (+), IDH1_R132H (-)	EGFR amp (+)	(+)	(+)
#32	Rec/res GBM	Mesenchymal		EGFR amp (-)	(-)	(-)
#33	GBM with OD component	Proneural	IDH1_R132H (-)	EGFR amp (-), 1p19q del (-)	(+)	(-)
#34	GBM	Classical		EGFR amp (+)	(-)	(-)
#35	Rec/res HG glioma*					
#36	Rec/res GBM	Proneural	PTEN (-), IDH1_R132H (-)	EGFR amp (-)	(-)	(-)

Characteristics of the primary specimens used in this study. Abbreviations: GBM, glioblastoma multiforme; GS, gliosarcoma; HG, high grade; OD, oligodendroglioma; rec/res, recurrent/resistant. MGMT, O6-methylguanine-DNA methyltransferase

Supplementary Table 12.

Subject Inclusion Criteria	Subject Exclusion Criteria
Subject is between 21-90 years old at time of consent.	Subject has history of allergic reaction to X-ray CT iodinated contrast medium and/or hypersensitivity to iodide products.
Subject has negative serum pregnancy test for females of childbearing age (11-50 years) or lacks child-bearing potential.	Subject has known hyperthyroidism.
Subjects with a diagnosis of cancer and/or Alzheimer's Disease, meeting trial eligibility criteria as specified below for either disease.<br><br>For subjects with cancer:<br><br>a) Subjects with eligible histologic type of cancer. Eligible histologic types of cancer include solid malignancy, myeloma, and lymphoma.<br>b) Cancer histology confirmed by pathology.<br>c) Cancerous disease is radiologically-measurable or evaluable as defined by published tumor response criteria (including, but not limited to RECIST 1.1)<br><br>For subjects with Alzheimer's Disease:<br>d) Established diagnosis of mild-moderate Alzheimer's disease based upon neurological and neuropsychological evaluation following the National Institute on Aging – Alzheimer's disease Association criteria that revisited the NINCDS-ADRDA criteria.*<br>e) Documentation of diagnosis of mild-moderate Alzheimer's disease, as above, by board-certified neurologist.<br>f) Subject has an appointed health care proxy specifically designated for research consent and this appointment is documented.<br>g) Subject has designated at-home caregiver(s) responsible for providing daily medication to the patient, who will document the patient's daily doses of SSKI oral medication, for 2 weeks as per study protocol.	Subject has inability to give consent personally or via appointed health care proxy.<br><br>Women who are pregnant or breastfeeding (and for 1 month after receiving the study drug).<br><br>Subject has any other condition or laboratory abnormality or receives any other treatment(s) that may increase the risk associated with study participation or may interfere with the interpretation of study results in the judgment of the investigator.<br><br>Subject has concurrent participation in any interventional studies within 30 days of first dose of study drug<br><br>Subject has history of acute major illness (i.e., unstable cardiovascular condition.)
Subjects who have both cancer and Alzheimer's Disease, subjects are considered eligible if they meet all eligibility requirements for either Alzheimer's Disease or cancer patients, as specified above.	Subject has unacceptable pre-study organ function during screening defined as:<br>a. Bilirubin > 1.5 x institutional upper limit of normal (ULN)<br>b. AST/ALT >2.5 x ULN<br>c. Albumin < 2 g dl^{-1}<br>d. GGT > 2.5 x ULN (IF Alkaline phosphatase > 2.5 x ULN).<br>e. Creatinine >1.5 x ULN or creatinine clearance < 60 mL min^{-1}.

Patient eligibility criteria. Patient history, physical exam, complete blood count (CBC) and liver and renal function laboratory tests were performed < 2 weeks prior to study entry. For toxicity monitoring, serum thyroid-stimulating hormone (TSH) was also obtained < 2 weeks prior to study entry and at 6-12 months follow-up.
*McKhann, et al. The Diagnosis of Dementia Due to Alzheimer's Disease: Recommendations from the National Institute on Aging – Alzheimer's Association Workgroups on Diagnostic Guidelines for Alzheimer's Disease. Alzheimers Dement 2011;May;7(3):263-9. doi: 10.1016/j.jalz.2011.03.005.

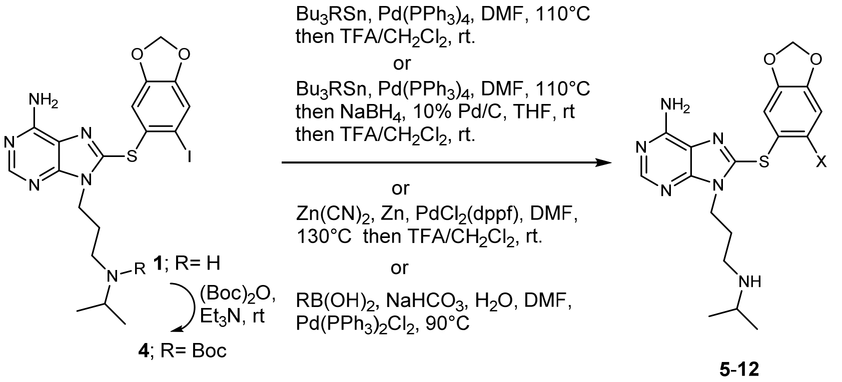

Supplementary Fig. 1: Synthesis of compound 5-12. The cyano derivative **5** was prepared from **4** by palladium catalyzed reaction with $Zn(CN)_2/Zn$ following deprotection with TFA. **6** was synthesized from Stille coupling of **1** with tri-*n*-butyl(1-ethoxy-vinyl)tin followed by hydrolysis. S-linker derivatives **7** and **8** were prepared from Stille coupling of Boc-protected purine **4** with tributylvinyltin or tributylallyltin, respectively, following removal of the Boc-group. Phenyl **9** and substituted phenyl derivatives **10-12** were prepared by direct Suzuki coupling of **1** with various boronic acids. Boc, *tert*-butoxycarbonyl; TFA, trifluoroacetic acid.

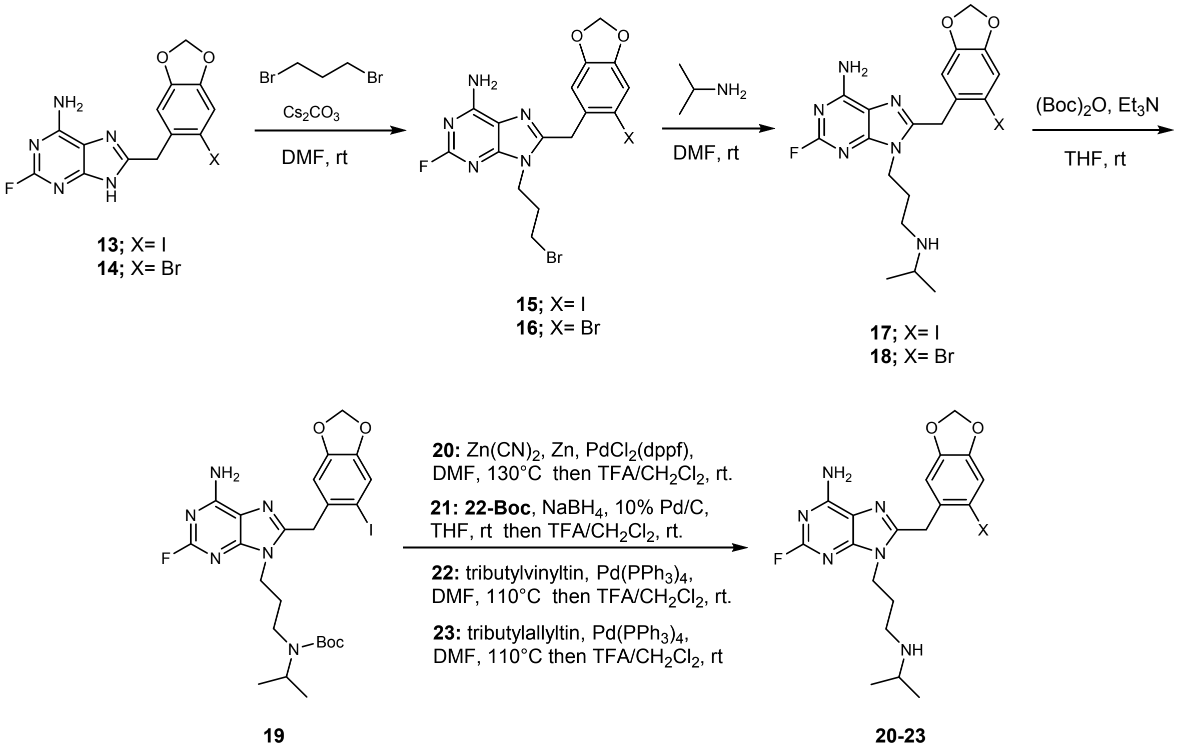

Supplementary Fig. 2: Synthesis of compounds 17, 18 & 20-23. **17** was synthesized by *N*9-alkylation of **13** with 1,3-dibromopropane followed by reaction of the resulting bromide **15** with isopropylamine. **14** was *N*9-alkylated with 1,3-dibromopropane to **16** in 86% yield which was reacted with isopropylamine to give **18** in 70% yield. Similarly, **17** was Boc-protected with di-*tert*-butyl dicarbonate to give **19** in 85% yield. The palladium catalyzed reaction of **19** with $Zn(CN)_2$/Zn resulted in cyano **20** following deprotection with TFA. Reduction of Boc-protected **22** with $NaBH_4$ and 10% Pd/C resulted in ethyl derivative **21**, following removal of the Boc-group. **19** was subjected to Stille coupling with tributylvinyltin or tributylallyltin to give **22** and **23**, respectively, following removal of the Boc-group with TFA. Boc, *tert*-butoxycarbonyl; TFA, trifluoroacetic acid; $(Boc)_2O$, di-*tert*-butyl decarbonate.

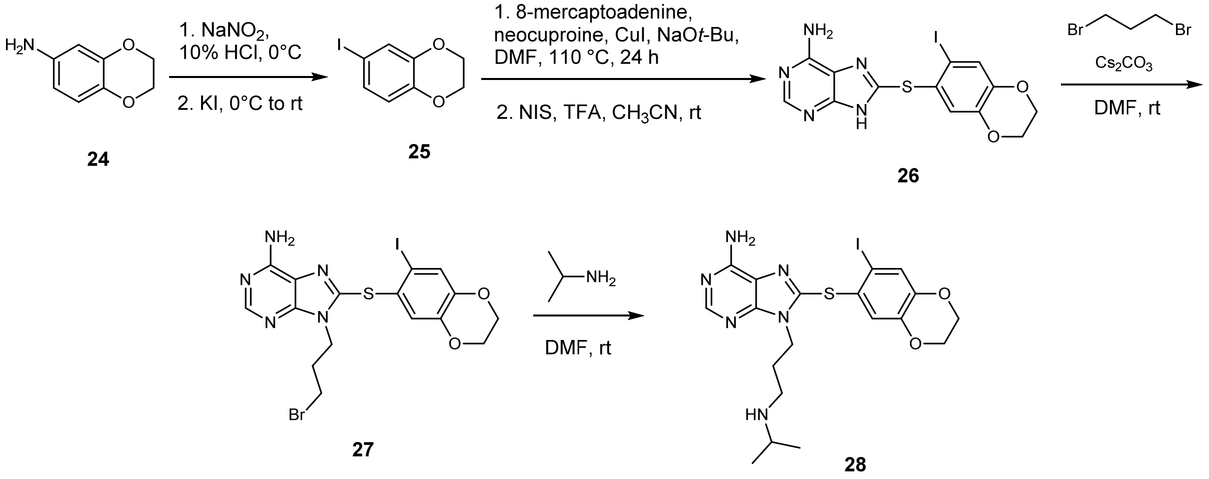

Supplementary Fig. 3: Synthesis of ethylenedioxy derivative 28. Amine **24** was converted to iodine **25** by iodo-de-diazoniation in 86% yield. **25** was coupled with 8-mercaptoadenine using CuI/neocuproine to give a thioether intermediate in 80% yield which was iodinated with NIS/TFA to give **26** in 73% yield. *N*9-alkylation of **26** with 1,3-dibromopropane followed by reaction of the resulting bromide **27** with isopropylamine resulted in ethylenedioxy derivative **28**. NIS, *N*-iodosuccinimide; TFA, trifluoroacetic acid.

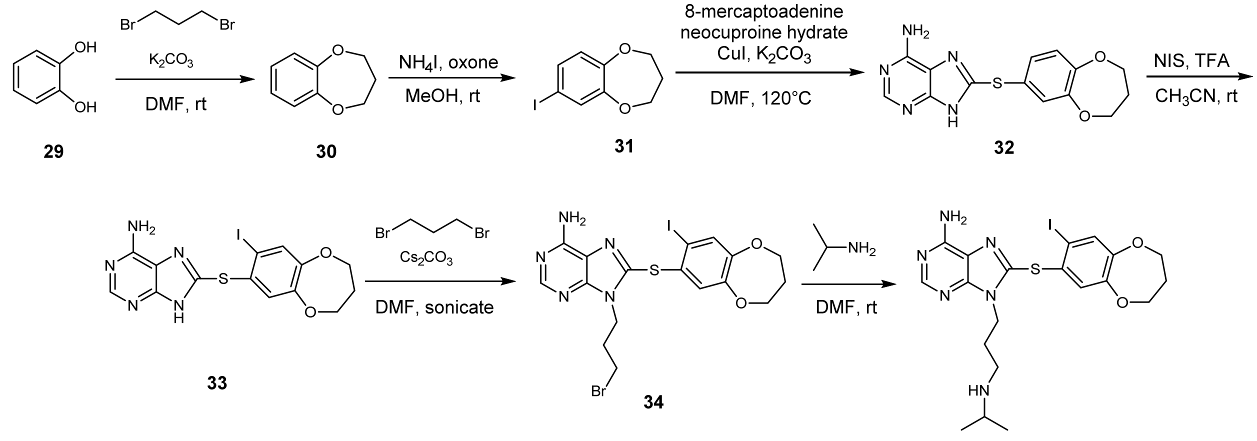

Supplementary Fig. 4: Synthesis of propylenedioxy derivative 35. 31 was obtained from the cyclization of catechol (**29**) with 1,3-dibromopropane in 44% yield followed by reaction of **30** with ammonium iodide/oxone in 70% yield. **31** was then coupled with 8-mercaptoadenine using CuI/neocuproine to give **32** in 56% yield which was then iodinated with NIS to give **33** in 70% yield. *N*9-alkylation of **33** with 1,3-dibromopropane followed by reaction of the resulting bromide **34** with isopropylamine resulted in **35**. NIS, *N*-iodosuccinimide; TFA, trifluoroacetic acid.

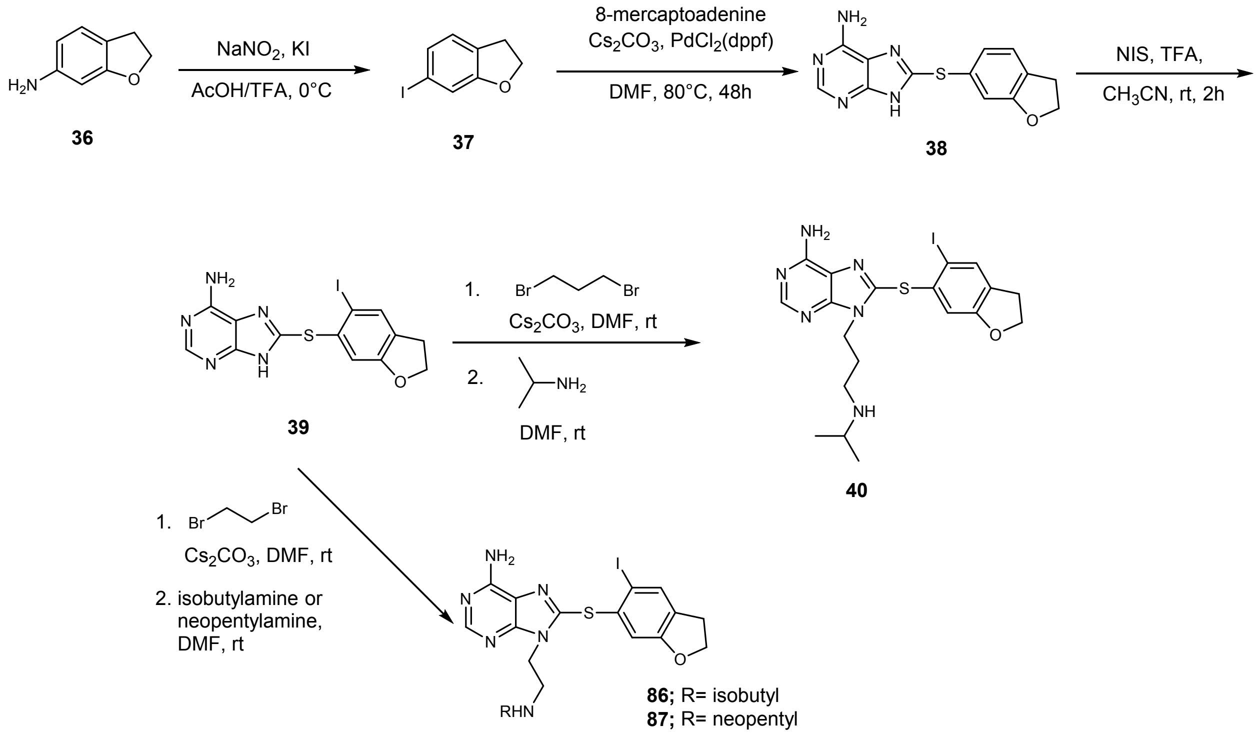

Supplementary Fig. 5: Synthesis of 40, 86 and 87. Amine **36** was converted to iodine **37** by iodo-de-diazoniation in 61% yield. **37** was coupled with 8-mercaptoadenine using $PdCl_2$(dppf) to give **38** in 44% yield which was iodinated with NIS/TFA to give **39** in 53% yield. *N*9-alkylation of **39** with 1,3-dibromopropane followed by reaction of the resulting bromide with isopropylamine resulted in **40**. *N*9-alkylation of **39** with 1,2-dibromoethane followed by reaction of the resulting bromide with isobutylamine resulted in **86** or with neopentylamine resulted in **87**. AcOH, acetic acid; NIS, *N*-iodosuccinimide; TFA, trifluoroacetic acid.

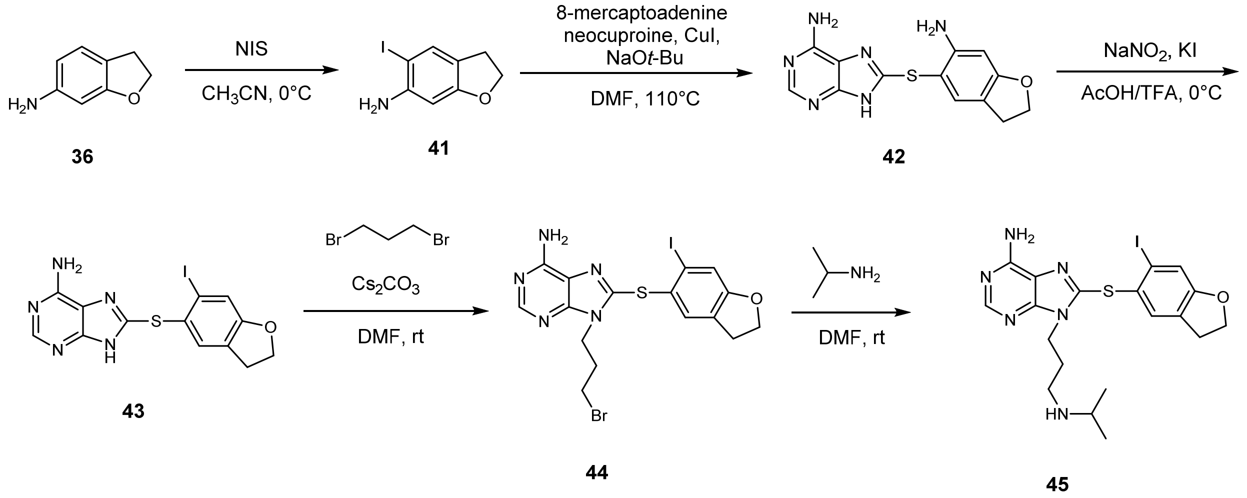

Supplementary Fig. 6: Synthesis of 45. Amine **36** was iodinated with NIS to give **41** in 98% yield. **41** was coupled with 8-mercaptoadenine using neocuproine/CuI to give **42** in 56% yield which was converted to iodine **43** by iodo-de-diazoniation in 41% yield. *N*9-alkylation of **43** with 1,3-dibromopropane followed by reaction of the resulting bromide **44** with isopropylamine resulted in **45**. AcOH, acetic acid; NIS, *N*-iodosuccinimide; TFA, trifluoroacetic acid.

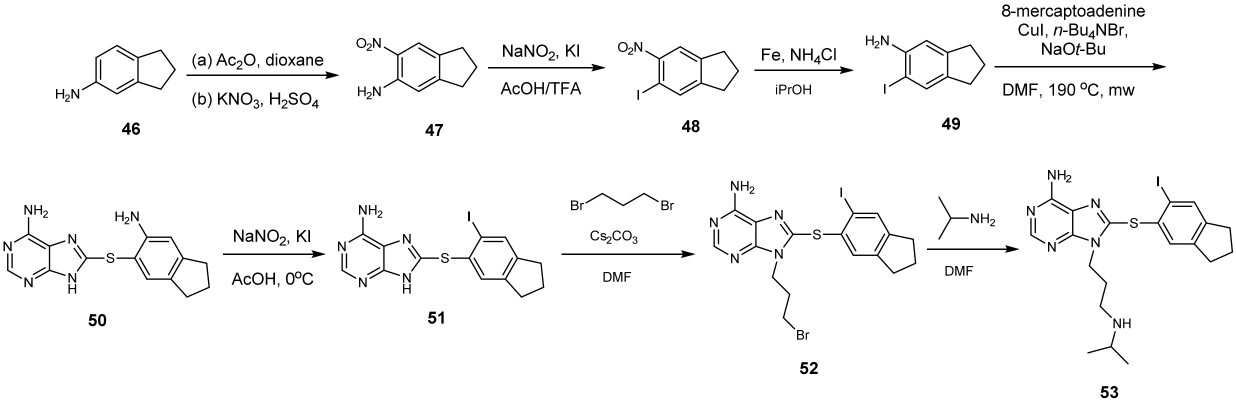

Supplementary Fig. 7: Synthesis of 53. 5-Aminoindane (**46**) was first treated with acetic anhydride followed by reaction with KNO_3/H_2SO_4 to give **47** in 43% yield. **47** was converted to iodine **48** by iodo-de-diazoniation in 65% yield which was then reduced with iron powder to give **49** in 92% yield. **49** was coupled with 8-mercaptoadenine using neocuproine with microwave to give **50** in 47% yield which was then converted to iodine **51** by iodo-de-diazoniation in 56% yield. *N*9-alkylation of **51** with 1,3-dibromopropane followed by reaction of the resulting bromide **52** with isopropylamine resulted in **53**. Ac_2OH, acetic anhydride; AcOH, acetic acid; NIS, *N*-iodosuccinimide; TFA, trifluoroacetic acid; *n*-Bu_4NBr, tetra-*n*-butylammonium bromide.

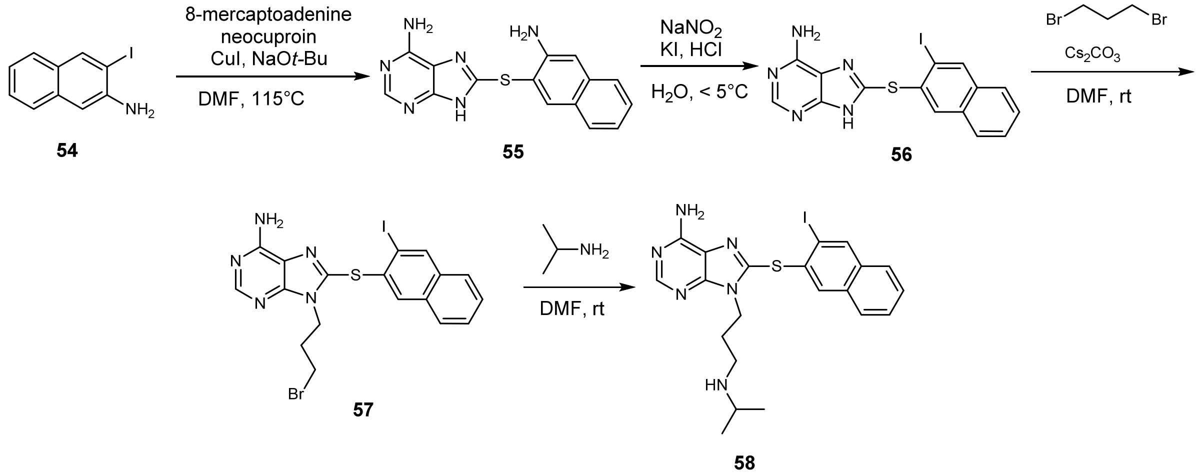

Supplementary Fig. 8: Synthesis of naphthalene derivative 58. 54 was coupled with 8-mercaptoadenine using CuI/neocuproine to give **55** in 37% yield which was then converted to **56** by iodo-de-diazoniation in 42% yield. *N*9-alkylation of **56** with 1,3-dibromopropane followed by reaction of the resulting bromide **57** with isopropylamine resulted in **58**.

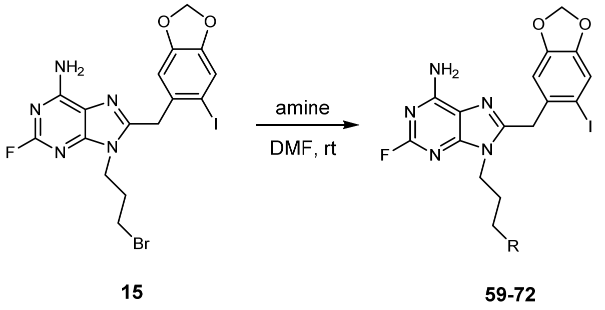

Supplementary Fig. 9: Synthesis of 59-72. Compounds **59-72** were prepared by the amination of bromide **15** with various corresponding amines.

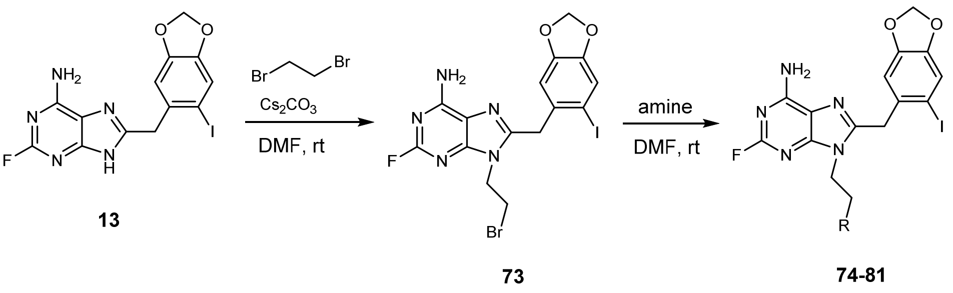

Supplementary Fig. 10: Synthesis of 74-81. Compounds **74-81** were prepared by the reaction of a variety of amines with pendant bromide **73**, which in turn was prepared by the alkylation of **13** with 1,2-dibromoethane in 44% yield.

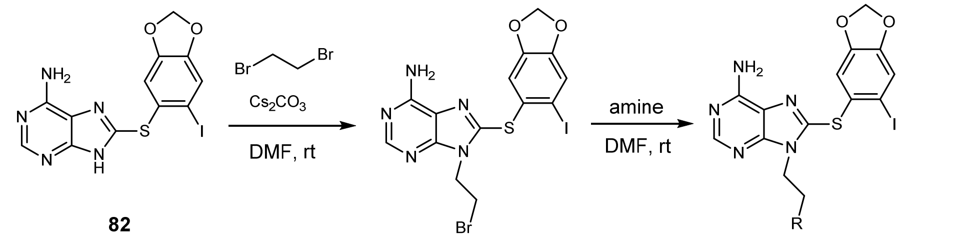

82

83

84; R= isobutylamine
85; R= neopentylamine

Supplementary Fig. 11: Synthesis of compounds 84 & 85. Compounds **84 & 85** were prepared by reaction of **83** with isobutylamine or neopentylamine, respectively.

a

b

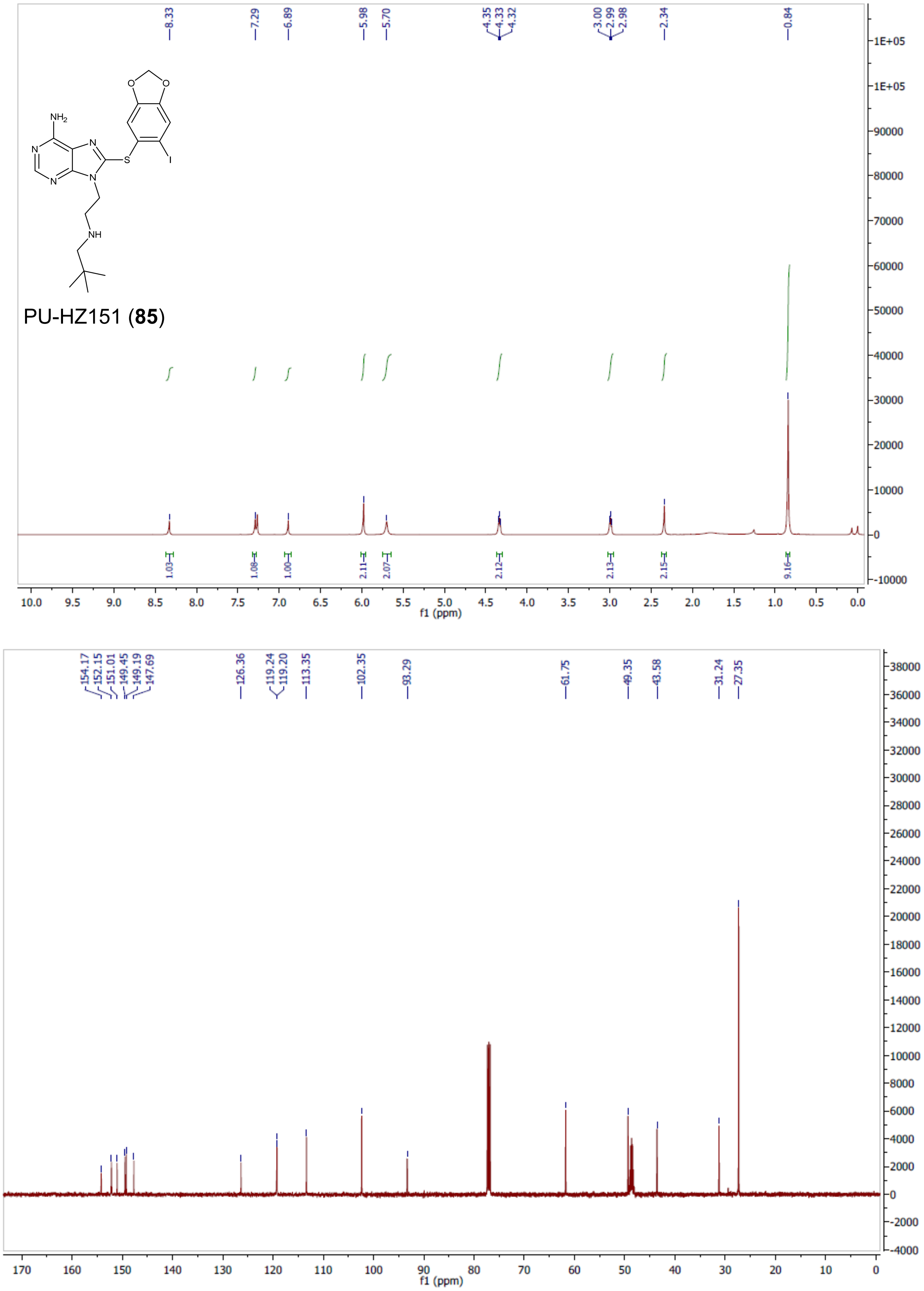

Supplementary Fig. 12: Structural characterization of PU-HZ151 (85) by nuclear magnetic resonance spectroscopy. a ^{1}H NMR spectra in $CDCl_3$. **b** ^{13}C NMR spectra in $CDCl_3/CD_3OD$.

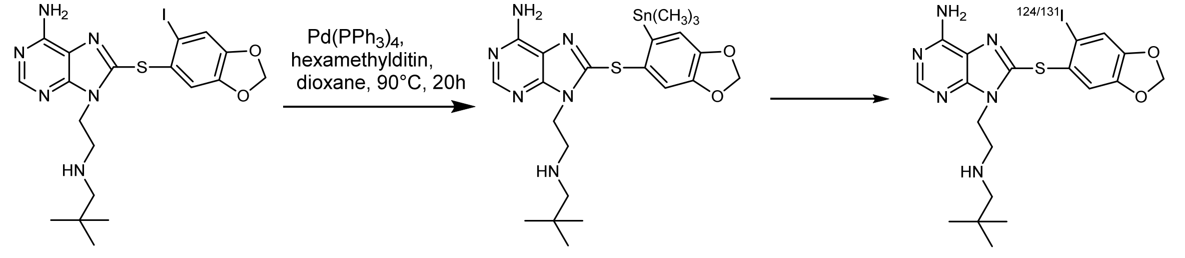

85 **PU-HZ151-Sn** **$[^{(124/131)}I]$-PU-HZ151**

Supplementary Fig. 13: Synthesis of $[^{(124/131)}I]$-PU-HZ151. PU-HZ151-Sn precursor was prepared by the Pd-catalyzed reaction of 85 with hexamethylditin which was then radiolabelled with 124/131-NaI.

a

Target	SKI-8
Gene Symbol	%Ctrl @ 10000nM
ABL1(E255K)-phosphorylated	80
ABL1(T315I)-phosphorylated	96
ABL1-nonphosphorylated	50
ABL1-phosphorylated	70
ACVR1B	83
ADCK3	92
AKT1	93
AKT2	87
ALK	93
AURKA	96
AURKB	73
AXL	100
BMPR2	97
BRAF	100
BRAF(V600E)	97
BTK	100
CDK11	90
CDK2	98
CDK3	100
CDK7	79
CDK9	91
CHEK1	100
CSF1R	94
CSNK1D	91
CSNK1G2	100
DCAMKL1	78
DYRK1B	36
EGFR	96
EGFR(L858R)	93
EPHA2	91
ERBB2	100
ERBB4	100
ERK1	100
FAK	96
FGFR2	63
FGFR3	95
FLT3	100
GSK3B	88
IGF1R	100
IKK-alpha	99
IKK-beta	88
INSR	92
JAK2(JH1domain-catalytic)	100
JAK3(JH1domain-catalytic)	86
JNK1	96
JNK2	93
JNK3	100
KIT	99

Target	SKI-8
Gene Symbol	%Ctrl @ 10000nM
KIT(D816V)	93
KIT(V559D,T670I)	96
LKB1	81
MAP3K4	49
MAPKAPK2	96
MARK3	100
MEK1	99
MEK2	76
MET	100
MKNK1	100
MKNK2	93
MLK1	100
p38-alpha	100
p38-beta	89
PAK1	92
PAK2	97
PAK4	100
PCTK1	100
PDGFRA	100
PDGFRB	98
PDPK1	77
PIK3C2B	96
PIK3CA	87
PIK3CG	94
PIM1	79
PIM2	81
PIM3	91
PKAC-alpha	87
PLK1	97
PLK3	88
PLK4	89
PRKCE	100
RAF1	74
RET	100
RIOK2	94
ROCK2	97
RSK2(Kin.Dom.1-N-terminal)	84
SNARK	100
SRC	100
SRPK3	100
TGFBR1	63
TIE2	100
TRKA	95
TSSK1B	51
TYK2(JH1domain-catalytic)	99
ULK2	94
VEGFR2	98
YANK3	95
ZAP70	78

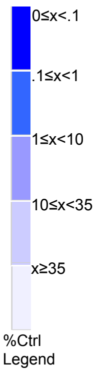

b

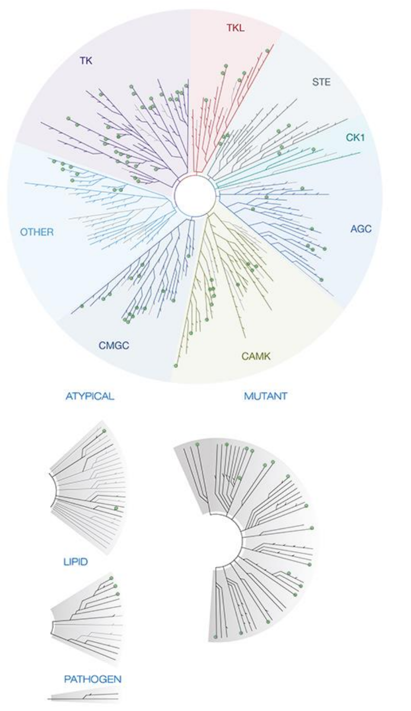

97 Assays Tested
0 Interactions Mapped
S-score(35) = 0

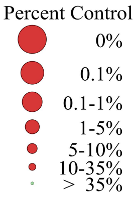

Supplementary Fig. 14: PU-HZ151 shows no off-target binding to kinases in vitro. a PU-HZ151 (at 10 μM) was tested in the scanEDGE screen against 97 kinases. Comprehensive list of the 97 kinases screened in the scanEDGE screen is shown. Lower numbers indicate stronger hits in the matrix. **b** The TREEspot interaction map for PU-HZ151 is presented. KINOMEscan's selectivity score (S) is a quantitative measure of compound selectivity. It is calculated by dividing the number of kinases that bind to the compound by the total number of distinct kinases tested, excluding mutant variants. S(35) = (number of nonmutant kinases with %Ctrl < 35)/(number of nonmutant kinases tested).

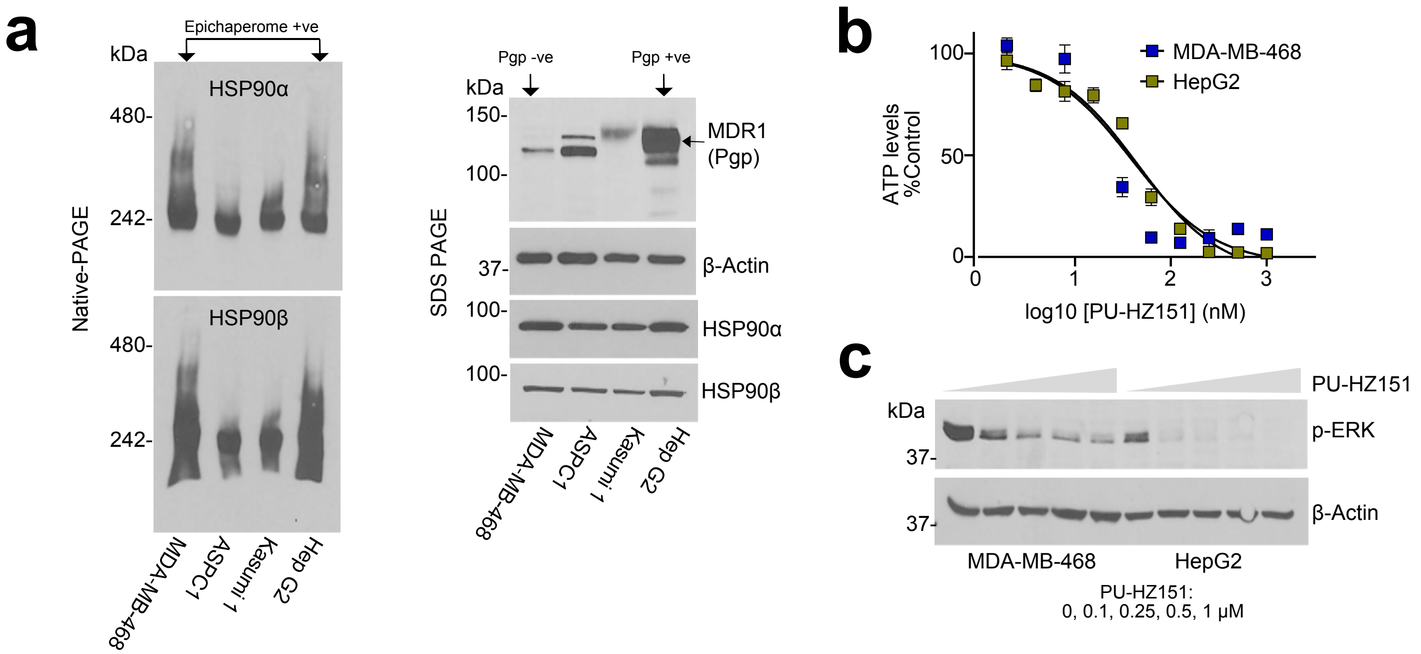

Supplementary Fig. 15: PU-HZ151 activity in cells is unaffected by Pgp overexpression. **a** Two Pgp-positive cells lines, Kasumi-1 and HepG2, were tested for epichaperome-positivity. β-Actin, loading control. **b** ATP levels in cell treated for 72 h with 0 to 1 μM PU-HZ151. Data are presented as means ±s.e.m. of three experimental repeats. Control, vehicle (DMSO) treated cells. **c** Western blot analysis of aberrant signaling pathway inhibition by PU-HZ151. Source data are provided as a Source Data file.

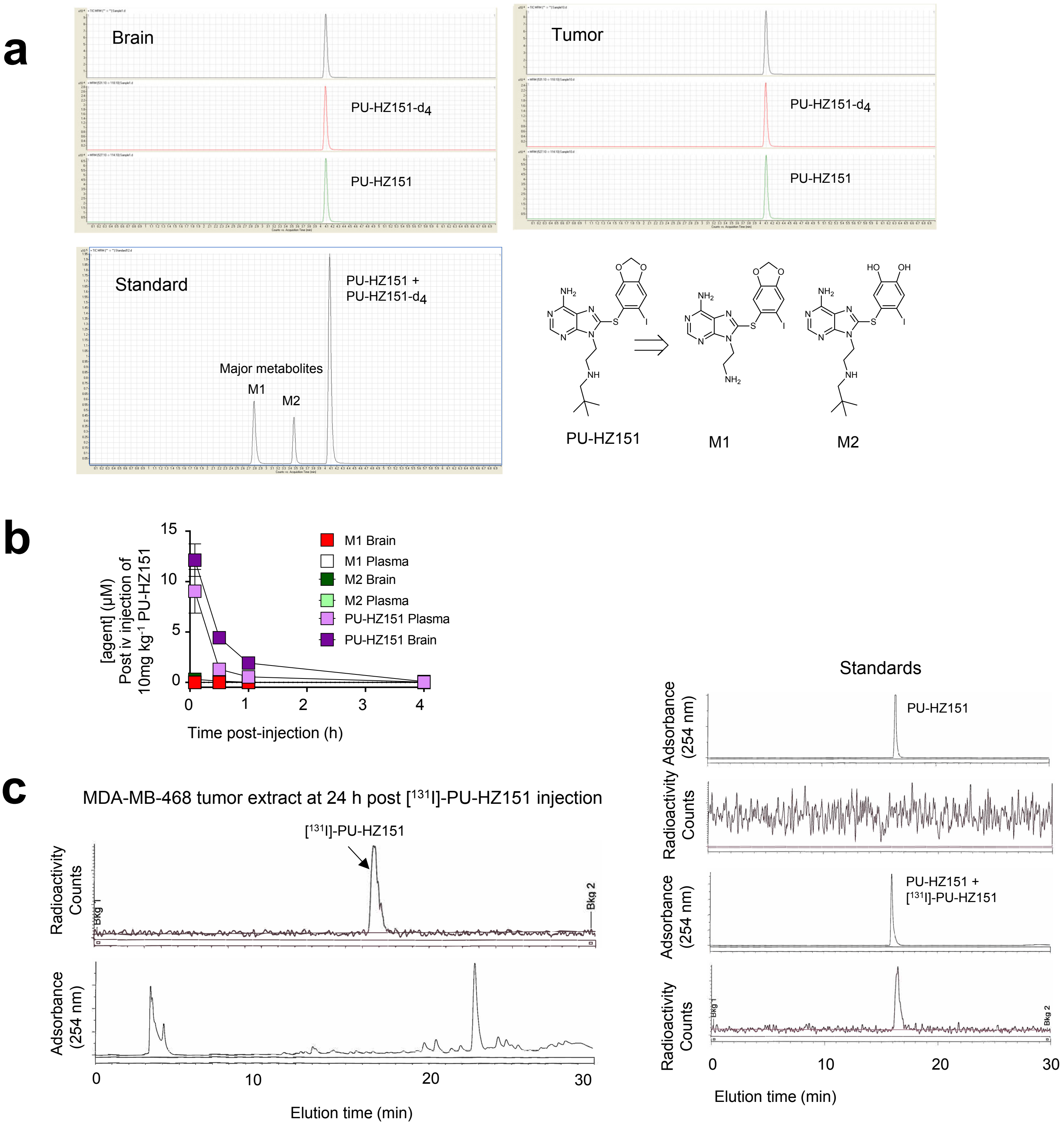

Supplementary Fig. 16: Liquid chromatography-tandem mass spectrometry (LC-MS/MS) and radio-HPLC confirm intact PU-HZ151 as the single species present in epichaperome-positive tissue and non-metabolizing organs. a Representative LC-MS/MS profiles of PU-HZ151 extracted from brain and from tumors at 1 h post-administration of 20 mg kg^{-1} PU-HZ151 (n = 5), show intact compound as the single species present. Deuterated PU-HZ151 is used as internal standard. **b** Molar concentration of PU-HZ151 in the indicated tissues and organs at 48 h after single-dose intraperitoneal (ip) administration of 75 mg kg^{-1} PU-HZ151, n = 4 mice. Data are presented as mean ± s.e.m.. **c** HPLC and radio-HPLC chromatograms of MDA-MB-468 tumor extracts of mice (n = 4) injected [^{131}I]-PU-HZ151 (1 mCi) intravenously and sacrificed at 24 h post-injection. Standards are shown on the right. Source data are provided as a Source Data file.

a

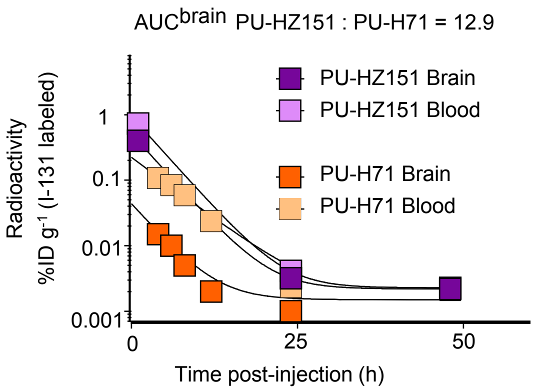

b

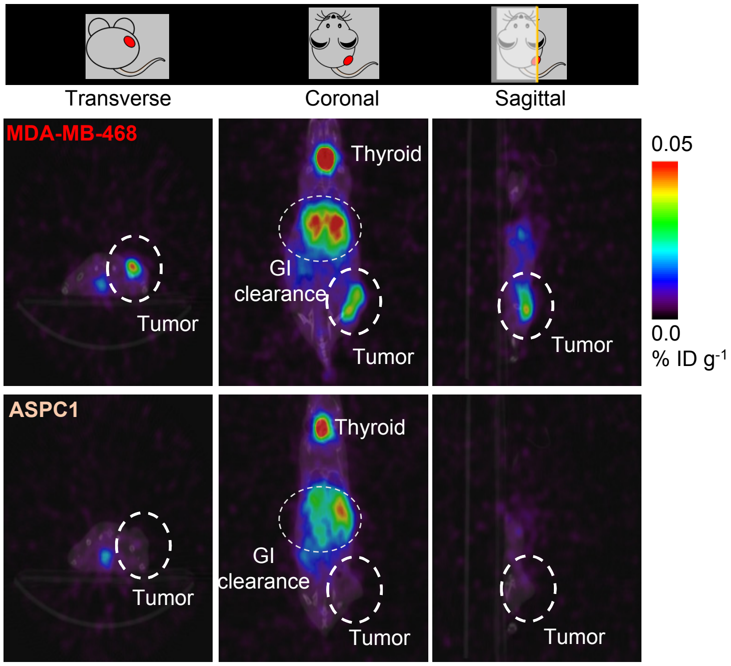

Supplementary Fig. 17: Biodistribution of PU-HZ151 and PU-H71 in mice. a Time-dependent radioactivity curve of [^{131}I]-PU-HZ151 and [^{131}I]-PU-H71 in plasma and brain. n = 5 mice per- time point. Data are presented as means ± s.e.m.. **b** PET image of representative mice obtained at 48 h post single [^{124}I]-PU-H71 injection (250 µCi; 12.5 µCi g^{-1}). Source data are provided as a Source Data file.

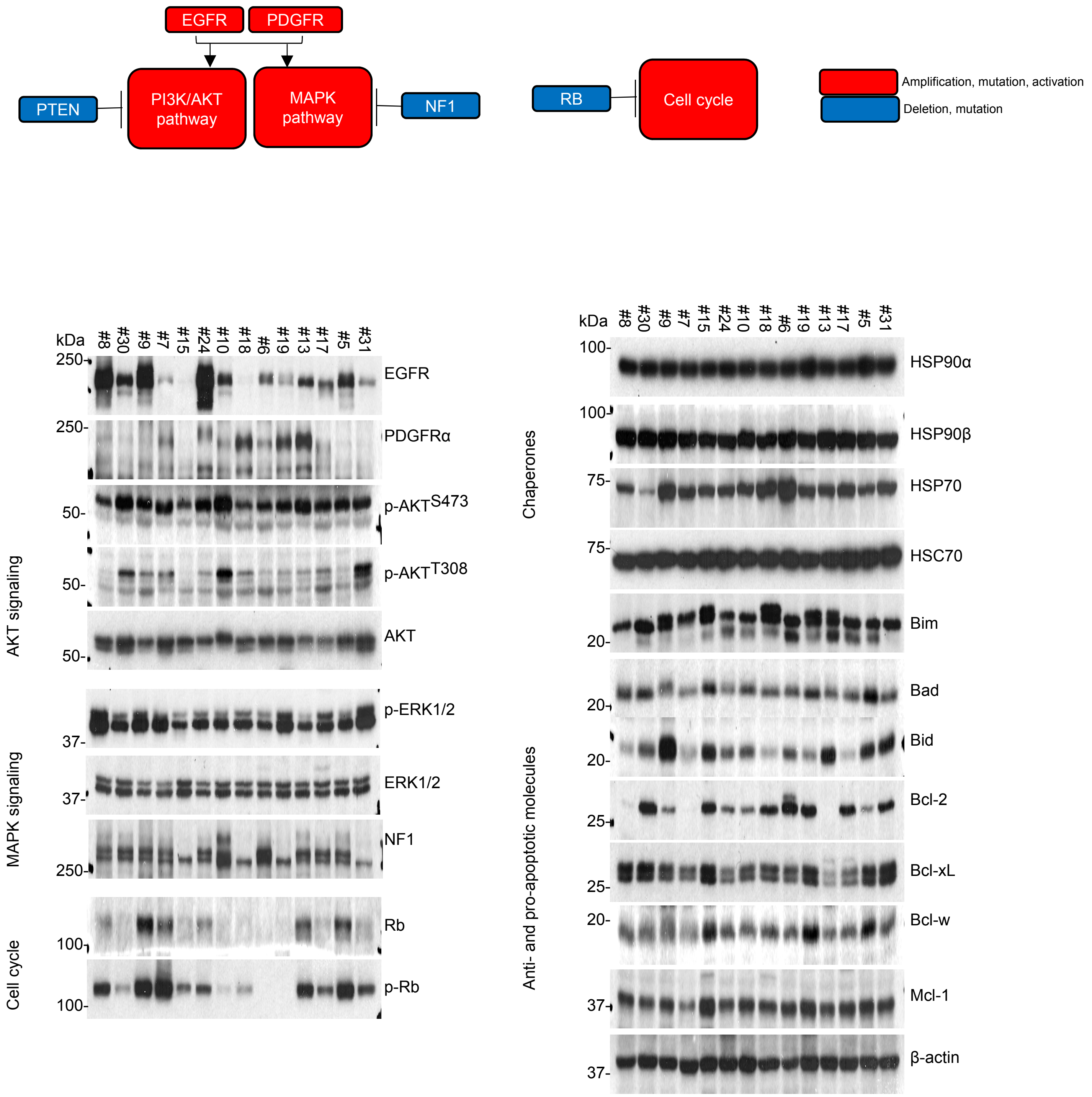

Supplementary Fig. 18: GSCs capture the molecular features and heterogeneity of human GBM tumors. Western blot analysis of molecular markers in patient-derived GSCs cultured adherently in a serum-free stem cell culture condition. The sample set captures the molecular diversity observed in GBMs (Brennan et al. Cancer Cell 155(2):462 2013) (eg. overexpression or truncations of EGFR and PDGFR, activation of their downstream signaling pathways such as PI3K/AKT and MAPK, and impairment in cell cycle regulatory proteins (i.e. retinoblastoma protein loss).

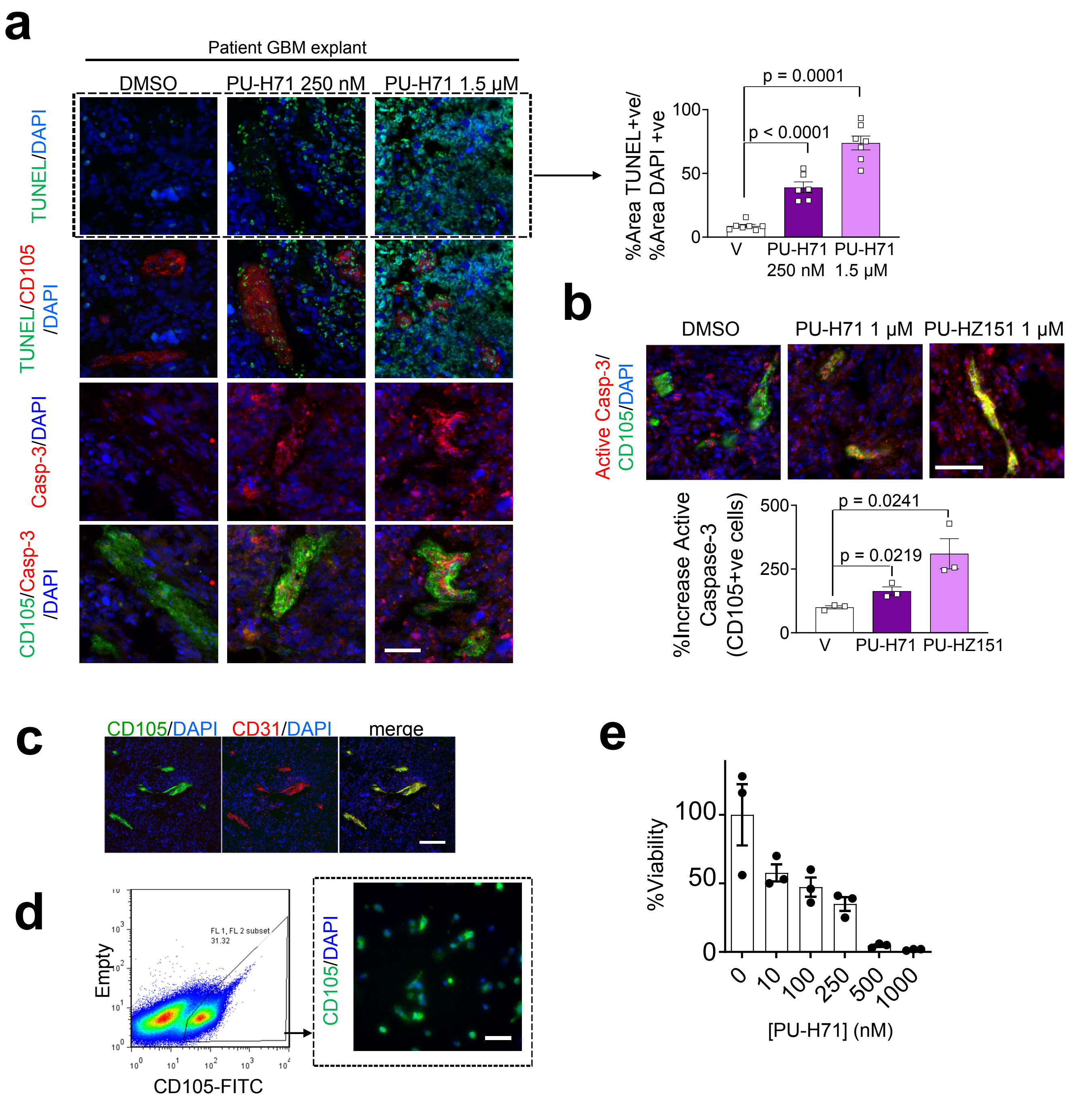

Supplementary Fig. 19: The GBM tumor endothelial cells are vulnerable to agents that induce epichaperome disassembly. a Representative GBM explants treated with PU-H71 at the indicated concentrations for 10 days were analyzed by immunohistochemistry (IHC) using an endothelial cell (EC) marker (CD105) and apoptosis markers (cleaved caspase-3 and TUNEL). Apoptotic nuclei are observed in both tumor cells and ECs. Graph, mean±SEM, n = 7, 6 and 7 explants, respectively, one-way ANOVA with Dunnett's post hoc, $F(2,17) = 68$. Scale bar, 50 μm. **b** Same as in (**a**) for quantification of apoptotic ECs in explants treated with vehicle, PU-H71 or PU-HZ151. Graph, mean±SEM, n = 3 explants, multiple unpaired two-tailed t-test. Scale bar, 100 μm. **c** IHC shows overlap between a tumor specific EC marker (CD105, green) and a general EC marker (CD31, red). Scale bar, 100 μm. **d** Example of FACS sort for CD105+ tumor ECs from a freshly resected GBM (left). Purity of the sorted fraction was confirmed using a fluorescent microscope (right). Scale bar, 50 μm. **e** Isolated tumor ECs were incubated with PU-H71 at the indicated concentrations (n = 3 technical repeats). After 6 days, cell viability was assessed by an ATP-based assay. Data are presented as mean ± s.e.m.. Source data are provided as a Source Data file.

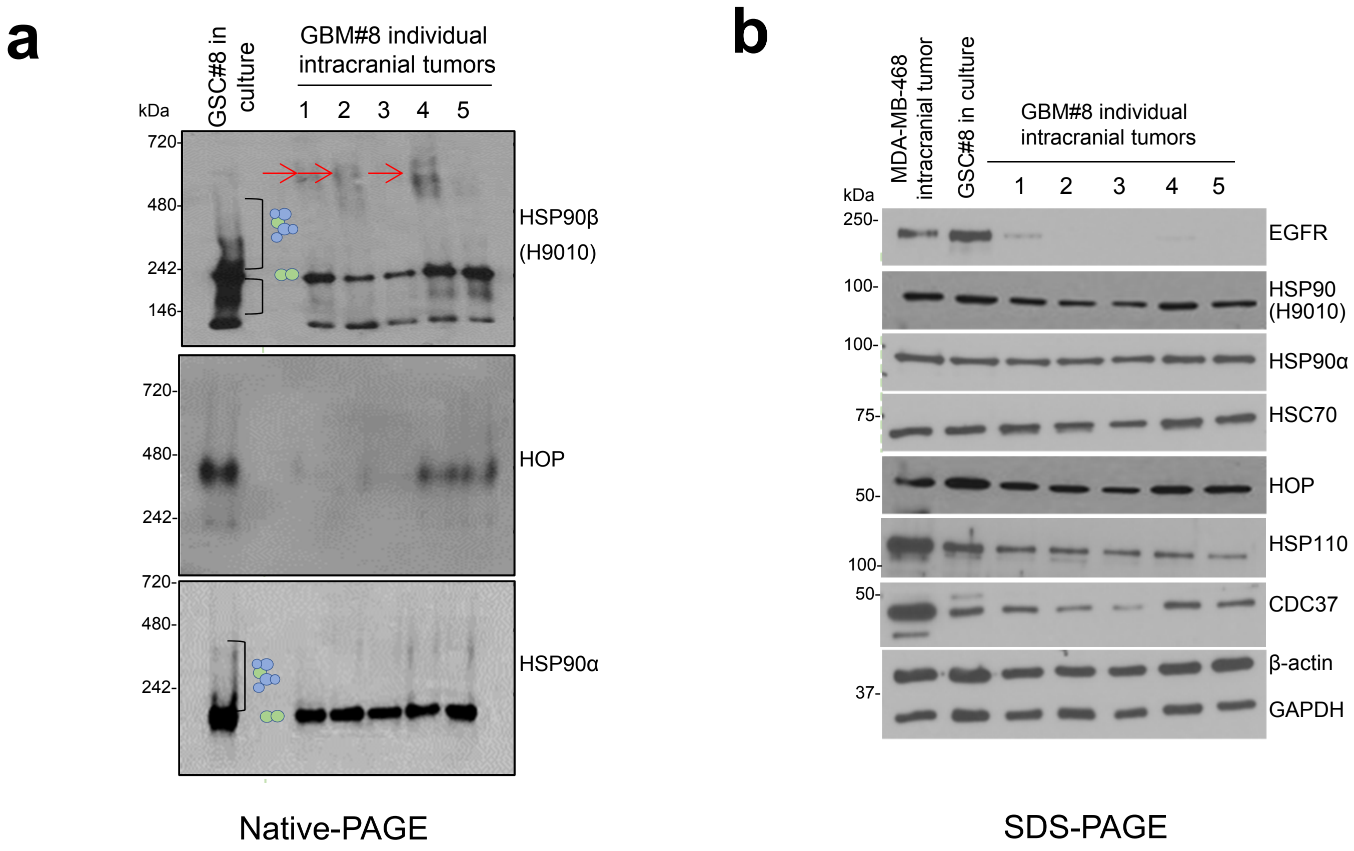

Supplementary Fig. 20: Epichaperome analysis in GBM#8. a,**b** Analysis of individual GBM#8 PDX tumors (n = 5) and of GSC#8 cells in culture. Native- (**a**) and SDS-PAGE (**b**) analyses are shown. When HSP90 is incorporated into epichaperomes, soluble oligomers of enhanced stability, these complexes withstand separation and are detected upon immunoblotting (see their position denoted with black brackets). Conversely, dynamic HSP90 complexes involved in folding dissociate and HSP90 is seen putatively as a dimer (see band at ~242 kDa). Red arrow, mouse IgG, unspecific signal. Source data are provided as a Source Data file.

Supplementary Note 1

Synthetic Procedures

General: All commercial chemicals and solvents were reagent grade and used without further purification. The identity and purity of each product was characterized by MS, HPLC, TLC, and NMR. $^{1}H/^{13}C$ NMR spectra were recorded on either a Bruker 400, 500 or 600 MHz instrument. Chemical shifts are reported in δ values in ppm downfield from TMS as the internal standard. ^{1}H data are reported as follows: chemical shift, multiplicity (s = singlet, d = doublet, t = triplet, q = quartet, br = broad, m = multiplet), coupling constant (Hz), integration. ^{13}C chemical shifts are reported in δ values in ppm downfield from TMS as the internal standard. High resolution mass spectra were recorded on a Waters LCT Premier system. Low resolution mass spectra were obtained on Waters Acquity Ultra Performance LC with electrospray ionization and SQ detector. HPLC analysis was done on Waters Autopurification system with PDA, MicroMass ZQ and ELSD detector and a reversed phase column (Waters X-Bridge C18, 4.6 x 150 mm, 5 µm) eluted with water/acetonitrile gradients, containing 0.1% TFA. Purity of target compounds has been determined to be >95% by HPLC. Column chromatography was performed using 230-400 mesh silica gel. Analytical thin layer chromatography was performed on 250 µM silica gel F_{254} plates. Preparative thin layer chromatography was performed on 1000 µM silica gel F_{254} plates. All reactions were performed under argon protection.

8-(6-Iodo-benzo[1,3]dioxol-5-ylsulfanyl)-9-(3-isopropylaminopropyl)adenine (1) (PU-H71). 1 was prepared as previously published[1]. ^{1}H NMR (400 MHz, $CDCl_3$) δ 8.31 (s, 1H), 7.29 (s, 1H), 6.88 (s, 1H), 6.10 (bs, 2H), 5.96 (s, 2H), 4.29 (t, *J* = 7.0 Hz, 2H), 2.75-2.69 (m, 1H), 2.58 (t, *J* = 6.8 Hz, 2H), 2.02- 1.95 (m, 2H), 1.03 (d, *J* = 6.2 Hz, 6H); ^{13}C NMR (100 MHz, $CDCl_3$) δ 154.6, 152.9, 151.6, 149.2, 148.9, 146.2, 127.9, 120.1, 119.2, 112.2, 102.2, 91.1, 48.7, 43.9, 41.7, 30.3, 22.9; MS m/z 513.2 $[M + H]^+$.

8-(6-Bromo-benzo[1,3]dioxol-5-ylsulfanyl)-9-(3-isopropylaminopropyl)adenine (3) (PU-H64). 3 was prepared as previously published[1]. ^{1}H NMR (400 MHz, $CDCl_3$) δ 8.30 (s, 1H), 7.04 (s, 1H), 6.81(s, 1H), 6.48 (bs, 2H), 5.94 (s, 2H), 4.29 (t, *J* = 7.0 Hz, 2H), 2.74-2.68 (m, 1H), 2.57 (t, *J* = 6.8 Hz, 2H), 2.02- 1.95 (m, 2H), 1.02 (d, *J* = 6.0 Hz, 6H); ^{13}C NMR (100 MHz, $CDCl_3$) δ 154.8, 152.9, 151.5, 148.8, 148.0, 145.2, 123.8, 120.0, 116.7, 113.2, 112.2, 102.3, 48.6, 43.8, 41.7, 30.2, 22.8; MS m/z 465.0 $[M + H]^+$.

***tert*-Butyl 3-(6-amino-8-(6-iodobenzo[d][1,3]dioxol-5-ylthio)-9H-purin-9-yl)propyl(isopropyl)carbamate (4). 4** was prepared as previously published[2]. A solution of **1** (70 mg, 0.137 mmol), $(Boc)_2O$ (35 mg, 0.161 mmol) and triethylamine (13 mg, 0.137 mmol) in THF (2 mL) was stirred at room temperature for 12 h. Following solvent removal, the residue was purified by preparative thin layer chromatography ($CHCl_3$:MeOH-NH_3 (7N), 20:1) to afford **4** (74 mg, 88%); ^{1}H NMR (500 MHz, $CDCl_3$) δ 8.33 (s, 1H), 7.30 (s, 1H), 6.90 (s, 1H), 5.99 (s, 2H), 5.49 (br s, 2H), 4.23 (m, 2H), 4.12 (m, 1H), 3.10 (m, 2H), 2.04 (m, 2H), 1.41 (s, 9H), 1.07 (d, *J* = 6.2 Hz, 6H); MS m/z 613.3 $[M + H]^+$.

6-((6-Amino-9-(3-(isopropylamino)propyl)-9H-purin-8-yl)thio)benzo[d][1,3]dioxoles-5-carbonitrile (5) (PU-WS5). To a solution of **4** (26 mg, 0.04 mmol) in DMF (3 mL) was added $PdCl_2$(dppf) (17 mg, 0.02 mmol), $Zn(CN)_2$ (10 mg, 0.08 mmol) and Zn (3 mg, 0.04 mL) and the resulting mixture was stirred at 90 °C for 6h. The reaction mixture was condensed under reduced pressure and the residue was purified by flash chromatography ($CHCl_3$:MeOH-NH_3 (7N), 20:1) to yield Boc-protected **5** as a white solid. To a solution of this in CH_2Cl_2 (2 mL) was added 0.2 mL of TFA and the mixture was stirred at room temperature for 5 h. The reaction mixture was condensed under reduced pressure and the residue purified by flash chromatography ($CHCl_3$:MeOH-NH_3 (7N), 20:1) to yield **5** (12.2 mg, 70% for two steps) as a white solid. ^{1}H NMR (500 MHz, CD_3OD) δ 8.28 (s, 1H), 7.37 (s, 1H), 7.32 (s, 1H), 6.22 (s, 2H), 4.45 (m, 2H), 3.29 (m, 1H), 3.04 (t, 2H), 2.38 (m, 2H), 1.34 (d, 6H, *J* = 6.5 Hz); ^{13}C NMR (125 MHz, $CDCl_3$) δ 154.6, 153.0, 152.0, 151.6, 148.8, 145.0, 128.8, 120.1, 116.7, 114.2, 112.5, 109.5, 103.1, 48.7, 43.8, 41.8, 30.3, 22.8; MS (ESI) *m/z* 412.2 $[M+H]^+$.

1-(6-(6-Amino-9-(3-(isopropylamino)propyl)-9H-purin-8-ylthio)benzo[d][1,3]dioxol-5-yl)ethanone (6) (PU-DZ4-22-3). A mixture of **1** (30 mg, 0.0585 mmol), tri-*n*-butyl(1-ethoxy-vinyl)tin (84.5 mg, 79 µl, 0.234 mmol), LiCl (5 mg, 0.117 mmol) and $Pd(PPh_3)_4$ (6.7 mg, 0.0058 mmol) in DMF (1 mL) was evacuated and back filled with nitrogen. This was repeated four times then the reaction mixture was heated under nitrogen at 90 °C for 18 h. Solvent was removed under reduced pressure and the resulting residue was hydrolyzed by stirring in a mixture

of CH_2Cl_2/1M HCl for 10 minutes at rt. The organic layer was separated and washed with 10% Na_2CO_3, dried over $MgSO_4$ and concentrated. The resulting residue was purified by preparatory TLC two times (hexane:CH_2Cl_2:EtOAC:MeOH-NH_3 (7N), 2:2:1:0.5 and CH_2Cl_2:MeOH-NH_3 (7N), 10:1) to give 16.3 mg (65%) of **6**. ^{1}H NMR (500 MHz, $CDCl_3/CD_3OD$) δ 8.28 (s, 1H), 7.44 (s, 1H), 6.26 (s, 1H), 6.07 (s, 2H), 4.30 (t, *J* = 6.9 Hz, 2H), 2.94 (m, 1H), 2.68 (t, *J* = 7.0 Hz, 2H), 2.64 (s, 3H), 2.08 (m, 2H), 1.16 (d, *J* = 6.4 Hz, 6H); ^{13}C NMR (125 MHz, $CDCl_3$ + MeOH-d_4) δ 201.4, 159.1, 157.1, 155.9, 154.9, 150.9, 149.6, 135.1, 132.6, 123.6, 114.4, 112.1, 106.7, 53.9, 45.6, 44.6, 31.5, 31.1, 23.4; HRMS (ESI) *m/z* $[M+H]^+$ calcd. for $C_{20}H_{25}N_6O_3S$, 429.1709; found 429.1711.

8-(6-Vinylbenzo[d][1,3]dioxol-5-ylthio)-9-(3-(isopropylamino)propyl)-9*H*-purin-6-amine (7) (PU-HT65). 4 (26 mg, 0.042 mmol), tributylvinyltin (17 mg, 0.052 mmol) and $Pd(PPh_3)_4$ (5 mg, 0.004 mmol) in anhydrous DMF (1 mL) was stirred at 110 °C for 6 h. Following solvent removal, the residue was purified by preparative thin layer chromatography ($CHCl_3$:MeOH-NH_3 (7N), 20:1) to afford Boc-protected **7**, which was dissolved in 10% TFA-CH_2Cl_2 and stirred at room temperature for 2 h. Following solvent removal, the residue was purified by preparative thin layer chromatography ($CHCl_3$:MeOH-NH_3 (7N), 20:1) to yield **7** (6 mg, 36% for two steps). ^{1}H NMR ($CDCl_3$, 500 MHz) δ 8.29 (s, 1H), 7.23 (dd, *J* = 17.3, 10.9 Hz, 1H), 7.10 (s, 1H), 6.92 (s, 1H), 5.99 (s, 2H), 5.64 (br s, 2H), 5.61 (d, *J* = 17.3 Hz, 1H), 5.29 (d, *J* = 11.0 Hz, 1H), 4.26 (t, *J* = 7.1 Hz, 2H), 2.73 (septet, *J* = 6.3 Hz, 1H), 2.57 (t, *J* = 6.8 Hz, 2H), 1.97 (m, 2H), 1.05 (d, *J* = 6.3Hz, 6H); ^{13}C NMR ($CDCl_3$, 125 MHz) δ 154.1, 152.6, 148.0, 133.7, 115.7, 113.6, 106.0, 101.9, 48.8, 47.2, 43.8, 41.5, 30.0, 29.7, 22.7, 19.2; MS (ESI) *m/z* 412.9 $[M+H]^+$.

8-(6-Allylbenzo[d][1,3]dioxol-5-ylthio)-9-(3-(isopropylamino)propyl)-9*H*-purin-6-amine (8) (PU-HT64). A solution of **4** (26 mg, 0.042 mmol), tributylallyltin (17 mg, 0.052 mmol) and $Pd(PPh_3)_4$ (5 mg, 0.004 mmol) in anhydrous DMF (1 mL) was stirred at 110 °C for 6 h. Following solvent removal, the residue was purified by preparative thin layer chromatography ($CHCl_3$:MeOH-NH_3 (7N), 20:1) to afford Boc-protected **8** (7.3 mg, 33%; MS *m/z* 527.1 $[M+H]^+$). A solution of Boc-protected **8** (7.3 mg, 0.014 mmol) in 10% TFA-CH_2Cl_2 was stirred at room temperature for 2h. Following solvent removal, the residue was purified by preparative thin layer chromatography ($CHCl_3$:MeOH-NH_3 (7N), 20:1) to yield **7** (4.6 mg, 77%). ^{1}H NMR ($CDCl_3$, 500 MHz) δ 8.27 (s, 1H), 6.92 (s, 1H), 6.80 (s, 1H), 5.99 (s, 2H), 5.84-5.91 (m, 1H), 5.59 (br s, 2H), 4.98-5.07 (m, 2H), 4.28 (t, *J* = 6.9 Hz, 2H), 3.54 (m, 2H), 2.77 (septet, *J* = 6.3 Hz, 1H), 2.58 (t, *J* = 6.8 Hz, 2H), 1.94-2.04 (m, 2H), 1.07 (d, *J* = 6.3 Hz, 6H); ^{13}C NMR ($CDCl_3$, 125 MHz) δ 154.0, 152.4, 151.9, 147.8, 146.9, 136.9, 136.2, 119.5, 116.6, 113.9, 110.3, 101.7, 48.9, 43.5, 41.2, 38.3, 29.7, 22.5; MS (ESI) *m/z* 426.8 $[M+H]^+$.

9-(3-(Isopropylamino)propyl)-8-(6-phenylbenzo[d][1,3]dioxol-5-ylthio)-9H-purin-6-amine (9) (PU-DZ2-388). 9 was prepared as previously published[3]. ^{1}H NMR (500 MHz, MeOH-d_4) δ 8.14 (s, 1H), 7.28-7.34 (m, 3H), 7.17-7.21 (m, 2H), 7.12 (s, 1H), 6.90 (s, 1H), 6.09 (s, 2H), 4.03 (t, *J* = 6.4 Hz, 2H), 3.27 (septet, *J* = 6.6 Hz, 1H), 2.72 (t, *J* = 6.6 Hz, 2H), 2.13 (m, 2H), 1.40 (d, *J* = 6.5 Hz, 6H); ^{13}C NMR (125 MHz, CD_3OD) δ 156.0, 153.4, 152.1, 151.1, 150.3, 149.4, 142.3, 141.8, 130.4, 129.1, 128.7, 120.3, 119.8, 115.7, 112.2, 103.8, 52.2, 43.2, 41.1, 27.6, 19.3; HRMS (ESI) *m/z* $[M+H]^+$ calcd. for $C_{24}H_{27}N_6O_2S$, 463.1916; found 463.1905.

8-(6-(4-Bromophenyl)benzo[d][1,3]dioxol-5-ylthio)-9-(3-(isopropylamino)propyl)-9H-purin-6-amine (10) (PU-DZ3-6). 10 was prepared as previously published[3]. ^{1}H NMR (500 MHz, $CDCl_3$) δ 8.23 (s, 1H), 7.44 (d, *J* = 8.2 Hz, 2H), 7.13 (d, *J* = 8.2 Hz, 2H), 7.01 (s, 1H), 6.81 (s, 1H), 6.05 (s, 2H), 5.69 (br s, 2H), 4.08 (t, *J* = 6.0 Hz, 2H), 2.91 (m, 1H), 2.50 (t, *J* = 5.9 Hz, 2H), 1.98 (m, 2H), 1.20 (d, *J* = 6.4 Hz, 6H); ^{13}C NMR (125 MHz, $CDCl_3$/ CD_3OD) δ 154.2, 152.0, 151.2, 149.8, 149.1, 148.2, 139.8, 139.1, 131.2, 131.0, 122.0, 119.0, 117.9, 115.0, 111.1, 102.4, 50.1, 42.1, 40.2, 27.3, 20.2; HRMS (ESI) *m/z* $[M+H]^+$ calcd. for $C_{24}H_{26}BrN_6O_2S$, 541.1021/ 543.1001; found 541.1016/543.1004.

8-(6-(4-*tert*-Butylphenyl)benzo[d][1,3]dioxol-5-ylthio)-9-(3-(isopropylamino)propyl)-9H-purin-6-amine (11) (PU-DZ2-390). 11 was prepared as previously published[3]. ^{1}H NMR (500 MHz, MeOH-d_4) δ 8.11 (s, 1H), 7.27 (d, *J* = 8.4 Hz, 2H), 7.14 (s, 1H), 7.12 (d, *J* = 8.4 Hz, 2H), 6.86 (s, 1H), 6.06 (s, 2H), 3.93 (t, *J* = 6.9 Hz, 2H), 2.92 (septet, *J* = 6.5 Hz, 1H), 2.61 (t, *J* = 7.3 Hz, 2H), 1.86 (m, 2H), 1.28 (s, 9H), 1.12 (d, *J* = 6.5 Hz, 6H); ^{13}C NMR (125 MHz, CD_3OD) δ 155.9, 153.3, 151.9, 151.8, 150.9, 150.2, 149.2, 141.9, 138.8, 130.0, 125.9, 120.4, 120.3, 115.4, 112.3, 103.6, 50.6, 44.0, 41.8, 35.4, 31.8, 29.3, 21.1; HRMS (ESI) *m/z* $[M+H]^+$ calcd. for $C_{28}H_{35}N_6O_2S$, 519.2542; found 519.2545.

8-(6-(4-(Dimethylamino)phenyl)benzo[d][1,3]dioxol-5-ylthio)-9-(3-(isopropylamino)propyl)-9H-purin-6-amine (12) (PU-DZ2-392). **12** was prepared as previously published[3]. ^{1}H NMR (500 MHz, $CDCl_3$) δ 8.24 (s, 1H), 7.13 (d, *J* = 8.7 Hz, 2H), 6.93 (s, 1H), 6.83 (s, 1H), 6.67 (d, *J* = 8.7 Hz, 2H), 6.01 (br s, 2H), 5.98 (s, 2H), 4.02 (t, *J* = 6.7 Hz, 2H), 2.97 (s, 6H), 2.78 (septet, *J* = 6.3 Hz, 1H), 2.44 (t, *J* = 6.7 Hz, 2H), 1.87 (m, 2H), 1.10 (d, *J* = 6.3 Hz, 6H); ^{13}C NMR (125 MHz, $CDCl_3$) δ 154.4, 152.4, 151.5, 149.9, 148.5, 148.0, 147.1, 139.6, 130.0, 127.8, 120.5, 119.7, 112.8, 111.7, 111.0, 101.7, 49.3, 43.1, 40.9, 40.4, 28.9, 21.9; HRMS (ESI) *m/z* $[M+H]^+$ calcd. for $C_{26}H_{32}N_7O_2S$, 506.2338; found 506.2330.

9-(3-Bromopropyl)-2-fluoro-8-((6-iodobenzo[d][1,3]dioxol-5-yl)methyl)-9H-purin-6-amine (15). **15** was prepared from **13** as previously published[1]. ^{1}H NMR ($CDCl_3$/CD_3OD, 400 MHz) δ 7.33 (s, 1H), 6.71 (s, 1H), 6.01 (s, 2H), 4.28 (s, 2H), 4.24 (t, *J* = 7.4 Hz, 2H), 3.43 (t, *J* = 6.4 Hz, 2H), 2.30-2.40 (m, 2H); MS (ESI): *m/z* 534.1/536.1 $[M + H]^+$.

8-((6-Bromobenzo[d][1,3]dioxol-5-yl)methyl)-9-(3-bromopropyl)-2-fluoro-9H-purin-6-amine (16). A solution of **14**[1] (0.57 g. 1.56 mmol), Cs_2CO_3 (0.55 g, 2.3 mmol) and 1,3-dibromopropane (1.57 g, 7.8 mmol) in anhydrous DMF (18 mL) was stirred at rt for 1 h. Then additional Cs_2CO_3 (0.18 g, 0.78 mmol) was added and the reaction mixture was stirred for an additional 1 h. Solids were filtered and the filtrate was concentrated under reduced pressure. The residue was purified by chromatography ($CHCl_3$/MeOH/AcOH, 220:1:0.5 to 180:1:0.5) to give **16** (0.65 g, 86%). ^{1}H NMR ($CDCl_3$/CD_3OD, 400 MHz) δ 7.07 (s, 1H), 6.61 (s, 1H), 5.96 (s, 2H), 4.29 (s, 2H), 4.16 (t, *J* = 7.4 Hz, 2H), 3.35 (t, *J* = 6.5 Hz, 2H), 2.29 (m, 2H); MS (ESI) *m/z* 485.9/487.9/489.9 $[M+H]^+$.

2-Fluoro-8-((7-iodobenzo[d][1,3]dioxol-5-yl)methyl)9-(3-isopropylamino)propyl)-9H-6-amine (17) (PU-DZ8). **17** was prepared as previously published[1]. ^{1}H NMR (400 MHz, $CDCl_3$) δ 7.29 (s, 1H), 6.59 (s, 1H), 5.94 (s, 2H), 5.89 (bs, 2H), 4.25 (s, 2H), 4.11 (t, *J* = 7.0 Hz, 2H), 2.73-2.60 (m, 1H), 2.55 (t, *J* = 6.8 Hz, 1H), 1.93-1.86 (m, 2H), 1.03-1.02 (d, *J* = 6.0 Hz, 6H); ^{13}C NMR (100 MHz, Methanol-d_4) δ 160.0, 158.4, 157.2, 152.4, 151.3, 149.4, 148.4, 133.1, 118.7, 110.6, 102.4, 88.5, 42.8, 40.1, 38.8, 27.6, 19.4; MS m/z 513.2 $[M + H]^+$.

8-((7-Bromobenzo[d][1,3]dioxol-5-yl)methyl)-2-fluoro-9-(3-(isopropylamino)propyl)-9H-purin-6-amine (18) (PU-DZ10). **16** (0.100 g, 0.205 mmol) was reacted with isopropylamine (1.7 mL, 1.2 g, 20.5 mmol) in DMF (3 mL) at rt and following purification **18** (67 mg, 70%) was obtained as a pale-yellow solid. ^{1}H NMR (400 MHz, CD_3OD) δ 7.09 (s, 1H), 6.79 (s, 1H), 6.01 (s, 2H), 4.28 (s, 2H), 4.19 (t, *J* = 7.2 Hz, 2H), 2.74 (septet, *J* = 6.4 Hz, 1H), 2.57 (t, *J* = 7 Hz, 2H), 1.97 (m, 2H), 1.07 (d, *J* = 6.4 Hz, 6H); ^{13}C NMR (100 MHz, $CDCl_3$/CD_3OD) δ 158.3 (d, *J* = 208 Hz), 156.1 (d, *J* = 20 Hz), 151.7 (d, *J* = 19 Hz), 150.3, 147.7, 147.5, 127.2, 115.3, 114.2, 112.2, 110.0, 101.7, 48.0, 42.8, 40.2, 33.5, 28.9, 21.2; MS (ESI) *m/z* 465.1/467.1 $[M+H]^+$.

6-((6-Amino-2-fluoro-9-(3-(isopropylamino)propyl)-9H-purin-8-yl)methyl)benzo[d][1,3]dioxole-5-carbonitrile (20) (PU-WS3). A solution of **17** (118 mg, 0.231 mmol), $(Boc)_2O$ (55 mg, 0.254 mmol) and triethylamine (16 mg, 0.231 mmol) in THF (2 mL) was stirred at room temperature for 12 h. Following solvent removal, the residue was purified by chromatography ($CHCl_3$:MeOH-NH_3 (7N), 20:1) to afford **19** in 85% yield (MS *m/z* 613.1 $[M+H]^+$). To a solution of **19** (26 mg, 0.04 mmol) in DMF (3 mL) was added $PdCl_2$(dppf) (17 mg, 0.02 mmol), $Zn(CN)_2$ (10 mg, 0.08 mmol) and Zn (3 mg, 0.04 mL) and the resulting mixture was stirred at 130 ^oC overnight. The reaction mixture was condensed under reduced pressure and the residue was purified by flash chromatography ($CHCl_3$:MeOH-NH_3 (7N), 20:1) to yield Boc-protected **20** as a white solid. To a solution of this in CH_2Cl_2 (2 mL) was added 0.2 mL of TFA and the mixture was stirred at room temperature for 5 h. The reaction mixture was condensed under reduced pressure and the residue purified by flash chromatography ($CHCl_3$:MeOH-NH_3 (7N), 20:1) to yield **20** (12 mg, 69% for two steps) as a white solid. ^{1}H NMR (500 MHz, CD_3OD) δ 7.13 (s, 1H), 6.96 (s, 1H), 6.03 (s, 2H), 4.31 (s, 2H), 4.25 (t, *J* = 7 Hz, 2H), 3.26 (m, 1H), 3.01 (t, *J* = 7.5 Hz, 2H), 2.14 (m, 2H), 1.23 (d, *J* = 6.5 Hz, 6H); ^{13}C NMR (125 MHz, CD_3OD) δ 160.4 (d, *J* = 208 Hz), 158.5 (d, *J* = 19 Hz), 153.8, 153.5 (d, *J* = 19 Hz), 151.7, 149.1, 137.2, 119.1, 117.4, 112.6, 112.4, 106.3, 104.4, 52.3, 43.4, 40.8, 33.2, 27.7, 19.3; MS (ESI) *m/z* 412.3 $[M+H]^+$.

8-((6-Ethylbenzo[d][1,3]dioxol-5-yl)methyl)-2-fluoro-9-(3-(isopropylamino)propyl)-9H-purin-6-amine (21) (PU-HT133). Boc-protected **22** (22 mg, 0.043 mmol), $NaBH_4$ (10 mg, 0.263 mmol) and 10% Pd/C (10 mg) in anhydrous THF (2 mL) was stirred at room temperature for 48 h. The reaction mixture was quenched with 2N HCl to pH ~ 2. After the pH was adjusted to neutral by saturated K_2CO_3, the reaction mixture was extracted with EtOAc (3 x 5 mL). The combined organic layer was dried over $MgSO_4$ and condensed under vacuum. The

resulting residue was dissolved in 10% TFA-CH_2Cl_2 (0.5 mL) and stirred at room temperature for 2 h. Following solvent removal, **21** (6.2 mg, 35%) was obtained after preparative thin layer chromatography ($CHCl_3$:MeOH-NH_3 (7N), 20:1). ^1H NMR ($CDCl_3$, 500 MHz) δ 6.73 (s, 1H), 6.43 (s, 1H), 5.90 (s, 2H), 5.72 (br s, 2H), 4.18 (s, 2H), 4.06 (t, J = 7.0 Hz, 2H), 2.60-2.71 (m, 3H), 2.49 (t, J = 6.6 Hz, 2H), 1.84 (m, 2H), 1.18 (t, J = 7.6 Hz, 3H), 1.01 (d, J = 6.2 Hz, 6H); ^{13}C NMR ($CDCl_3$, 125 MHz) δ 157.8 (d, J = 208 Hz), 155.9 (d, J = 22 Hz), 153.0 (d, J = 17 Hz), 151.7, 146.9, 145.9, 135.6, 126.0, 116.8, 109.0, 108.9, 101.0, 48.8, 43.7, 41.0, 31.2, 30.2, 25.7, 22.9, 15.0; MS (ESI) *m/z* 415.0 $[M+H]^+$.

2-Fluoro-8-((6-vinylbenzo[d][1,3]dioxol-5-yl)methyl)--9-(3-(isopropylamino)propyl)-9*H*-purin-6-amine (22) (PU-HT70). A solution of **19** (26 mg, 0.042 mmol), tributylvinyltin (17 mg, 0.052 mmol) and $Pd(PPh_3)_4$ (5 mg, 0.004 mmol) in anhydrous DMF (1 mL) was stirred at 110 °C for 6 h. Following solvent removal, the residue was purified by preparative thin layer chromatography ($CHCl_3$:MeOH-NH_3 (7N), 20:1) to afford Boc-protected **22** (MS *m/z* 513.1 $[M+H]^+$), which was dissolved in 10% TFA-CH_2Cl_2 and stirred at room temperature for 2 h. Following solvent removal, the residue was purified by preparative thin layer chromatography ($CHCl_3$:MeOH-NH_3 (7N), 20:1) to yield **22** (6.4 mg, 32% for three steps). ^1H NMR ($CDCl_3$, 500 MHz) δ 7.00 (s, 1H), 6.99 (dd, J = 17.2, 10.8 Hz, 1H), 6.52 (s, 1H), 5.93 (s, 2H), 5.87 (br s, 2H), 5.55 (dd, J = 17.0, 0.8 Hz, 1H), 5.28 (dd, J = 11.0, 1.0 Hz, 1H), 4.23 (s, 2H), 4.04 (t, J = 7.0 Hz, 2H), 2.74 (septet, J = 6.3 Hz, 1H), 2.50 (t, J = 6.7 Hz, 2H), 1.83 (m, 2H), 1.06 (d, J = 6.3 Hz, 6H); ^{13}C NMR ($CDCl_3$, 125 MHz) δ 158.3 (d, J = 208 Hz), 156.1 (d, J = 20 Hz), 153.0 (d, J = 19 Hz), 151.5, 147.8, 147.3, 133.6, 130.8, 126.5, 118.7, 115.8, 109.0, 106.2, 101.3, 49.0, 43.5, 40.8, 31.3, 29.8, 22.6; MS (ESI) *m/z* 413.2 $[M+H]^+$.

2-Fluoro-8-((6-allylbenzo[d][1,3]dioxol-5-yl)methyl)-9-(3-(isopropylamino)propyl)-9*H*-purin-6-amine (23) (PU-HT78). A solution of **19** (26 mg, 0.042 mmol), tributylallyltin (17 mg, 0.052 mmol) and $Pd(PPh_3)_4$ (5 mg, 0.004 mmol) in anhydrous DMF (1 mL) was stirred at 110 °C for 6 h. Following solvent removal, the residue was purified by preparative thin layer chromatography ($CHCl_3$:MeOH-NH_3 (7N), 20:1) to afford Boc-protected **23** (7.7 mg, 35%; MS *m/z* 527.1 $[M+H]^+$), which was dissolved in 10% TFA-CH_2Cl_2 and stirred at room temperature for 2 h. Following solvent removal, the residue was purified by preparative thin layer chromatography ($CHCl_3$:MeOH-NH_3 (7N), 20:1) to yield **23** (3.2 mg, 51%). ^1H NMR ($CDCl_3$, 500 MHz) δ 6.71 (s, 1H), 6.41 (s, 1H), 5.93 (s, 2H), 5.82-5.88 (m, 1H), 5.06 (d, J = 10 Hz, 1H), 4.95 (d, J = 17.5 Hz, 1H), 4.18-4.20 (m, 2H), 4.13 (s, 2H), 3.46-3.49 (m, 2H), 3.25-3.29 (m, 1H), 2.74 (t, J = 6 Hz, 2H), 1.97-2.05 (m, 2H), 1.41 (d, J = 6.2 Hz, 6H); ^{13}C NMR (150 MHz, $CDCl_3$) δ 158.60 (d, J = 208.5 Hz), 156.08 (d, J = 19.5 Hz), 152.96 (d, J = 18 Hz), 151.54 (d, J = 3 Hz), 146.96, 146.52, 136.29, 131.17, 126.60, 116.15, 110.45, 108.99, 101.17, 49.33, 43.12, 40.60, 37.38, 31.22, 29.73, 22.13. MS (ESI) *m/z* 426.9 $[M+H]^+$.

6-Iodo-2,3-dihydrobenzo[b][1,4]dioxine (25). 2,3-Dihydrobenzo[b][1,4]dioxin-6-amine (**24**; 5 g, 33 mmol) was dissolved in 10% HCl solution and cooled to 0 °C. Then, 30 mL of a cold aqueous solution of $NaNO_2$ (4.6 g, 66 mmol) was added over a period of 15 min and the reaction mixture was stirred at 0 °C for an additional 10 min, followed by the addition of urea (1.6 g, 27 mmol). After 15 min, 40 mL of a suspension of KI (16.5 g, 100 mmol) in water/CH_2Cl_2 (1:1) was added. The reaction mixture was stirred overnight at room temperature then extracted with CH_2Cl_2, dried over $MgSO_4$ and condensed under reduced pressure and the residue was purified by flash chromatography (hexane:EtOAc, 100:0 to 90:10) to afford **25** (7.4 g, 86%) as a colorless oil. ^1H NMR (500 MHz, $CDCl_3$) δ 7.28 δ (s, 1H), 7.13 (d, J = 8.5 Hz, 1H), 6.63 (d, J = 8.5 Hz, 1H), 4.27-4.24 (m, 4H).

8-(7-Iodo-2,3-dihydrobenzo[b][1,4]dioxin-6-ylthio)-9*H*-purin-6-amine (26). To a solution of **25** (1.26 g, 4.8 mmol) in DMF (15 mL) was added 8-mercaptoadenine (0.400 g, 2.4 mmol), neocuproine (0.056 g, 0.24 mmol), CuI (0.044 g, 0.24 mmol) and Na*t*OBu (0.460 g, 4.8 mmol). The reaction mixture was stirred at 110 °C for 24 h. Solids were filtered and the filtrate was condensed under reduced pressure. The residue was flash chromatographed ($CHCl_3$:MeOH:AcOH, 60:0.5:0.5 to 30:0.5:0.5) to yield 0.578 g (80%) of intermediate coupling product (MS (ESI) *m/z* 301.9 $[M+H]^+$). To 0.400 g (1.4 mmol) of this and NIS (0.945 g, 4.2 mmol) in acetonitrile (15 mL) was added TFA (540 μL, 0.800 g, 7 mmol) and the mixture was stirred at room temperature overnight. The solvent was removed under reduced pressure and the residue was purified by flash chromatography ($CHCl_3$:MeOH:AcOH, 60:0.5:0.5 to 30:0.5:0.5) to give **26** (0.436 g, 73%). ^1H NMR (DMSO-d_6, 500 MHz) δ 8.37 (s, 1H), 8.03 (br s, 2H), 7.47 (s, 1H), 7.10 (s, 1H), 4.25-4.27 (m, 4H); MS (ESI) *m/z* 427.9 $[M+H]^+$.

9-(3-Bromopropyl)-8-(7-iodo-2,3-dihydrobenzo[b][1,4]dioxin-6-ylthio)-9*H*-purin-6-amine (27). A mixture of **26** (0.213 g, 0.5 mmol), 1,3-dibromopropane (0.512 g, 2.5 mmol) and Cs_2CO_3 (0.184 g, 0.75 mmol) in anhydrous

DMF (6 mL) was stirred at room temperature for 3 h. Solids were filtered and the filtrate was condensed under reduced pressure to give a residue that was purified by preparative thin layer chromatography ($CHCl_3$:MeOH-NH_3 (7N), 20:1) to give **27** (0.104 g, 38%). ^{1}H NMR ($CDCl_3$, 500 MHz) δ 8.26 (s, 1H), 7.32 (s, 1H), 6.94 (s, 1H), 5.6 (br s, 2H), 4.27 (t, *J* = 7.0 Hz, 2H), 4.10-4.17 (m, 4H), 3.32 (t, *J* = 7.0 Hz, 2H), 2.26 (m, 2H); MS (ESI) *m/z* 547.9/549.8 $[M+H]^+$.

8-(7-Iodo-2,3-dihydrobenzo[b][1,4]dioxin-6-ylthio)-9-(3-(isopropylamino)propyl)-9H-purin-6-amine (28) (PU-RK11). **27** (0.045 g, 0.082 mmol) and isopropylamine (0.242 g, 4.1 mmol) in DMF (1 mL) was stirred overnight at rt. Solvent was removed under reduced pressure and the resulting residue was purified by chromatography (CH_2Cl_2:MeOH) to give 0.038 g (88%) of **28**. ^{1}H NMR (500 MHz, $CDCl_3$) δ 8.30 (s, 1H), 7.38 (s, 1H), 6.95 (s, 1H), 5.65 (br s, 2H), 4.32 (t, *J* = 6.9 Hz, 2H), 4.22-4.24 (m, 4H), 2.80 (septet, *J* = 6.7 Hz, 1H), 2.61 (t, *J* = 6.7 Hz, 2H), 2.07 (m, 2H), 1.11 (d, *J* = 6.7 Hz, 6H); ^{13}C NMR (125 MHz, $CDCl_3$) δ 154.4, 152.8, 151.7, 146.7, 144.8, 144.6, 128.3, 127.8, 121.3, 120.1, 91.0, 64.3, 64.2, 49.0, 43.6, 41.6, 29.8, 22.5; MS (ESI) *m/z* 527.1 $[M+H]^+$.

3,4-Dihydro-2H-benzo[b][1,4]dioxepine (30). A solution of catechol (**29**; 3.0 g, 0.0272 mol) in DMF (30 mL) was stirred for several minutes, then K_2CO_3 (9.40 g, 0.068 mol) and 1,3-dibromopropane (6.04 g, 3.06 mL, 0.02992 mol) were added and the mixture was stirred at rt for 3 h. The reaction mixture was added to dH_2O (300 mL) and extracted with EtOAc (3 x 70 mL). The combined organic layers were washed with 1M NaOH (3 x 100 mL) and dH_2O (100 mL), dried over Mg_2SO_4, filtered and concentrated under reduced pressure. The residue was purified by chromatography (hexane:EtOAc, 10:1 to 40:1) to give 1.80 g (44%) of **30** as a clear oil. ^{1}H NMR (500 MHz, $CDCl_3$) δ 6.95-6.99 (m, 2H), 6.90-6.94 (m, 2H), 4.22 (t, *J* = 5.6 Hz, 4H), 2.19 (pentet, *J* = 5.6 Hz, 2H); GCMS *m/z* 150.0.

7-Iodo-3,4-dihydro-2H-benzo[b][1,4]dioxepine (31). To a mixture of **30** (1.40 g, 9.32 mmol) and oxone (6.30 g, 10.25 mmol) in MeOH (45 mL) was added ammonium iodide (1.49 g, 10.25 mmol) and was stirred for 24 h at rt. The reaction mixture was filtered and MeOH removed under reduced pressure. The residue was taken up into CH_2Cl_2 (150 mL), washed with brine (3 x 50 mL), dried over $MgSO_4$ and concentrated under reduced pressure. The residue was purified by chromatography (hexane:EtOAc, 95:5) to give 1.80 g (70%) of **31** as a clear oil. ^{1}H NMR (500 MHz, $CDCl_3$) δ 7.31 (d, *J* = 2.1 Hz, 1H), 7.21 (dd, *J* = 2.1, 8.4 Hz, 1H), 6.70 (d, *J* = 8.4 Hz, 1H), 4.18-4.23 (m, 4H), 2.16-2.22 (m, 2H); GCMS *m/z* 275.9.

8-(3,4-Dihydro-2H-benzo[b][1,4]dioxepin-7-ylthio)-9H-purin-6-amine (32). 8-Mercaptoadenine (0.727 g, 4.35 mmol), neocuproine hydrate (0.148 g, 0.653 mmol), CuI (0.124 g, 0.653 mmol), K_2CO_3 (1.80 g, 13.1 mmol), **31** (1.8 g, 6.52 mol), and DMF (60 mL) were heated at 120 ˚C for 24 h. The solvent was removed under reduced pressure and the residue was purified by chromatography (CH_2Cl_2:MeOH:AcOH, 100:0:0 to 20:1:0.5) to give 0.77 g (56%) of **32** as a solid. ^{1}H NMR (500 MHz, $CDCl_3$/CD_3OD) δ 8.14 (s, 1H), 7.22 (d, *J* = 1.7 Hz, 1H), 7.16 (dd, *J* = 1.8, 8.2 Hz, 1H), 7.01 (d, *J* = 8.3 Hz, 1H), 4.22-4.31 (m, 4H), 2.19-2.27 (m, 2H); MS (ESI) *m/z* 316.0 $[M+H]^+$.

8-(8-Iodo-3,4-dihydro-2H-benzo[b][1,4]dioxepin-7-ylthio)-9H-purin-6-amine (33). **32** (0.078 g, 0.247 mmol), NIS (0.277 g, 1.23 mmol), CH_3CN (3.5 mL), TFA (0.564 g, 0.380 mL, 4.94 mmol) was stirred at rt overnight. The mixture was dried under reduced pressure and the residue purified by preparatory chromatography (CH_2Cl_2:MeOH:AcOH, 20:1:0.5) to give 0.076 g (70%) of **33**. ^{1}H NMR (500 MHz, $CDCl_3$/CD_3OD) δ 7.57 (s, 1H), 4.29 (t, *J* = 5.8 Hz, 2H), 4.26 (t, *J* = 5.8 Hz, 2H), 2.21-2.27 (m, 2H); MS (ESI) *m/z* 441.8 $[M+H]^+$.

9-(3-Bromopropyl)-8-(8-iodo-3,4-dihydro-2H-benzo[b][1,4]dioxepin-7-ylthio)-9H-purin-6-amine (34). **33** (0.060 g, 0.136 mmol), Cs_2CO_3 (0.443 g, 1.36 mmol), 1,3-dibromopropane (0.274 g, 0.139 mL, 1.36 mmol) in DMF (2 mL) was sonicated for 3 h. The mixture was dried under reduced pressure and the residue purified by preparatory chromatography (CH_2Cl_2:MeOH:AcOH, 25:1:0.5) to give 0.015 g (20%) of **34**. ^{1}H NMR (500 MHz, $CDCl_3$) δ 8.36 (s, 1H), 7.48 (s, 1H), 6.94 (s, 1H), 6.51 (br s, 2H), 4.35 (t, *J* = 7.1 Hz, 2H), 4.23 (t, *J* = 5.7 Hz, 2H), 4.20 (t, *J* = 5.7 Hz, 2H), 3.38 (t, *J* = 6.6 Hz, 2H), 2.34 (m, 2H), 2.19 (m, 2H); MS (ESI) *m/z* 561.7/563.9 $[M+H]^+$.

8-(8-Iodo-3,4-dihydro-2H-benzo[b][1,4]dioxepin-7-ylthio)-9-(3-(isopropylamino)propyl)-9H-purin-6-amine (35) (PU-DZ3-117). **34** (10 mg, 0.0177 mmol) and isopropylamine (10.5 mg, 150 µl, 1.77 mmol) in DMF (1 mL) was stirred overnight at rt. Solvent was removed under reduced pressure and the resulting residue was purified

by preparatory chromatography (CH_2Cl_2:hexane:EtOAc:MeOH-NH_3 (7N), 2:2:1:0.5) to give 3.5 mg (37%) of **35**. ^1H NMR (500 MHz, $CDCl_3$) δ 8.28 (s, 1H), 7.48 (s, 1H), 6.97 (s, 1H), 5.73 (br s, 2H), 4.39 (t, *J* = 6.4 Hz, 2H), 4.23 (t, *J* = 5.7 Hz, 2H), 4.20 (t, *J* = 5.7 Hz, 2H), 2.99-3.08 (m, 1H), 2.69 (t, *J* = 6.4 Hz, 2H), 2.24 (m, 2H), 2.15-2.22 (m, 2H), 1.27 (d, *J* = 6.3 Hz, 6H); ^{13}C NMR (150 MHz, $CDCl_3$) δ 154.6, 153.1, 151.9, 151.7, 151.6, 145.9, 132.5, 129.9, 124.5, 120.2, 92.1, 70.6, 70.6, 48.8, 43.7, 41.7, 31.2, 30.1, 22.8; HRMS (ESI) *m/z* $[M+H]^+$ calcd. for $C_{20}H_{26}IN_6O_2S$, 541.0883; found 541.0859.

6-Iodo-2,3-dihydrobenzofuran (37). A solution of 2,3-dihydrobenzofuran-6-amine (**36**; 0.74 g, 5.5 mmol) in acetic acid (25 mL) and TFA (2 mL) was cooled in an ice bath for 5 minutes. $NaNO_2$ (0.454g, 6.6 mmol) was added in 3 portions followed by KI (2.73 g, 16.4 mmol). The resulting mixture was stirred at 0°C for 15 minutes and quenched with H_2O (20 mL). The mixture was extracted with EtOAc (3 x 150 mL) and the organic layer was washed with NaS_2O_3, brine, dried over $MgSO_4$ and filtered. The filtrate was condensed under reduced pressure and the residue was purified by flash chromatography (hexane:EtOAc, 90:10 to 40:60) to yield **37** (0.82 g, 61%) as a pale-yellow solid. ^1H NMR (500 MHz, $CDCl_3$) δ 7.14 (d, *J* = 7.6 Hz, 1H), 7.11 (s, 1H), 6.89 (d, *J* = 7.6 Hz, 1H), 4.54 (t, *J* = 8.7 Hz, 2H), 3.14 (t, *J* = 8.7 Hz, 2H); ^{13}C NMR (125 MHz, $CDCl_3$) δ 161.1, 129.4, 127.1, 126.4, 118.7, 91.7, 71.6, 29.4.

8-(2,3-Dihydrobenzofuran-6-ylthio)-9*H*-purin-6-amine (38). To a solution of **37** (50 mg, 0.2 mmol) in DMF (2 mL) was added 8-mercaptoadenine (34 mg, 0.2 mmol), Cs_2CO_3 (99.4 mg, 0.3 mmol) and $PdCl_2$(dppf) (33 mg, 0.02 mmol). The mixture was degassed for 5 minutes with argon and stirred at 80 °C under argon protection for 48 h. The resulting mixture was concentrated under reduced pressure and the residue was purified by flash chromatography (CH_2Cl_2:MeOH, 100:0 to 90:10) to yield **38** (25 mg, 44%) as a yellow solid. ^1H NMR (500 MHz, CD_3OD) δ 8.14 (s, 1H), 7.24 (d, *J* = 7.6 Hz, 1H), 7.07 (d, *J* = 7.3 Hz, 1H), 6.97 (s, 1H), 4.62 (t, *J* = 8.7 Hz, 2H), 3.25 (t, *J* = 8.7 Hz, 2H); MS (ESI) *m/z* 285.8 $[M+H]^+$; HRMS (ESI) *m/z* $[M+H]^+$ calcd. for $C_{13}H_{12}N_5OS$, 286.0763; found 286.0768.

8-(5-Iodo-2,3-dihydrobenzofuran-6-ylthio)-9*H*-purin-6-amine (39) To a solution of **38** (40 mg, 0.14 mmol) in 6 mL of acetonitrile was added TFA (40 µL) and NIS (63 mg, 0.28 mmol). The resulting mixture was stirred at rt for 2 h. The reaction mixture was concentrated under reduced pressure and the residue was purified by flash chromatography (CH_2Cl_2:MeOH, 100:0 to 90:10) to afford **39** (48 mg, 53%) as a yellow gum. ^1H NMR (500 MHz, $CDCl_3$) δ 8.26 (s, 1H), 7.79 (s, 1H), 7.12 (s, 1H), 4.65 (t, *J* = 8.8 Hz, 2H), 3.28 (t, *J* = 8.7 Hz, 2H); MS (ESI) *m/z* 412.0 $(M+H)^+$.

8-(5-Iodo-2,3-dihydro-benzofuran-6-ylsulfanyl)-9-(3-isopropylamino-propyl)-9*H*-purin-6-ylamine (40) (PU-WS4). A mixture of **39** (54 mg, 0.13 mmol), Cs_2CO_3 (127 mg, 0.39 mmol) and 1,3-dibromopropane (202 mg, 0.65 mmol) in anhydrous DMF (2 mL) was stirred at rt for 2 h. Solvent was removed under reduced pressure and the residue purified by chromatography (CH_2Cl_2:MeOH:AcOH). The resulting solid was dissolved in DMF (2 mL) and isopropylamine (0.347 g, 0.5 mL, 5.9 mmol) was added and the solution stirred overnight at rt. Solvent was removed under reduced pressure and the resulting residue was purified by chromatography (CH_2Cl_2:MeOH) to give **40** (13 mg, 20%; over two-steps) as a yellow solid. ^1H NMR (500 MHz, $CDCl_3/CD_3OD$) δ 8.26 (s, 1H), 7.77 (s, 1H), 7.07 (s, 1H), 4.65 (t, *J* = 8.7 Hz, 2H), 4.47 (t, *J* = 6.9 Hz, 2H), 3.20-3.33 (m, 3H), 3.01 (t, *J* = 7.5 Hz, 2H), 2.33 (m, 2H), 1.34 (d, *J* = 6.5 Hz, 6H); ^{13}C NMR (150 MHz, $CDCl_3/CD_3OD$) δ 162.2, 155.4, 153.1, 151.5, 147.2, 136.7, 133.9, 132.3, 120.0, 114.1, 90.7, 72.8, 51.3, 42.7, 41.2, 29.4, 27.3, 19.5; MS (ESI) *m/z* 511.2 $[M+H]^+$; HRMS (ESI) *m/z* $[M+H]^+$ calcd. for $C_{19}H_{24}IN_6OS$, 511.0777; found 511.0779.

8-(5-Iodo-2,3-dihydro-benzofuran-6-ylsulfanyl)-9-(2-isobutylamino-ethyl)-9*H*-purin-6-ylamine (86) (PU-WS9). A mixture of **39** (30 mg, 0.073 mmol), Cs_2CO_3 (71 mg, 0.22 mmol) and 1,2-dibromoethane (69 mg, 0.365 mmol) in anhydrous DMF (1 mL) was stirred at rt for 2 h. Solvent was removed under reduced pressure and the residue purified by chromatography (CH_2Cl_2:MeOH:AcOH). The resulting solid was dissolved in DMF (2 mL) and isobutylamine (0.241 g, 0.33 mL, 3.3 mmol) was added and the solution stirred overnight at rt. Solvent was removed under reduced pressure and the resulting residue was purified by chromatography (CH_2Cl_2:MeOH) to give **86** (15 mg, 40%; over two-steps) as a pale yellow solid. ^1H NMR (500 MHz, $CDCl_3$) δ 8.26 (s, 1H), 7.63 (s, 1H), 6.56 (s, 1H), 6.27 (br s, 2H), 4.57 (t, *J* = 8.5 Hz, 2H), 4.50 (t, *J* = 5.5 Hz, 2H), 3.20 (t, *J* = 8.5 Hz, 2H), 3.12 (t, *J* = 5.5 Hz, 2H), 2.59 (d, *J* = 7 Hz, 2H), 1.99 (m, 1H), 0.97 (d, *J* = 7 Hz, 6H); ^{13}C NMR (150 MHz, $CDCl_3/CD_3OD$) δ 161.5, 154.6, 153.0, 151.4, 146.4, 135.8, 135.4, 130.5, 119.9, 112.7, 88.6, 72.1, 57.4, 48.7, 43.8, 29.0, 28.2, 20.5; HRMS (ESI) *m/z* $[M+H]^+$ calcd. for $C_{19}H_{24}IN_6OS$, 511.0777; found 511.0790.

8-((5-iodo-2,3-dihydrobenzofuran-6-yl)thio)-9-(2-(neopentylamino)ethyl)-9H-purin-6-amine (87) (PU-WS21). A mixture of **39** (30 mg, 0.073 mmol), Cs_2CO_3 (71 mg, 0.22 mmol) and 1,2-dibromoethane (69 mg, 0.365 mmol) in anhydrous DMF (1 mL) was stirred at rt for 2 h. Solvent was removed under reduced pressure and the residue purified by chromatography (CH_2Cl_2:MeOH:AcOH). The resulting solid was dissolved in DMF (2 mL) and neopentylamine (0.288 g, 0.39 mL, 3.3 mmol) was added and the solution stirred overnight at rt. Solvent was removed under reduced pressure and the resulting residue was purified by chromatography (CH_2Cl_2:MeOH) to give **87** as a white solid. ^{1}H NMR (500 MHz, $CDCl_3$, δ): 7.56 (s, 1H), 6.60 (s, 1H), 4.47 (t, *J* = 8.7 Hz, 2H), 4.37 (m, 2H), 3.06-3.11 (m, 4H), 2.45 (s, 2H), 0.83 (s, 9H); ^{13}C NMR (150 MHz, $CDCl_3/CD_3OD$) δ 161.6, 154.7, 152.8, 151.3, 146.9, 135.9, 135.1, 130.9, 119.8, 113.1, 89.2, 72.3, 61.9, 49.7, 43.9, 31.6, 29.1, 27.7; HRMS (*m/z*): $[M+H]^+$ calcd for $C_{20}H_{26}IN_6OS$ 525.0933; found 525.0927.

5-Iodo-2,3-dihydrobenzofuran-6-amine (41). To a solution of 2,3-dihydrobenzofuran-6-amine (**36**; 95 mg, 0.7 mmol) in acetonitrile (3 mL) cooled in an ice-bath was added NIS (158 mg, 0.7 mmol). After stirring at 0 °C for 20 min, the mixture was condensed and purified by flash chromatography (hexane:EtOAc, 90:10 to 20:80) to yield **41** (180 mg, 98%) as a yellow solid. ^{1}H NMR (500 MHz, $CDCl_3$) δ 7.38 (s, 1H), 6.26 (s, 1H), 4.53 (t, *J* = 8.5 Hz, 2H), 3.09 (t, *J* = 8.4 Hz, 2H); MS (ESI) *m/z* 261.9 $[M+H]^+$.

8-(6-Amino-2,3-dihydrobenzofuran-5-ylthio)-9*H*-purin-6-amine (42). The mixture of **41** (80 mg, 0.31 mmol), 8-mercaptoadenine (52 mg, 0.3 mmol), neocuproine (7 mg, 0.03 mmol), CuI (7 mg, 0.03 mmol) and sodium *t*-butoxide (100 mg, 1.04 mmol) was suspended in 10 mL of DMF and stirred at 110 °C overnight. The mixture was concentrated under reduced pressure and the residue purified by flash chromatography (hexane:EtOAc, 90:10 to 20:80) to yield **42** (50 mg, 56%) as a pale yellow solid. ^{1}H NMR (500 MHz, $CDCl_3$) δ 8.15 (s, 1H), 7.33 (s, 1H), 6.35 (s, 1H), 4.57 (t, *J* = 8.5 Hz, 2H), 3.14 (t, *J* = 8.4 Hz, 2H); MS (ESI) *m/z* 301.0 $[M+H]^+$.

8-(6-Iodo-2,3-dihydrobenzofuran-5-ylthio)-9*H*-purin-6-amine (43). To a solution of **42** (25 mg, 0.08 mmol) in acetic acid/TFA (5 mL/1 mL) cooled in ice-bath was added $NaNO_2$ (7 mg, 0.1mmol) and KI (27 mg, 0.16 mmol). The mixture was stirred at 0°C for 10 minutes and condensed under reduced pressure. The residue was purified by flash chromatography (CH_2Cl_2:MeOH, 100:0 to 90:10) to yield **43** (13 mg, 41%) as a yellow solid. ^{1}H NMR (500 MHz, CD_3OD) δ 8.13 (s, 1H), 7.57 (s, 1H), 7.44 (s, 1H), 4.66 (t, *J* = 8.5 Hz, 2H), 3.22 (t, *J* = 8.6 Hz, 2H); MS (ESI) *m/z* 411.9 $[M+H]^+$.

9-(3-Bromopropyl)-8-(6-iodo-2,3-dihydrobenzofuran-5-ylthio)-9*H*-purin-6-amine (44). To a solution of **43** (13 mg, 0.03 mmol) in DMF (2 mL) was added 1,3-dibromopropane (16 µL, 0.16 mmol) and Cs_2CO_3 (20 mg, 0.06 mmol) and the resulting mixture was stirred at rt for 40 minutes. The mixture was condensed under reduced pressure and the residue was purified by flash chromatography to yield **44** (6.2 mg, 34%). ^{1}H NMR (500 MHz, $CDCl_3$) δ 8.31 (s, 1H), 7.37 (s, 2H), 6.00 (br s, 2H), 4.62 (t, *J* = 8.7 Hz, 2H), 4.36 (t, *J* = 6.7 Hz, 2H), 3.42 (t, *J* = 6.2 Hz, 2H), 3.18 (t, *J* = 8.9 Hz, 2H), 2.39 (m, 2H); MS (ESI) *m/z* 531.9/533.9 $[M+H]^+$.

8-(6-Iodo-2,3-dihydro-benzofuran-5-ylsulfanyl)-9-(3-isobutylamino-ethyl)-9*H*-purin-6-ylamine (45) (PU-WS10). A solution of **44** (6.2 mg, 0.012 mmol) and isopropylamine (0.2 mL) in DMF (1 mL) was stirred for 12 h. Solvent was removed under reduced pressure and the residue purified by preparative thin layer chromatography ($CHCl_3$:MeOH-NH_3 (7N), 20:1) to afford **45** (4.0 mg, 60%) as a pale yellow solid. ^{1}H NMR (400 MHz, $CDCl_3$) δ 8.27 (s, 1H), 7.69 (s, 1H), 7.27 (s, 1H), 5.93 (br s, 2H), 4.66 (t, *J* = 8.8 Hz, 2H), 4.29 (t, *J* = 7 Hz, 2H), 3.33 (t, *J* = 8.7 Hz, 2H), 2.74 (septet, *J* = 6.2 Hz, 1H), 2.58 (t, *J* = 6.8 Hz, 2H), 1.98 (m, 2H), 1.05 (d, *J* = 6.5 Hz, 6H); ^{13}C NMR (100 MHz, $CDCl_3$) δ 162.0, 154.4, 152.7, 151.9, 147.8, 141.7, 130.2, 128.6, 121.1, 120.0, 74.1, 71.7, 48.9, 44.0, 41.6, 30.9, 30.2; MS (ESI) *m/z* 511.1 $[M+H]^+$.

5-Amino-6-nitro-indane (47). A solution of 5-aminoindane (**46**; 10 g, 75 mmol) in 100 mL of dioxane cooled in ice bath was added acetic anhydride (15 mL) dropwise and kept stirring at room temperature for 2 days. The resulting mixture was condensed and dried under vacuum. The residue was dissolved in 100 mL of concentrated H_2SO_4, cooled in ice bath. KNO_3 in 15 mL of concentrated H_2SO_4 was added dropwise. The resulting solution was stirred at 0 °C for 2 h and then at rt for 2 h. The reaction mixture was poured into 150 g of ice and the resulting yellow precipitate was filtered and washed with cold water to give **47** (7.1 g, 43%). ^{1}H NMR (500 MHz, $CDCl_3$) δ 7.94 (s, 1H), 6.65 (s, 1H), 6.02 (br, 2H), 2.83 (m, 4H), 2.06 (m, 2H); ^{13}C NMR (125 MHz, $CDCl_3$) δ 154.4, 144.2, 134.1, 131.2, 120.8, 113.5, 33.1, 31.4, 25.7.

5-Iodo-6-nitro-indane (48). To a solution of **47** (0.14 g, 0.78 mmol) in acetic acid cooled in ice bath was added $NaNO_2$ (65 mg, 0.94 mmol). The reaction mixture was stirred for 2 minutes. KI (0.39g, 2.45 mmol) was added and the mixture was stirred at rt for 20 minutes. The resulting suspension was quenched with water (15 mL) and extracted with ethyl acetate (2 x 20 mL). The organic layer was washed with saturated aqueous $Na_2S_2O_3$ solution, brine and dried over MgSO4 and evaporated to dryness to give a residue that was purified by flash chromatography (ethyl acetate/hexane, gradient 0 to 50%) to give **48** (0.12 g, 65%) as a yellow solid. ^{1}H NMR (500 MHz, $CDCl_3$) δ 7.83 (s, 1H), 7.71 (s, 1H), 2.95 (m, 4H), 2.11 (m, 2H).

5-Amino-6-iodo-indane (49). To a solution of **48** (1.65 g, 5.7 mmol) in isopropanol (100 mL) and saturated aqueous NH_4Cl solution (20 mL) was added iron powder (1.1 g). The resulting suspension was refluxed for 1h. The reaction mixture was filtered and the filtrate was condensed and purified by flash chromatography (ethyl acetate/hexane, gradient 0 to 50%) to give **49** (1.36 g, 92%) as a pale yellow solid. ^{1}H NMR (500 MHz, $CDCl_3$) δ 7.44 (s, 1H), 6.59 (s, 1H), 3.88 (s, 2H), 2.74 (m, 4H), 1.98 (m, 2H); ^{13}C NMR (125 MHz, $CDCl_3$) δ 146.2, 144.9, 136.5, 134.1, 111.0, 32.8, 31.8, 26.1; MS (ESI): *m/z* 259.99 $[M+H]^+$.

8-((6-Amino-2,3-dihydro-1*H*-inden-5-yl)thio)-9-*H*-purin-6-amine (50). The mixture of 8-mercaptoadenine (64 mg, 0.38 mmol), **49** (100 mg, 0.38 mmol), CuI (14.7 mg, 0.07 mmol), sodium t-butoxide (111 mg, 1.15 mmol) and tetrabutylammonium bromide (24.9 mg, 0.07 mmol) in anhydrous DMF (4 mL) was vortexed and heated at 190 ^oC under microwave for 1h. The resulting mixture was condensed and purified by flash chromatography (methylene chloride/methanol, gradient 0 to 10%) to give **50** (54 mg, 47%) as a while solid. ^{1}HNMR (500 MHz, MeOH-*d₄*/$CDCl_3$) δ 8.11 (s, 1H), 7.36 (s, 1H), 6.81 (s, 1H), 2.85 (m, 4H), 2.06 (m, 2H); MS (ESI): *m/z* 299.02 $[M+H]^+$.

8-((6-Iodo-2,3-dihydro-1*H*-inden-5-yl)thio)-9-*H*-purin-6-amine (51). To a solution of **50** (54 mg, 0.18 mmol) in acetic acid (5 mL) cooled in ice bath was added $NaNO_2$ (15 mg, 0.22 mmol) followed by KI (90 mg, 0.54 mmol). The reaction mixture was stirred at 0 ^oC for 15 min and quenched with water (10 mL). The resulting mixture was extracted with methylene chloride (2 x 20 mL). The organic layer was washed with saturated aqueous $Na_2S_2O_3$, brine, dried over $MgSO_4$ and evaporated to dryness. The residue was purified by flash chromatography (methylene chloride/methanol, gradient 0 to 10%) to give **51** (42 mg, 56%) as a white solid. ^{1}HNMR (500 MHz, $CDCl_3$) δ 8.12 (s, 1H), 7.84 (s, 1H), 7.39 (s, 1H), 2.91 (m, 4H), 2.11 (m, 2H); MS (ESI) *m/z* 410.10 $[M+H]^+$.

9-(3-Bromopropyl)-8-((6-iodo-2,3-dihydro-1*H*-inden-5-yl)thio)-9*H*-purin-6-amine (52). To a solution of **51** (30 mg, 0.07 mmol) in DMF (3 mL) was added 1,3-dibromopropane (37 μL, 0.86 mmol) and Cs_2CO_3 (46 mg, 0.14 mmol). The resulting mixture was stirred at rt for 2 h. **52** (8 mg, 21%) was obtained following preparatory TLC (methylene chloride/methanol, 20/1) as a white solid. ^{1}H NMR (500 MHz, $CDCl_3$) δ 8.27 (s, 1H), 7.75 (s, 1H), 7.12 (s, 1H), 6.55 (br s, 2H), 4.33 (m, 2H), 2.88 (m, 2H), 2.79 (t, *J* = 7.4 Hz, 2H), 2.29 (m, 2H), 1.97 (m, 2H); MS (ESI): *m/z* 530.3, 532.3 $[M, M+2]^+$.

8-((6-Iodo-2,3-dihydro-1*H*-inden-5-yl)thio)-9-(3-(isopropylamino)propyl)-9*H*-purin-6-amine (53) PU-WS29). To a solution of **52** (8 mg, 0.015 mmol) in DMF (3 mL) was added isopropylamine (100 μL), stirred at rt overnight and condensed under vacuum. **53** (5.9 mg, 99%) was obtained following preparatory TLC (methylene chloride/methanol, 10/1) as a white solid. ^{1}H NMR (500 MHz, $CDCl_3$)δ 8.32 (s, 1H), 7.75 (s, 1H), 7.12 (s, 1H), 5.73 (br s, 2H), 4.29 (t, 2H), 2.87 (t, *J* = 7.4 Hz, 2H), 2.7-2.79 (m, 3H), 2.55 (t, 2H), 2.03-2.09 (m, 4H), 1.05 (d, *J* = 11.2 Hz, 6H); ^{13}C NMR (125 MHz, $CDCl_3$) δ 154.5, 152.9, 151.7, 147.2, 146.5, 135.9, 133.1, 127.6, 120.2, 97.9, 48.8, 43.7, 41.7, 32.5, 32.2, 30.0, 25.5, 22.7; HRMS (*m/z*): $[M+H]^+$ calcd for $C_{20}H_{26}IN_6S$ 509.0984; found 509.1003.

8-(3-Aminonaphthalen-2-ylthio)-9H-purin-6-amine (55). A mixture of 8-mercaptoadenine (20.7 mg, 0.124 mmol), neocuproine hydrate (3.9 mg, 0.0185 mmol), CuI (3.5 mg, 0.0185 mmol), sodium *tert*-butoxide (23.7 mg, 0.24 mmol), **54**[4] (100 mg, 0.37 mmol) and DMF (2 mL) were heated at 115 ˚C for 20 h. The solvent was removed under reduced pressure and the residue was purified by preparatory TLC (CH_2Cl_2:MeOH-NH_3 (7N), 10:1) to give 14 mg (37%) of **55** as a solid. ^{1}H NMR (500 MHz, $CDCl_3/CD_3OD$) δ 8.18 (s, 1H), 8.12 (s, 1H), 7.71 (d, *J* = 8.3 Hz, 1H), 7.62 (d, *J* = 8.1 Hz, 1H), 7.40-7.46 (m, 1H), 7.24-7.30 (m, 1H), 7.20 (s, 1H); MS (ESI) *m/z* 308.95 $[M+H]^+$.

8-(3-Iodonaphthalen-2-ylthio)-9H-purin-6-amine (56). To a suspension of **55** (14 mg, 0.0454 mmol) in water (150 μL) at 5 ˚C was added 6 M HCl (140 μL) over 5 min. Then a solution of $NaNO_2$ (6.3 mg, 0.0908 mmol) in water (70 μL) was added dropwise over 30 min. at below 5 ˚C. The mixture was stirred for an additional 10 min., then urea (1.9 mg, 0.0317 mmol) was added slowly. After 10 minutes, a solution of KI (22.6 mg, 0.136 mmol) in

water (70 µL) was added dropwise over 5 min. and the mixture was stirred overnight. The solvent was removed under reduced pressure and the residue was purified by preparatory TLC (CH_2Cl_2:MeOH-NH_3 (7N), 8:1) to give 8 mg (42%) of **56** as a solid. ^{1}H NMR (500 MHz, $CDCl_3$/CD_3OD) δ 8.51 (s, 1H), 8.17 (s, 2H), 7.75-7.80 (m, 2H), 7.52-7.60 (m, 2H); MS (ESI) *m/z* 420.01 [M+H]$^+$.

9-(3-Bromopropyl)-8-(3-iodonaphthalen-2-ylthio)-9H-purin-6-amine (57). **56** (8 mg, 0.019 mmol), Cs_2CO_3 (7.4 mg, 0.0228 mmol), 1,3-dibromopropane (19.2 mg, 9.7 µL, 0.095 mmol) in DMF (0.2 mL) was stirred for 30 min. Then additional Cs_2CO_3 (7.4 mg, 0.0228 mmol) and 1,3-dibromopropane (19.2 mg, 9.7 µL, 0.095 mmol) was added and the mixture stirred for 30 min. The mixture was dried under reduced pressure and the residue purified by preparatory TLC (CH_2Cl_2:MeOH:AcOH, 15:1:0.5) to give 4.6 mg (45%) of **57**. ^{1}H NMR (500 MHz, $CDCl_3$/CD_3OD) δ 8.51 (s, 1H), 8.27 (s, 1H), 8.05 (s, 1H), 7.74-7.80 (m, 2H), 7.53-7.60 (m, 2H), 4.42 (t, J = 7.1 Hz, 2H), 3.45 (t, J = 6.6 Hz, 2H), 2.45 (m, 2H); MS (ESI) *m/z* 539.84/541.89 [M+H]$^+$.

8-(3-Iodonaphthalen-2-ylthio)-9-(3-(isopropylamino)propyl)-9H-purin-6-amine (58) (PU-DZ4-52-N9). **57** (4.6 mg, 0.0085 mmol) and isopropylamine (100 µL) in DMF (100 µL) was stirred overnight at rt. Solvent was removed under reduced pressure and the resulting residue was purified by preparatory TLC (CH_2Cl_2:MeOH-NH_3 (7N), 10:1) to give 4.0 mg (91%) of **58**. ^{1}H NMR (500 MHz, $CDCl_3$) δ 8.44 (s, 1H), 8.34 (s, 1H), 7.77 (s, 1H), 7.70-7.74 (m, 1H), 7.64-7.68 (m, 1H), 7.45-7.54 (m, 2H), 4.36 (t, J = 6.9 Hz, 2H), 2.74 (septet, J = 6.1 Hz, 1H), 2.58 (t, J = 6.8 Hz, 2H), 2.06 (m, 2H), 1.05 (d, J = 6.3 Hz, 6H); ^{13}C NMR (125 MHz, $CDCl_3$ /CD_3OD) δ 157.7, 155.6, 155.2, 151.9, 140.6, 139.9, 137.7, 136.4, 133.4, 132.7, 131.2, 130.8, 123.0, 114.4, 52.6, 47.3, 45.2, 33.3, 25.9; HRMS (ESI) *m/z* [M+H]$^+$ calcd. for $C_{21}H_{24}IN_6S$, 519.0828; found 519.0815.

Preparation of 9-ethylamino and 9-propylamino purines 59-72, 74-81, 84 & 85. To a mixture of bromide **15** or **73** or **83** in DMF at rt was added the appropriate amine in excess and the reaction mixture was stirred for 8-12 h. When determined to be complete (by TLC or LC-MS), the solvent was removed under reduced pressure and the resulting residue was purified by chromatography on silica gel with a gradient of dichloromethane/methanol to give the desired compound.

2-Fluoro-8-((6-iodobenzo[d][1,3]dioxol-5-yl)methyl)-9-(3-(tert-butylamino)propyl)-9*H*-purin-6-amine (59) (PU-DZ4-80). **15** (5 mg, 0.0093 mmol) and *tert*-butylamine (68 mg, 98 µL, 0.93 mmol) in DMF (0.5 mL) was stirred overnight at rt. Solvent was removed under reduced pressure and the resulting residue was purified by preparatory TLC (hexane:CH_2Cl_2:EtOAc:MeOH-NH_3 (7N), 2:3:1:0.5) to give 2.2 mg (45%) of **59**. ^{1}H NMR (500 MHz, $CDCl_3$) δ 7.29 (s, 1H), 6.62 (s, 1H), 5.98 (br s, 2H), 5.97 (s, 2H), 4.25 (s, 2H), 4.18 (t, J = 6.6 Hz, 2H), 2.63 (t, J = 6.7 Hz, 2H), 2.06 (m, 2H), 1.21 (s, 9H); ^{13}C NMR (125 MHz, $CDCl_3$) δ 158.7 (d, J = 210.1 Hz), 156.3 (d, J = 19.8 Hz), 152.8(d, J = 19.0 Hz), 150.9 (d, J = 2.2 Hz), 148.9, 147.8, 131.7, 118.7, 116.7, 109.5, 101.9, 88.1, 50.6, 41.2, 39.2, 39.0, 30.9, 28.9; HRMS (ESI) *m/z* [M+H]$^+$ calcd. for $C_{20}H_{25}FIN_6O_2$, 527.1068; found 527.1077; HPLC: R_t = 8.25.

2-Fluoro-8-((6-iodobenzo[d][1,3]dioxol-5-yl)methyl)-9-(3-(isopropyl(methyl)amino)propyl)-9*H*-purin-6-amine (60) (PU-BSI12). **15** (39 mg, 0.073 mmol) was reacted with N-isopropylmethylamine (0.38 mL, 0.27 g, 3.65 mmol) in DMF (1 mL) and following purification **60** (28.7 mg, 75%) was obtained as a yellow solid. ^{1}H NMR (500 MHz, $CDCl_3$) δ 7.28 (s, 1H), 6.57 (s, 1H), 6.29 (br s, 2H), 5.94 (s, 2H), 4.26 (s, 2H), 4.04 (t, J = 7.5 Hz, 2H), 2.77 (m, 1H), 2.39 (t, J = 6.9 Hz, 2H), 2.13 (s, 3H), 1.87 (m, 2H), 0.96 (d, J = 6.5 Hz, 6H); ^{13}C NMR ($CDCl_3$/CD_3OD, 125 MHz) δ 158.8 (d, J = 208 Hz), 156.4 (d, J = 20 Hz), 152.8 (d, J = 20 Hz), 150.9, 148.9, 147.8, 131.9, 118.7, 116.9, 109.4, 101.9, 88.1, 53.4, 50.2, 41.6, 39.2, 36.3, 27.6, 17.7; MS (ESI): *m/z* 527.0 [M+H]$^+$.

9-(3-(Ethyl(isopropyl)amino)propyl)-2-fluoro-8-((6-iodobenzo[d][1,3]dioxol-5-yl)methyl)-9*H*-purin-6-amine. (61) (PU-BSI8). **15** (37 mg, 0.069 mmol) was reacted with N-ethylisopropylamine (0.42 mL, 0.3 g 3.5 mmol) in DMF (1 mL) and following purification **61** (27 mg, 72%) was obtained as a pale-yellow solid. ^{1}H NMR (500 MHz, $CDCl_3$) δ 7.28 (s, 1H), 6.59 (s, 1H), 6.07 (br s, 2H), 5.95 (s, 2H), 4.24 (s, 2H), 4.05 (t, J = 7.6 Hz, 2H), 2.91 (m, 1H), 2.42-2.46 (m, 4H), 1.84 (m, 2H), 1.03 (t, J = 7.1 Hz, 3H), 0.99 (d, J = 6.5 Hz, 6H); ^{13}C NMR ($CDCl_3$/CD_3OD, 125 MHz) δ 158.8 (d, J = 209 Hz), 156.2 (d, J = 20 Hz), 152.5 (d, J = 20 Hz), 150.6, 148.9, 147.9, 131.4, 118.8, 116.1, 109.8, 101.9, 88.3, 50.2, 46.5, 43.7, 41.6, 39.2, 28.4, 18.0, 13.6; MS (ESI): *m/z* 541.0 [M+H]$^+$.

9-(3-(Ethyl(methyl)amino)propyl)-2-fluoro-8-((6-iodobenzo[d][1,3]dioxol-5-yl)methyl)-9H-purin-6-amine (62) (PU-BSI13). **15** (35 mg, 0.065 mmol) was reacted with N-ethylmethylamine (0.28 mL, 0.19 g, 3.28 mmol) in

DMF (1 mL) and following purification **62** (26 mg, 78%) was obtained as a yellow solid. ^{1}H NMR (500 MHz, $CDCl_3/CD_3OD$) δ 7.30 (s, 1H), 6.66 (s, 1H), 5.98 (s, 2H), 4.27 (s, 2H), 4.08 (t, *J* = 7.3 Hz, 2H), 2.41 (m, 4H), 2.19 (s, 3H), 1.94 (m, 2H), 1.04 (t, *J* = 7.1 Hz, 3H); ^{13}C NMR ($CDCl_3$/ CD_3OD, 125 MHz) δ 158.8 (d, *J* = 209 Hz), 156.3 (d, *J* = 19 Hz), 152.4 (d, *J* = 19 Hz), 150.7, 148.9, 147.9, 131.4, 118.7, 116.1, 109.7, 101.9, 88.2, 53.7, 51.2, 41.4, 41.1, 39.0, 26.8, 11.7; MS (ESI): *m/z* 513.0 $[M+H]^+$.

9-(3-((Cyclopropylmethyl)(propyl)amino)propyl)-2-fluoro-8-((6-iodobenzo[d][1,3]dioxol-5-yl)methyl)-9*H*-purin-6-amine (63) (PU-BSI14). 15 (35 mg, 0.066 mmol) was reacted with N-(cyclopropylmethyl)propan-1-amine (0.47 mL, 0.37 g, 3.3 mmol) in DMF (1 mL) and following purification **63** (29.6 mg, 80%) was obtained as a yellow solid. ^{1}H NMR (500 MHz, $CDCl_3$) δ 7.27 (s, 1H), 6.53 (s, 1H), 6.35 (br s, 2H), 5.94 (s, 2H), 4.31 (s, 2H), 4.04 (t, *J* = 7.5 Hz, 2H), 2.53 (t, *J* = 7.1 Hz, 2H), 2.41 (t, *J* = 7.6 Hz, 2H), 2.27 (d, *J* = 6.5 Hz, 2H), 1.84 (m, 2H), 1.41 (m, 2H), 0.85 (t, *J* = 7.4 Hz, 3H), 0.78 (m, 1H), 0.45 (m, 2H), 0.04 (m, 2H). ^{13}C NMR ($CDCl_3/CD_3OD$, 125 MHz) δ 158.9 (d, *J* = 209 Hz), 156.3 (d, *J* = 19 Hz), 152.8 (d, *J* = 19 Hz), 151.0, 148.9, 147.8, 131.9, 118.7, 116.7, 109.3, 101.9, 88.0, 58.7, 55.9, 50.8, 41.8, 39.1, 27.2, 19.9, 12.0, 8.5, 3.9; MS (ESI) *m/z* 567.0 $[M+H]^+$.

2-Fluoro-8-((6-iodobenzo[d][1,3]dioxol-5-yl)methyl)-9-(3-(methyl)(prop-2-ynyl)amino)propyl)-9*H*-purin-6-amine (64) (PU-BSI11). 15 (30 mg, 0.056 mmol) was reacted with N-methylpropargylamine (0.193 g, 2.8 mmol) in DMF (0.8 mL) and following purification **64** (5.8 mg, 20%) was obtained as a yellow solid. ^{1}H NMR (500 MHz, $CDCl_3$) δ 7.29 (s, 1H), 6.57 (s, 1H), 5.95 (s, 2H), 5.73 (br s, 2H), 4.28 (s, 2H), 4.05 (t, *J* = 7.2 Hz, 2H), 3.33 (s, 2H), 2.40 (t, *J* = 6.7 Hz, 2H), 2.27 (s, 3H), 2.20 (s, 1H), 1.91 (m, 2H); ^{13}C NMR ($CDCl_3$, 125 MHz) δ 158.8 (d, *J* = 208 Hz), 156.4 (d, *J* = 20 Hz), 152.8 (d, *J* = 20 Hz), 151.2, 148.9, 147.8, 131.9, 118.7, 116.6, 109.4, 101.9, 88.1, 51.9, 45.5, 41.5, 41.3, 39.2, 27.0. MS (ESI): *m/z* 522.9 $[M+H]^+$.

9-(3-Allyl(methyl)amino)propyl)-2-fluoro-8-((6-iodobenzo[d][1,3]dioxol-5-yl)methyl)-9*H*-purin-6-amine (65) (PU-BSI7). 15 (40 mg, 0.075 mmol) was reacted with N-allylmethylamine (0.148 mL, 107 mg, 1.5 mmol) in DMF (0.8 mL) and following purification **65** (31 mg, 79%) was obtained as a yellow solid. ^{1}H NMR (500 MHz, $CDCl_3/CD_3OD$) δ 7.30 (s, 1H), 6.66 (s, 1H), 5.98 (s, 2H), 5.81 (m, 1H), 5.13-5.15 (m, 2H), 4.25 (s, 2H), 4.09 (t, *J* = 7.4 Hz, 2H), 2.97 (d, *J* = 6.6 Hz, 2H), 2.39 (t, *J* = 7 Hz, 2H), 2.19 (s, 3H), 1.95 (m, 2H); ^{13}C NMR ($CDCl_3/CD_3OD$, 125 MHz) δ 158.8 (d, J = 209 Hz), 156.2 (d, J = 20 Hz), 152.5 (d, J = 20 Hz), 150.6, 148.9, 147.9, 134.9, 131.4, 118.7, 118.0, 116.1, 109.7, 101.9, 88.2, 60.7, 53.7, 41.8, 41.4, 39.1, 27.0; MS (ESI): *m/z* 524.9 $[M+H]^+$.

2-Fluoro-8-((6-iodobenzo[d][1,3]dioxol-5-yl)methyl)-9-(3-(2-methylaziridin-1-yl)propyl)-9*H*-purin-6-amine (66) (PU-BSI10). 15 (40 mg, 0.075 mmol) was reacted with 2-methylaziridine (0.053 mL, 43 mg, 0.75 mmol) in DMF (0.8 mL) and following purification **66** (20.9 mg, 55%) was obtained as a yellow solid. ^{1}H NMR (500 MHz, $CDCl_3$) δ 7.28 (s, 1H), 6.55 (s, 1H), 6.37 (br s, 2H), 5.95 (s, 2H), 4.34 (s, 2H), 4.12 (m, 2H), 2.22 (m, 2H), 1.94 (m, 2H), 1.48 (d, *J* = 3.5 Hz, 1H), 1.32 (m, 1H), 1.16 (m, 1H), 1.15 (d, *J* = 5.5 Hz, 3H); ^{13}C NMR ($CDCl_3$, 125 MHz) δ 158.8 (d, *J* = 208 Hz), 156.4 (d, *J* = 20 Hz), 152.8 (d, *J* = 20 Hz), 151.1, 148.9, 147.8, 131.9, 118.7, 116.6, 109.4, 101.9, 88.1, 57.7, 41.5, 39.0, 34.7, 34.6, 29.7, 18.2; MS (ESI): *m/z* 511.1 $[M+H]^+$.

2-Fluoro-8-((6-iodobenzo[d][1,3]dioxol-5-yl)methyl)-9-(3-(piperidine-1-yl)propyl)-9*H*-purin-6-amine. (67) (PU-BSI15). 15 (30 mg, 0.056 mmol) was reacted with piperidine (0.011 mL, 9.5 mg, 0.112 mmol) in DMF (0.8 mL) and following purification **67** (12.1 mg, 40%) was obtained as a yellow solid. ^{1}H NMR (500 MHz, $CDCl_3$) δ 7.26 (s, 1H), 6.56 (s, 1H), 5.95 (s, 2H), 5.84 (br s, 2H), 4.31 (s, 2H), 4.05 (t, *J* = 7.2 Hz, 2H), 2.29 (m, 6H), 1.94 (m, 2H), 1.54 (m, 4H), 1.41 (m, 2H); ^{13}C NMR ($CDCl_3/CD_3OD$, 125 MHz) δ 158.6 (d, *J* = 157 Hz) , 155.9 (d, *J* = 19 Hz), 152.2 (d, *J* = 19 Hz), 150.6, 148.8, 147.8, 131.3, 118.6, 116.1, 110.0, 101.8, 88.2, 55.6, 54.3, 41.4, 39.0, 26.2, 25.4, 24.0; MS (ESI): *m/z* 539.0 $[M+H]^+$.

2-Fluoro-8-((6-iodobenzo[d][1,3]dioxo-5-yl)methyl)-9-(3-morpholinopropyl)-9*H*-purin-6-amine (68) (PU-BSI16). 15 (30 mg, 0.056 mmol) was reacted with morpholine (0.098 mL, 9.8 mg, 0.112 mmol) in DMF (0.8 mL) and following purification **68** (5.8 mg, 19.2%) was obtained as a yellow solid. ^{1}H NMR (500 MHz, $CDCl_3$) δ 7.29 (s, 1H), 6.55 (s, 1H), 5.95 (s, 2H), 5.85 (br s, 2H), 4.27 (s, 2H), 4.07 (t, *J* = 7.0 Hz, 2H), 3.66 (t, *J* = 4.6 Hz, 4H), 2.31-2.35 (m, 6H), 1.92 (m, 2H); ^{13}C NMR ($CDCl_3$, 125 MHz) δ 158.8 (d, *J* = 210 Hz), 156.2 (d, *J* = 20 Hz), 152.4 (d, *J* = 19 Hz), 151.1, 148.8, 147.8, 131.8, 118.7, 116.9, 109.1, 101.9, 87.5, 66.9, 55.6, 53.6, 41.4, 39.0, 25.8; MS (ESI): *m/z* 540.9 $[M+H]^+$.

2-((3-(6-Amino-2-fluoro-8-((6-iodobenzo[d][1,3]dioxol-5-yl)methyl)-9*H*-purin-9-yl)propyl)(isopropyl)amino)ethanol (69) (PU-BSI6). **15** (36 mg, 0.067 mmol) was reacted with 2-(isoproylamino)ethanol (0.39 mL, 0.35 g, 3.37 mmol) in DMF (1 mL) and following purification **69** (17.7 mg, 46%) was obtained as a yellow solid. ^{1}H NMR (500 MHz, $CDCl_3/CD_3OD$) δ 7.31 (s, 1H), 6.70 (s, 1H), 5.99 (s, 2H), 4.24 (s, 2H), 4.09 (t, *J* = 7.9 Hz, 2H), 3.54 (t, *J* = 5.3 Hz, 2H), 2.97 (m, 1H), 2.58 (t, *J* = 5.3 Hz, 2H), 2.54 (t, *J* = 7.2 Hz, 2H), 1.86 (m, 2H), 1.00 (d, *J* = 6.5 Hz, 6H); ^{13}C NMR($CDCl_3/CD_3OD$, 125 MHz) δ 158.5 (d, *J* = 221 Hz), 156.3 (d, *J* = 20 Hz), 152.3 (d, *J* = 19 Hz), 150.5, 148.9, 147.9, 131.1, 118.7, 116.0, 109.7, 101.9, 88.2, 58.8, 51.1, 50.6, 47.1, 41.2, 39.1, 28.7, 17.8; MS (ESI): *m/z* 556.9 $[M+H]^+$.

2-((3-6-Amino-2-fluoro-8-((6-iodobenzo[d][1,3]dioxo-5-yl)methyl)-9*H*-purin-9-yl)propyl)(cyclohexyl)amino)ethanol (70) (PU-BSI5). **15** (40 mg, 0.075 mmol) was reacted with N-cyclohexylethanolamine (1.07 g, 7.5 mmol) in DMF (1 mL) and following purification **70** (16.6 mg, 37%) was obtained as a pale-yellow solid. ^{1}H NMR (500 MHz, $CDCl_3$) δ 7.28 (s, 1H), 6.62 (s, 1H), 6.30 (br s, 2H), 5.95 (s, 2H), 4.24 (s, 2H), 4.03 (t, *J* = 7.6 Hz, 2H), 3.49 (t, *J* = 5.2 Hz, 2H), 2.59 (t, *J* = 5.3 Hz, 2H), 2.53 (t, *J* = 7 Hz, 2H), 2.43 (m, 1H), 1.61-1.79 (m, 8H), 1.15-1.21 (m, 4H); ^{13}C NMR ($CDCl_3$, 125 MHz) δ 158.9 (d, *J* = 210 Hz), 156.3 (d, *J* = 20 Hz), 152.7 (d, *J* = 20 Hz), 150.8, 149.0, 147.9, 131.6, 118.7, 116.6, 109.5, 102.0, 88.0, 60.0, 59.0, 52.0, 47.7, 41.4, 39.2, 29.5, 29.0, 26.2, 26.1; MS (ESI): *m/z* 597.0 $[M+H]^+$.

2-(4-(3-(6-Amino-2-fluoro-8-((6-iodobenzo[d][1,3]dioxol-5-yl)methyl)-9*H*-purin-9-yl)propyl)peperazin-1-yl)ethanol (71) (PU-BSI3). **15** (40 mg, 0.075 mmol) was reacted with 1-(2-hydroxyethyl)piperazine (0.92 mL, 0.97 g, 7.5 mmol) in DMF (1 mL) and following purification **71** (14.9 mg, 34%) was obtained as a pale yellow solid. ^{1}H NMR (500 MHz, $CDCl_3$) δ 7.33 (s, 1H), 6.70 (s, 1H), 6.0 (s, 2H), 4.27 (s, 2H), 4.14 (t, *J* = 7.5 Hz, 2H), 3.67 (t, *J* = 5.8 Hz, 2H), 2.54 (t, *J* = 5.8 Hz, 2H), 2.38-2.60 (br s, 8H), 2.42 (t, *J* = 7.2 Hz, 2H), 1.98 (m, 2H); ^{13}C NMR ($CDCl_3/CD_3OD$, 125 MHz): δ 158.6 (d, *J* = 209 Hz), 156.3 (d, *J* = 20 Hz), 152.1 (d, *J* = 19 Hz), 150.6, 148.8, 147.9, 131.1, 118.6, 115.9, 109.6, 101.9, 88.1, 59.7, 58.2, 54.9, 52.7, 49.0, 41.3, 38.9, 26.1; MS (ESI): *m/z* 584.0 $[M + H]^+$.

3-(6-Amino-2-fluoro-8-((6-iodobenzo[d][1,3]dioxol-5-yl)methyl)-9*H*-purin-9-yl)-*N,N,N*-trimethylpropan-1-aminium (72) (PU-BSI4). **15** (40 mg, 0.075 mmol) was reacted with trimethylamine (1.5 g, 31-35% in ethanol) in DMF (0.8 mL) and following purification **72** was obtained as a pale-yellow solid in quantitative yield. ^{1}H NMR (500 MHz, $CDCl_3/CD_3OD$) δ 7.33 (s, 1H), 6.90 (s, 1H), 6.02 (s, 2H), 4.36 (s, 2H), 4.27 (t, *J* = 7.3 Hz, 2H), 3.59 (m, 2H), 3.22 (s, 9H), 2.34 (m, 2H); ^{13}C NMR ($CDCl_3/CD_3OD$, 125 MHz) δ 158.5 (d, *J* = 210 Hz), 156.4 (d, *J* = 20 Hz), 151.7 (d, *J* = 19 Hz), 150.6, 148.7, 147.7, 131.2, 118.4, 115.9, 110.2, 101.7, 88.5, 63.3, 53.2, 39.5, 38.6, 23.2; MS (ESI) *m/z* $[M+H]^+$ 513.0.

9-(2-Bromoethyl)-2-fluoro-8-((6-iodobenzo[d][1,3]dioxol-5-yl)methyl)-9H-purin-6-amine (73). A solution of **13**[1] (1.0 g. 2.4 mmol), Cs_2CO_3 (1.6 g, 4.8 mmol) and 1,2-dibromoethane (1.04 mL, 2.2 g, 12.1 mmol) in anhydrous DMF (45 mL) was stirred at rt for 5 h. Then additional Cs_2CO_3 (0.8 g, 2.4 mmol) was added and the reaction mixture was stirred for an additional 1 h. Solids were filtered and the filtrate was concentrated under reduced pressure. The residue was chromatographed (CH_2Cl_2/MeOH/AcOH, 240:1:0.5 to 200:1:0.5) to give 0.55 g of **73** (44%). ^{1}H NMR ($CDCl_3/CD_3OD$, 400 MHz) δ 7.30 (s, 1H), 6.64 (s, 1H), 5.97 (s, 2H), 4.42 (t, *J* = 6.6 Hz, 2H), 4.36 (s, 2H), 3.64 (t, *J* = 6.6 Hz, 2H); MS (ESI) *m/z* 520.0/522.1 $[M+H]^+$.

2-Fluoro-8-((6-iodobenzo[d][1,3]dioxol-5-yl)methyl)-9-(2-(isopropylamino)ethyl)-9*H*-purin-6-amine (74) (PU-DZ12). **73** (40 mg, 0.077 mmol) was reacted with isopropylamine (0.065 mL, 7.69 mmol) in DMF (0.8 mL) and following purification **74** (37 mg, 91%) was obtained as a yellow solid. ^{1}H NMR ($CDCl_3$, 400 MHz) δ 7.27 (s, 1H), 6.61 (s, 1H), 6.47 (br s, 2H), 5.94 (s, 2H), 4.29 (s, 2H), 4.09 (t, *J* = 6.5 Hz, 2H), 2.91 (t, *J* = 6.5 Hz, 2H), 2.75 (m, 1H), 0.98 (d, *J* = 6.2 Hz, 6H); ^{13}C NMR ($CDCl_3$, 100 MHz) δ 158.8 (d, *J* = 208 Hz), 156.4 (d, *J* = 20 Hz), 152.7 (d, *J* = 20 Hz), 151.10, 148.9, 147.8, 131.8, 118.7, 116.7, 109.6, 101.9, 88.1, 48.6, 46.0, 43.9, 39.4, 22.9; MS (ESI) *m/z* 498.9 $[M+H]^+$.

9-(2-(tert-Butylamino)ethyl)-2-fluoro-8-((6-iodobenzo[d][1,3]dioxol-5-yl)methyl)-9H-purin-6-amine (75) (PU-DZ4-81). **73** (8 mg, 0.0153 mmol) was reacted with *tert*-butylamine (112 mg, 161 μL, 1.53 mmol) in DMF (0.5 mL) and following purification **75** (4.3 mg, 55%) was obtained. ^{1}H NMR (500 MHz, $CDCl_3$) δ 7.29 (s, 1H), 6.60 (s, 1H), 5.95 (s, 2H), 5.83 (br s, 2H), 4.34 (s, 2H), 4.03 (t, *J* = 6.5 Hz, 2H), 2.86 (t, *J* = 6.5 Hz, 2H), 0.98 (s, 9H); ^{13}C NMR (151 MHz, $CDCl_3/CD_3OD$) δ 159.5 (d, *J* = 211 Hz), 156.6 (d, *J* = 19.6 Hz), 152.4 (d, *J* = 18.8 Hz),

151.3 (d, J = 2.4 Hz), 149.1, 148.1, 131.5, 118.9, 116.1 (d, J = 3.8 Hz), 110.2, 102.14, 88.5, 51.5, 43.7, 41.6, 39.3, 28.4; HRMS (ESI) m/z $[M+H]^+$ calcd. for $C_{19}H_{23}FIN_6O_2$, 513.0911; found 513.0915.

2-Fluoro-8-((6-iodobenzo[d][1,3]dioxol-5-yl)methyl)-9-(2-(isobutylamino)ethyl)-9*H*-purin-6-amine (76) (PU-DZ13). 73 (40 mg, 0.077 mmol) was reacted with isobutylamine (0.46 mL, 0.34 g, 4.6 mmol) in DMF (0.8 mL) and following purification **76** (12.4 mg, 32%) was obtained as a yellow solid. ^1H NMR ($CDCl_3$, 500 MHz) δ 7.29 (s, 1H), 6.61 (s, 1H), 5.96 (s, 2H), 5.71 (br s, 2H), 4.30 (s, 2H), 4.09 (t, J = 6.4 Hz, 2H), 2.90 (t, J = 6.4 Hz, 2H), 2.37 (d, J = 6.8 Hz, 2H), 1.62 (m, 1H), 0.86 (d, J = 6.7 Hz, 6H); ^{13}C NMR ($CDCl_3$, 125 MHz) δ 158.8 (d, J = 208 Hz), 156.3 (d, J = 20 Hz), 152.9 (d, J = 19 Hz), 151.3, 148.9, 147.6, 131.8, 118.7, 116.7, 109.5, 101.9, 88.2, 57.7, 48.8, 43.5, 39.3, 28.4, 20.6; MS (ESI) m/z 513.0 $[M+H]^+$.

2-Fluoro-8-((6-iodobenzo[d][1,3]dioxol-5-yl)methyl)-9-(2-(neopentylamino)ethyl)-9*H*-purin-6-amine (77) (PU-DZ14). 73 (40 mg, 0.077 mmol) was reacted with neopentylamine (0.67 g, 7.69 mmol) in DMF (0.8 mL) and following purification **77** (25.5 mg, 63%) was obtained as a yellow solid. ^1H NMR ($CDCl_3$, 500 MHz) δ 7.29 (s, 1H), 6.59 (s, 1H), 5.95 (s, 2H), 5.75 (br s, 2H), 4.30 (s, 2H), 4.08 (t, J = 6.1 Hz, 2H), 2.91 (t, J = 6.1 Hz, 2H), 2.29 (s, 2H), 0.84 (s, 9H); ^{13}C NMR ($CDCl_3/CD_3OD$, 125 MHz) δ 158.8 (d, J = 208 Hz), 156.3 (d, J = 20 Hz), 152.4 (d, J = 19 Hz), 151.1, 149.0, 148.0, 131.5, 118.8, 116.7, 109.9, 102.0, 88.4, 62.2, 49.6, 43.4, 39.2, 31.5, 27.7; MS (ESI) m/z 527.0 $[M+H]^+$.

9-(2-(Cyclopropylmethylamino)ethyl)-2-fluoro-8-((6-iodobenzo[d][1,3]dioxo-5-yl)methyl)-9*H*-purin-6-amine (78) (PU-DZ16). **73** (40 mg, 0.077 mmol) was reacted with cyclopropylmethylamine (0.67 mL, 0.547 g, 7.69 mmol) in DMF (0.8 mL) and following purification **78** (23.6 mg, 60%) was obtained as a yellow solid. ^1H NMR (500 MHz, $CDCl_3$) δ 7.28 (s, 1H), 6.62 (s, 1H), 6.03 (br s, 2H), 5.95 (s, 2H), 4.29 (s, 2H), 4.11 (t, J = 6.5 Hz, 2H), 2.94 (t, J = 6.5 Hz, 2H), 2.44 (d, J = 6.8 Hz, 2H), 0.87 (m, 1H), 0.44 (m, 2H), 0.08 (m, 2H); ^{13}C NMR ($CDCl_3$, 125 MHz) δ 158.8 (d, J = 209 Hz), 156.4 (d, J = 19 Hz), 152.9 (d, J = 19 Hz), 151.1, 148.9, 147.8, 131.8, 118.7, 116.7, 109.6, 101.9, 88.1, 54.7, 48.4, 43.5, 39.3, 11.2, 3.4; MS (ESI) m/z 510.9 $[M+H]^+$.

9-(2-(Ethyl(methyl)amino)ethyl)-2-fluoro-8-((6-iodobenzo[d][1,3]dioxol-5-yl)methyl)-9*H*-purin-6-amine (79) (PU-DZ15). 73 (40 mg, 0.077 mmol) was reacted with N-ethylmethylamine (0.66 mL, 0.45 g, 7.69 mmol) in DMF (0.8 mL) and following purification **79** was obtained as a yellow solid (14.6 mg, 38 %). ^1H NMR (500 MHz, $CDCl_3$) δ 7.29 (s, 1H), 6.57 (s, 1H), 6.12 (br s, 2H), 5.95 (s, 2H), 4.27 (s, 2H), 4.09 (t, J = 6.6 Hz, 2H), 2.66 (t, J = 6.6 Hz, 2H), 2.43 (q, J = 7.1 Hz, 2H), 2.27 (s, 3H), 0.94 (t, J = 7.1 Hz, 3H); ^{13}C NMR ($CDCl_3/CD_3OD$, 125 MHz) δ 158.8 (d, J = 208 Hz), 156.2 (d, J = 20 Hz), 152.8 (d, J = 19 Hz), 151.3, 148.9, 147.8, 131.9, 118.7, 116.8, 109.5, 101.9, 88.1, 55.8, 51.7, 42.1, 41.6, 39.3, 12.3; MS (ESI) m/z 498.9 $[M+H]^+$.

2-Fluoro-8-((6-iodobenzo[d][1,3]dioxol-5-yl)methyl)-9-(2-(isobutyl(methyl)amino)amino)ethyl)-9*H*-purin-6-amine (80) (PU-DZ17). 73 (40 mg, 0.077 mmol) was reacted with isobutylmethylamine (0.92 mL, 0.67 g, 7.69 mmol) in DMF (0.8 mL) and following purification **80** (19.3 mg, 48%) was obtained as a yellow solid. ^1H NMR (500 MHz, $CDCl_3$) δ 7.29 (s, 1H), 6.55 (s, 1H), 6.02 (br s, 2H), 5.95 (s, 2H), 4.26 (s, 2H), 4.06 (t, J = 6.5 Hz, 2H), 2.62 (t, J = 6.5 Hz, 2H), 2.25 (s, 3H), 2.04 (d, J = 7.3 Hz, 2H), 1.52 (m, 1H), 0.73 (d, J = 6.5 Hz, 6H); ^{13}C NMR ($CDCl_3$, 125 MHz) δ 158.8 (d, J = 213 Hz), 156.3 (d, J = 19 Hz), 152.9 (d, J = 19 Hz), 151.2, 148.8, 147.7, 131.9, 118.7, 116.8 (d, J = 4 Hz), 109.5, 101.9, 88.1, 66.8, 56.8, 42.9, 41.6, 39.4, 26.3, 20.6; MS (ESI) m/z 527.0 $[M+H]^+$.

2-Fluoro-8-((6-iodobenzo[d][1,3]dioxo-5-yl)methyl)-9-(2-(methyl(prop-2-ynyl)amino)ethyl)-9*H*-purin-6-amine (81) (PU-DZ18). **73** (40 mg, 0.077 mmol) was reacted with N-methylpropargylamine (0.38 mL, 0.32 g, 4.6 mmol) in DMF (0.8 mL) and following purification **81** (13.6 mg, 35%) was obtained as a yellow solid. ^1H NMR (500 MHz, $CDCl_3$) δ 7.31 (s, 1H), 6.68 (s, 1H), 5.99 (s, 2H), 4.26 (s, 2H), 4.15 (t, J = 6.4 Hz, 2H), 3.35 (s, 2H), 2.80 (t, J = 6.4 Hz, 2H), 2.36 (s, 3H), 2.05 (s, 1H); ^{13}C NMR ($CDCl_3/CD_3OD$, 125 MHz) δ 158.7 (d, J = 213 Hz), 156.2 (d, J = 19 Hz), 153.5 (d, J = 19 Hz), 150.9, 148.9, 147.9, 131.5, 118.7, 116.1 (d, J = 4 Hz), 109.9, 101.9, 88.3, 78.1, 73.5, 54.4, 46.2, 42.0, 41.3, 39.2; MS (ESI) m/z 508.9 $[M+H]^+$.

9-(2-Bromoethyl)-8-(6-iodobenzo[d][1,3]dioxol-5-ylthio)-9H-purin-6-amine (83). A solution of **82**[1] 1.0 g. 2.42 mmol), Cs_2CO_3 (1.2 g, 3.63 mmol) and 1,2-dibromoethane (1.04 mL, 2.2 g, 12.1 mmol) in anhydrous DMF (30 mL) was stirred at rt for 1 h. Then additional Cs_2CO_3 (0.4 g, 1.21 mmol) was added and the reaction mixture was stirred for an additional 30 minutes. This was repeated two more times, then solids were filtered and the filtrate

was concentrated under reduced pressure. The residue was purified by chromatography ($CHCl_3$/MeOH/AcOH, 150:1:0.5 to 120:1:0.5) to give 0.50 g of **83** (39%). ^1H NMR ($CDCl_3$/CD_3OD, 500 MHz) δ 8.24 (s, 1H), 7.37 (s, 1H), 7.07 (s, 1H), 6.04 (s, 2H), 4.65 (t, *J* = 6.7 Hz, 2H), 3.75 (t, *J* = 6.7 Hz, 2H); MS (ESI) *m/z* 519.6/521.7 $[M+H]^+$.

8-(6-Iodobenzo[d][1,3]dioxol-5-ylthio)-9-(2-(isobutylamino)ethyl)-9*H*-purin-6-amine (84) (PU-HZ150). 83 (20 mg, 0.0384 mmol) was reacted with isobutylamine (0.385 mL, 0.281 g, 3.84 mmol) in DMF (0.4 mL) and following purification **84** was obtained as a yellow solid (10.2 mg, 51%). ^1H NMR ($CDCl_3$/CD_3OD, 500 MHz) δ 8.23 (s, 1H), 7.36 (s, 1H), 7.04 (s, 1H), 6.04 (s, 2H), 4.35 (t, *J* = 6.3 Hz, 2H), 3.00 (t, *J* = 6.3 Hz, 2H), 2.44 (d, *J* = 6.9 Hz, 2H), 1.72 (septet, *J* = 7 Hz, 1H), 0.88 (d, *J* = 6.7 Hz, 6H); ^{13}C NMR($CDCl_3$/CD_3OD, 125 MHz) δ 154.5, 152.6, 151.3, 149.4, 149.3, 147.3, 126.9, 119.6, 119.3, 113.3, 102.5, 93.0, 57.4, 48.6, 43.7, 28.2, 20.5; MS (ESI) *m/z* 512.9 $[M+H]^+$.

8-(6-Iodobenzo[d][1,3]dioxol-5-ylthio)-9-(2-(neopentylamino)ethyl)-9*H*-purin-6-amine (85) (PU-HZ151). 83 (20 mg, 0.038 mmol) was reacted with neopentylamine (335 mg, 3.84 mmol) in DMF (0.4 mL) and following purification **85** (19.7 mg, 97%) was obtained as a yellow solid. ^1H NMR (500 MHz, $CDCl_3$) δ 8.33 (s, 1H), 7.29 (s, 1H), 6.89 (s, 1H), 5.98 (s, 2H), 5.70 (br s, 2H), 4.33 (t, *J* = 6.0 Hz, 2H), 2.99 (t, *J* = 5.9 Hz, 2H), 2.34 (s, 2H), 0.84 (s, 9H); ^{13}C NMR ($CDCl_3$/CD_3OD, 125 MHz) δ 154.17, 152.15, 151.01, 149.45, 149.19, 147.69, 126.36, 119.24, 119.20, 113.35, 102.35, 93.29, 61.75, 49.35, 43.58, 31.24, 27.35; MS (ESI) *m/z* 527.0 $[M+H]^+$.

9-(2-(Neopentylamino)ethyl)-8-((6-(trimethylstannyl)benzo[d][1,3]dioxol-5-yl)thio)-9H-purin-6-amine (PU-HZ151-Sn). To **85** (25 mg, 0.047 mmol), $Pd(PPh_3)_4$ (2.7 mg, 0.0023 mmol), hexamethylditin (38.9 µL, 0.232 mmol) was added 3 mL of dry dioxane and the reaction mixture was heated at 90°C in a 10 mL RBF sealed with a rubber septum for 20 hours. The solvent was concentrated under vacuum and the crude product was purified via preparatory TLC twice; first time using $CHCl_3$:EtOAc:hexane:NH_3/MeOH (7N) at 2:1:2:0.5, and then by CH_2Cl_2:MeOH at 9:1 to afford **PU-HZ151-Sn** (7.4 mg, 28 %). ^1H NMR (500 MHz, CD_3OD) δ 8.14 (s, 1H), 7.12 (s, 1H), 7.05 (s, 1H), 6.05 (s, 2H), 4.34 (t, *J* = 6.6 Hz, 2H), 3.04 (t, *J* = 6.6 Hz, 2H), 2.41 (s, 2H), 0.89 (s, 9H), 0.26 (s, 9H); ^{13}C NMR (151 MHz, $CDCl_3$/CD_3OD) δ 154.00, 151.84, 151.63, 150.67, 149.66, 144.65, 134.36, 126.35, 119.22, 119.19, 115.88, 101.81, 61.97, 49.40, 43.33, 31.53, 27.65, -7.63; MS (*m/z*): $[M+H]^+$ 565.2.

[$^{124/131}$I]-PU-HZ151. Radioactive iodine-124 in the form of [^{124}I]-NaI dissolved in 0.1 N NaOH was synthesized at MSKCC or purchased from 3D Imaging (Littlerock, AR). Radioactive iodine-131 in the form of [^{131}I]-NaI dissolved in 0.1 N NaOH was purchased from Cardinal Health (Dublin, OH). **PU-HZ151-Sn** (25 µg, 0.044 µmol) in an eppendorf tube was dissolved in 50 µL of ethanol, was briefly vortexed and centrifuged for 20 seconds at 500 RPM. To the resulting solution, 0.5 – 5 mCi of [$^{124/131}$I]-NaI in 0.1 N sodium hydroxide was added followed by addition of 2 µL of chloramine-T solution (2 mg/mL in acetic acid) and the reaction mix was allowed to react for 2 minutes. The resulting solution was diluted to a total volume of 200 µL with water and loaded on to HPLC and purified by passing through C-18 250 x 4.6 mm, RP HPLC column, using an isocratic elution at 30 % CH_3CN (0.1% TFA) in water as the eluant with a flow rate of 1 ml/min. The product which has a retention time of about 6.3 minutes under the conditions described above, was collected and solvent was removed under reduced pressure. The pure compound was formulated in 0.9% saline and used for further studies.

References

1. He, H. *et al.* Identification of potent water soluble purine-scaffold inhibitors of the heat shock protein 90. *J. Med. Chem.* **49**, 381-390 (2006).
2. Taldone, T. *et al.* Radiosynthesis of the iodine-124 labeled Hsp90 inhibitor PU-H71. *J. Labelled Comp. Radiopharm.* **59**, 129-132 (2016).
3. Taldone, T. *et al.* Preparation of a Diverse Purine-Scaffold Library via One-Step Palladium Catalyzed Cross-Coupling. *Heterocycles* **87**, 91-113 (2013).
4. Baudoin, O. *et al.* Synthesis and biological evaluation of A-ring biaryl-carbamate analogues of rhazinilam. *Bioorg. Med. Chem.* **10**, 3395-3400 (2002).

Supplementary Note 2. Clinical study protocol

PROTOCOL
PU-AD-00-001

**TITLE:**	PET Imaging of Subjects Using ^{124}I-PU-AD: A Pilot Study
**PRODUCT:**	^{124}I-PU-AD
**STUDY SPONSOR:**	**Samus Therapeutics, Inc.**<br>10 South Main Street<br>Topsfield, MA 01983<br>Phone: (646) 838-0624<br>Fax: (917) 322-2105<br><br>**Primary Sponsor Contacts**<br>Robert A. Morgan<br>10 South Main Street<br>Topsfield, MA 01983<br>Phone: (646) 838-0624
**MEDICAL MONITOR:**	**Michael H. Silverman, MD**<br>Samus Therapeutics, Inc.<br>10 South Main Street<br>Topsfield, MA 01983<br>Phone: (781) 631-8596
**AMENDMENT:**	7.0
**DATE:**	07-November-2017
**PREVIOUS VERSION/S (DATE):**	Amendment 6: 24-October-2017<br>Amendment 5: 26-September-2017<br>Amendment 4: 15-June-2017<br>Amendment 3: 03-March-2017<br>Amendment 2: 01-September-2016<br>Amendment 1: 28-June-2016<br>Original: 19-April-2016
**CONFIDENTIALITY STATEMENT**:	This document is confidential. It contains proprietary information of Samus Therapeutics, Inc. Any viewing or disclosure of such information that is not authorized in writing by Samus Therapeutics, Inc. is strictly prohibited. Such information may be used solely for the purpose of reviewing or conducting this protocol.

Memorial Sloan Kettering Cancer Center
IRB Number 16-004 A(9)
Approval date: 03-Feb-2020

SPONSOR AUTHORIZATION

**PROTOCOL NUMBER:**	PU-AD-00-001
**TITLE:**	PET Imaging of Subjects Using ^{124}I-PU-AD: A Pilot Study
**AMENDMENT:**	7.0
**DATE:**	07-November- 2017

07 November 2017

Robert A. Morgan
Chief Regulatory/Quality/Contracting Officer
Samus Therapeutics, Inc.
10 South Main Street
Topsfield, MA 01983

Date of Signature

PROTOCOL SYNOPSIS

**Protocol**	PU-AD-00-001
**Title**	PET Imaging of Subjects Using ^{124}I-PU-AD: A Pilot Study
**Product**	^{124}I-PU-AD
**Phase**	0
**Population**	Subjects with a confirmed diagnosis of specific cancer types, including solid malignancy, lymphoma, and myeloma, and/or Alzheimer's Disease.
**Objectives**	**Primary Objectives:**<br>• Determine the pharmacokinetics (PK), metabolism, biodistribution, and radiation dosimetry of ^{124}I-PU-AD in subjects with specific cancer types (solid malignancy, lymphoma, and/or myeloma) and/or Alzheimer's disease.<br><br>**Secondary Objectives:**<br>• Determine the safety of ^{124}I-PU-AD in this population, as determined through recording of adverse events.
**Design**	This first in-human trial of the positron-emitting agent ^{124}I-PU-AD is an open-label pilot study.<br><br>Up to 10 evaluable subjects who have active disease will be enrolled to evaluate the PK, metabolism, biodistribution, and radiation dosimetry of ^{124}I-PU-AD.<br><br>Following a 28-day screening period as outlined in Table 1, eligible subjects will return to the clinic on Day 1. A single dose of ^{124}I-PU-AD will be administered by intravenous (IV) injection to subjects. The formulation includes a microgram PU-AD dose of up to 10.0 mCi (minimal toxicity risk) with a predicted radiation exposure comparable to that of standard radiology exams. Subjects are prescribed a standard clinical oral iodine regimen to reduce thyroid exposure. Seven drops of saturating solution of potassium iodide (SSKI) will be given to the patient orally, daily, for two weeks; the first oral dose occurring the day before or at least 2 hours prior to the administration of ^{124}I-PU-AD.<br><br>For Alzheimer's Disease patients, the patient's individual legally authorized representative (LAR) will assume responsibility for ensuring adherence to the daily SSKI dosing regimen prescribed, documenting the time and date of each ingested dose of SSKI in a study medication journal. The site staff will contact the responsible caregiver prior to ^{124}I-PU-AD tracer administration and on the 2nd day of ^{124}I-PU-AD PET scanning to ensure SSKI compliance. The prescribed daily dose of 7 SSKI droplets can be added to a glass of water for easier oral consumption, as needed.<br><br>Prior to ^{124}I-PU-AD PET studies, patients will be permitted to eat a light meal. For the ^{124}I-PU-AD PET study, two IV catheters (heparin-locked) will be placed in the subject for radiopharmaceutical administration and for blood sampling.

	After ^{124}I-PU-AD tracer injection, PET scans will be performed at the following time-points: immediately post-injection; 2.5-4.5 (+/- 30 mins) hours, post-injection; and 1-2 days, post-injection. Optionally, in willing patients, an additional PET scan will be obtained 3-7 days post-injection.<br><br>At each time-point, a 30-60 minute axial body image (spanning from skull vertex to proximal thigh regions) is acquired on a state-of-the-art PET-CT scanner. A low-dose CT will be obtained immediately-prior to PET imaging, at each time-point. A 30-60 minute scanning time-period is typical for clinical nuclear medicine diagnostic imaging studies. Subjects will be monitored visually and communication will be maintained directly between the patient and the radiology investigators, except during the CT, when communication will be maintained via a speaker system. The radiology investigators will evaluate patients during the early time-period, post-injection.<br><br>Serial blood samples will be obtained at approximately 1, 5, 15±5, 30±5, 60-90 (±30) minutes; and 2.5-4.5 (±30 mins) hours, post-injection. Serial blood samples shall also be obtained, weighed, and whole-blood activity counted. Blood samples will then be centrifuged and the plasma pipetted, weighed and counted to determine the plasma time activity concentration curves (% injected dose/liter), as well as for metabolite analysis of the ^{124}I-PU-AD compound (by radio-HPLC and/or TCA methodology).<br><br>Between assays, the subject is free to engage in regular activities (ambulating, eating, drinking, toileting, etc.). During these breaks, the patient will be free to temporarily depart the clinic.<br><br>Subjects will be treated at the discretion of their physicians.<br><br>Subjects will be evaluated to ensure that there are no clinically significant ongoing AEs prior to discharge.
**Eligibility Criteria**	**<u>Inclusion Criteria:</u>**<br>1. Subject is between 21-90 years old at time of consent.<br>2. Subject has negative serum pregnancy test for females of childbearing age (11-50 years) and/or lack child-bearing potential.<br>3. Subjects with a diagnosis of cancer and/or Alzheimer's Disease, meeting trial eligibility criteria as specified below for either disease:<br>***<u>Cancer:</u>***<br>a. Subjects with eligible histologic types of cancer. Eligible histologic types of cancer include solid malignancy, myeloma, and lymphoma.<br>b. Cancer histology confirmed by pathology.<br>c. Cancerous disease is radiologically-measurable or evaluable as defined by published tumor response criteria (including but not limited to RECIST 1.1).<br><br>***<u>Alzheimer's:</u>***<br>a. Established diagnosis of mild-moderate Alzheimer's disease based upon neurological and neuropsychological evaluation following the National Institute on Aging – Alzheimer's disease Association criteria that recently revisited the NINCDS-ADRDA

	criteria. (14)<br>b. Documentation of diagnosis of mild-moderate Alzheimer's disease, as above, by board-certified neurologist.<br>c. Subject has an appointed health care proxy specifically designated for research consent and this appointment is documented.<br>d. Subject has designated at-home caregiver(s) responsible for providing daily medications to the patient, who will document the patient's daily doses of SSKI oral medication, for 2 weeks, as per study protocol.<br>4. Subjects who have both cancer and Alzheimer's Disease, subjects are considered eligible if they meet all eligibility requirements for either Alzheimer's Disease or cancer patients, as specified above.
	**<u>Exclusion Criteria:</u>**<br>1. Subject has history of allergic reaction to X-ray CT iodinated contrast medium.<br>2. Subject has hypersensitivity to iodide products.<br>3. Subject has known hyperthyroidism.<br>4. Subject has unacceptable pre-study organ function during screening defined as:<br>a. Bilirubin > 1.5 x institutional upper limit of normal (ULN)<br>b. AST/ALT >2.5 x ULN<br>c. Albumin < 2 g/dl<br>d. GGT > 2.5 x ULN (**<u>IF</u>** Alkaline phosphatase > 2.5 x ULN)<br>e. Creatinine >1.5 x ULN or creatinine clearance < 60 mL/min.<br>5. Subject has history of acute major illness (i.e., unstable cardiovascular condition.)<br>6. Subject has inability to give consent personally or via appointed health care proxy.<br>7. Women who are pregnant or breastfeeding (and for 1 month after receiving the study drug).<br>8. Subject has any other condition or laboratory abnormality, or receives any other treatment(s) that may increase the risk associated with study participation or may interfere with the interpretation of study results in the judgment of the investigator.<br>9. Subject has concurrent participation in any interventional studies within 30 days of first dose of study drug.
**Study Drug**	^{124}I-PU-AD is the non-radioactive PU-AD therapeutic drug which contains an endogenous iodine atom (the naturally-occurring stable isotope iodine-127).<br><br>^{124}I-PU-AD and PU-AD are the same molecule, in chemical structure. The non-radioactive PU-AD therapeutic drug contains an endogenous iodine atom (the naturally-occurring stable isotope iodine-127). ^{124}I-PU-AD has the same molecular structure as PU-AD, with a positron-emitting iodine-124 atom in place of iodine-127.<br><br>^{124}I-PU-AD will be supplied by Memorial Sloan Kettering Cancer Center (MSKCC). Please reference the pharmacy manual for additional details.

**Study Rationale**	The U.S. FDA has encouraged use of the microdose PET trial design as an opportunity to discover and address fundamental technical issues that may abort clinical drug development or encourage pursuit of more-promising avenues of research, avoiding a greater expenditure of resources.<br><br>Additionally, microdose PET data offers data suitable for hypothesis-generating. For example, the radiation dosimetry data to be obtained for ^{124}I-PU-AD can be directly-extrapolated to alternative iodine isotopes, including iodine-123-PU-AD, as a potential single-photon imaging agent.
**Statistical Methods**	**Primary Endpoints:**<br>• PK of ^{124}I-PU-AD in subjects with specific cancer types and/or Alzheimer's disease.<br>• Metabolism, biodistribution, and radiation dosimetry as assessed by the collection of imaging and blood data in this patient population.<br><br>**Secondary Endpoints:**<br>• Safety of ^{124}I-PU-AD in subjects as assessed by evaluation of adverse events (AEs) and serious adverse events (SAEs).<br><br>PET and blood data is used to quantify tracer-biodistribution, measuring the amount of radioactivity present in the blood and bodily regions-of-interest, at each time-point (expressedin terms of percentage injected-dose per unit tissue volume or mass).<br><br>**Pharmacokinetic and Metabolites Analyses**<br>Blood/serum analyses identify and quantify the presence of metabolites versus intact compound. The blood-based datasets shall be used to quantify the characterization of tracer-metabolism. This data shall be used to derive the quantitative PK parameters of tracer-uptake, -clearance and tracer-excretion. PK parameters of PU-AD and its metabolite (including, but not limited to C_{max}, T_{max}, $AUC_{0\text{-}24\ hr}$, AUC_{inf}, CL, and $t_{1/2}$) will be analyzed descriptively.<br><br>**Biodistribution Analyses**<br>^{124}I-PU-AD is injected as a single intravenous bolus. The patient then undergoes PET and blood assays, at multiple time-points; the PET and blood data is used to quantify tracer-biodistribution, measuring the amount of radioactivity present in the blood and bodily regions-of-interest, at each time-point (expressed, e.g., in terms of percentage injected-dose per unit tissue volume or mass). The biodistribution parameters of ^{124}I-PU-AD will be summarized using medians as well as graphically for each bodily region.<br><br>**Radiation Dosimetry Analyses**<br>The PK parameters are used to derive the radiation dosimetry parameters of absorbed-dose and effective-dose. The radiation dosimetry parameters will be summarized descriptively.<br><br>**Safety Analyses**<br>Evaluation of safety will be performed on the Safety Analysis Set. Safety data to be evaluated include AEs and SAEs. Note that AEs will be observed for all patients through Day 2, and thereafter will be collected from telephone interviews through Day 30.

	Toxicities will be assessed according to the National Cancer Institute CTC Scale (Version 4.0). Thyroid abnormalities observed at the 6-12 month assessment will be recorded. A baseline serum TSH will be obtained within 4 weeks prior to the ^{124}I-PU-AD injection. Thyroid ^{124}I uptakes doses will be measured.<br><br>AEs will be coded using MedDRA (Version 15.0 or higher). An overall summary of AEs will be provided, as well as summaries of treatment-emergent AEs (TEAE) by MedDRA system organ class (SOC) and preferred term (PT). TEAEs by SOC and PT will be provided overall, for treatment-related TEAEs, by maximal severity, and by study day of onset.<br><br>A formal Statistical Analysis Plan (SAP) will be finalized prior to database lock and will detail the methods of analysis and summarization for all of the study endpoints.
**Site Location**	Up to 4 investigative sites in the United States
**Duration of Participation**	Subjects will be participating in this study for up to approximately 13 months (4 week screening period, and up to 12 months of follow-up).

1. SCHEDULE OF ASSESSMENTS

Table 1: Study Procedures

	Screening (28 days)	Prior to Day 1 (2-24 hrs)	Day 1	Day 2	Optional Day (Day 3-7)	Day 30: Telephone Follow Up	6-12 Month Follow Up
Informed Consent	x						
Inclusion/Exclusion	x						
Concomitant Medications	x	x	x	x	x		
Adverse Events		x	x	x	x	x[i]	
Abbreviated Physical Exam[a]	x						
Serum Chemistries [b]	x						
Hematology (Complete Blood Count)[b]	x						
Serum thyroid stimulating hormone[c]	x						x
Pregnancy Test [d]	x						
Plasma PK draws[e]			x				
^{124}I-PU-AD Injection/ Radiopharmaceutical Administration			x				
Brain MRI[f]				x			
PET-CT Scans[g]			x	x	x		
SSKI drops[h]		x					

a Abbreviated Physical Exam includes Medical History.

b **Serum Chemistries:** Serum BUN, creatine, AST, ALT, total bilirubin, alkaline phosphatases, GGT (only IF alkaline phosphatase is > 2.5 x ULN) and albumin **Hematology:** complete blood count will be completed at the time of screening. Both serum chemistries and hematology should be completed within 2 weeks of study entry.

c Serum thyroid stimulating hormone (TSH) assay: TSH must be taken from the subject within 4 weeks prior to Day 1 to establish a baseline.

d Pregnancy Test: Serum pregnancy test for Women of Child Bearing Potential (i.e., premenopausal and not surgically sterile/ 11-50 years) will be performed at the screening visit per site standard.

e Plasma PK draws: Blood will be drawn at multiple time points: 1, 5, 15±5, 30 ±5 min, 60-90 (± 30) minutes, and 2.5-4.5 (± 30 mins) hours post-injection.

f The MRI may be waived if a prior scan meeting study requirements was completed within 2 months of study enrollment. The brain MRI will be completed 24-48 hours post-injection during the 1-2 days post-injection PET scan timepoint.

g PET-CT Scans": PET scans will be performed at the following time-points: Day 1: immediately/post-injection and 2.5-4.5 hours (+/- 30 mins) post-injection; and 1-2 days post-injection. Optionally, in willing patients, an additional PET scan will be obtained 3-7 days post-injection. At each time-point, a 30-60 minute axial body image (spanning from skull vertex to proximal thigh regions) is acquired on a state-of-the-art PET-CT scanner. A low-dose CT will be obtained immediately-prior to PET imaging, at each time-point. A 30-60 minute scanning time-period is typical for clinical nuclear medicine diagnostic imaging studies.

h SSKI drops: 7 drops of SSKI will be given to the subject orally, daily, for 2 weeks; the first oral dose occurring the day before or at least 2 hours prior to the administration of ^{124}I-PU-AD. For AD subjects, the subject's LAR will assume responsibility for ensuring adherence to the daily SSKI dosing regimen prescribed, documenting the time and date of each dose in a study medication journal. The subject will be contacted by a study assistant prior to ^{124}I-PU-AD administration and on the 2nd day of PET scanning to ensure compliance.

i Telephone Follow-up: On Day 30, a telephone call should be made in order to assess any AEs and changes in medications. A minimum of 3 attempts should be made and documented before documenting a subject as non-responsive.

TABLE OF CONTENTS

SPONSOR AUTHORIZATION 2
PROTOCOL SYNOPSIS 3
Pharmacokinetic and Metabolites Analyses 6
Biodistribution Analyses 6
Radiation Dosimetry Analyses 6
1. SCHEDULE OF ASSESSMENTS 8
2. INTRODUCTION 13
2.1. Mechanism Of Action 14
2.2. Study Rationale 15
2.2.1. Rationale for Alzheimer's Disease 16
2.2.2. Non-Clinical Experience with PU-AD 17
2.2.3. Rationale for Dose and Schedule Selection 18
3. OBJECTIVES AND ENDPOINTS 19
3.1. Objectives 19
3.2. Endpoints 19
4. STUDY DESIGN 20
4.1. Overall Design 20
5. STUDY POPULATION 21
5.1. Eligibility Criteria 21
5.1.1. Inclusion Criteria 21
5.1.2. Exclusion Criteria 21
6. TREATMENT 22
6.1. Study Drug 22
6.1.1. ^{124}I-PU-AD 22
6.2. PU-AD Administration 22
6.3. Treatment Blinding 23
6.4. Prior and Concomitant Medication 23
6.4.1. Prior Medication 23
6.4.2. Concomitant Medication 23

6.4.3. Prohibited Concomitant Medications 23
7. STUDY SUPPLY 23
7.1. ^{124}I-PU-AD Supply, Package and Labeling 23
7.2. ^{124}I-PU-AD Storage and Preparation 24
7.3. Accountability 24
8. STUDY CONDUCT 24
8.1. Subject Identification, Randomization and Enrollment 24
8.2. Discontinuation, Withdrawal and Replacement of Subjects 24
8.2.1. Discontinuation 24
8.2.2. Withdrawal from the Study 25
8.2.3. Study Termination 25
9. STUDY ASSESSMENTS AND PROCEDURES 26
9.1. Informed Consent 26
9.2. Pharmacokinetic Measurements 26
9.3. Medical Imaging 26
9.3.1. PET-CT scan 26
9.3.2. Brain MRI 26
9.4. Safety Assessments 27
9.4.1. Pregnancy Test 27
9.4.2. Physical Examinations 27
9.4.3. Protocol-Required Safety Laboratory Assessments 27
9.4.4. Telephone Contact 27
9.5. Adverse Events and Serious Adverse Events 27
9.5.1. Definitions 27
9.5.1.1. Adverse Event 27
9.5.1.2. Serious Adverse Event 28
9.5.2. Adverse Events Assessment 29
9.5.2.1. Severity 29
9.5.2.2. Relationship to Study Drug 30
9.5.2.3. Causality 30

9.5.2.4. Adverse Event Outcome 31
9.5.3. Recording and Reporting of AE and SAE Information 31
9.5.3.1. Recording of Adverse Events 31
9.5.3.2. Reporting of Serious Adverse Events 31
9.5.3.3. Follow-up of AEs and SAEs 32
9.5.3.4. Regulatory Reporting Requirements for SAEs 32
9.5.4. Pregnancy 32
10. STATISTICAL CONSIDERATIONS 33
10.1. Sample Size Determination 33
10.2. Populations for Analyses 33
10.3. Statistical Analyses 33
10.3.1. Primary Analyses 33
10.3.1.1. Pharmacokinetic and Metabolites Analyses 34
10.3.1.2. Biodistribution Analyses 34
10.3.1.3. Radiation Dosimetry Analyses 34
10.3.2. Secondary Analyses 34
10.3.2.1. Safety 34
10.3.3. Other Analyses 34
11. REGULATORY AND ETHICAL CONSIDERATIONS 35
11.1 Good Clinical Practice 35
11.2 Informed Consent Process 35
11.3 Institutional Review Board 35
11.4 Publication Policy 36
12. STUDY MANAGEMENT 36
12.1. Data Quality Assurance 36
12.2. Case Report Forms and Source Documentation 36
12.3. Monitoring 37
12.4. Inspections 37
12.5. Financial Disclosure Reporting Obligations 37
12.6. Archiving Study Records 38

13. REFERENCES 39

14. APPENDICES 41

Appendix 1: Abbreviations and Trademarks 41

Appendix 2: Contraceptive Guidance & Collection of Pregnancy Information ..43

Appendix 3: Investigator Statement 44

LIST OF TABLES

Table 1: Study Procedures 8

Table 2: Classifications for AE Causality 30

Table 3: Classifications for Outcome of an AE 31

2. INTRODUCTION

Samus Therapeutics, Inc. (Samus) is developing two purine-based epichaperome inhibitors discovered at Memorial Sloan Kettering Cancer Center (MSKCC), PU-H71 for cancer and PU-AD for cancer and neurodegenerative diseases. Both drug candidates have high affinity and specificity for the epichaperome specific HSP90 in diseased cells, but do not bind to, nor affect HSP90 in normal cells. PU-H71 has limited blood-brain barrier permeability and thus is not an ideal agent for the treatment of central nervous system diseases. PU-AD is structurally, chemically and biochemically very similar to PU-H71 but contains minor modifications to enable for blood brain barrier permeability. In addition, and like PU-H71, PU-AD contains endogenous iodine, enabling its labeling with PET-amenable iodine, ^{124}I. While PU-AD may be used for the imaging and treatment of cancers, and as a single/combination agent in the treatment of Alzheimer's disease.

The 'chaperome' is a term used to refer to several families of chaperones, co-chaperones, foldases, isomerases, and scaffolding proteins that together with the protein degradation machineries regulates proteostasis in a cell.[8]. Epichaperome is a term used for chaperome networks formed under cellular stress such as in cancer, neurodegenerative and other diseases. The heat shock proteins (HSPs) – HSP60s, HSP70s, HSP90s, HSP110s, and the small HSPs are some of the many chaperones within the chaperome.[18] The most common molecular chaperone in human cells is HSP90. HSP90 regulates the stability and activation of what is known as "client proteins".[1] HSP90, like other members of the HSP family, is necessary for essential cellular housekeeping functions, though its levels are dramatically increased in response to stressors such as heat or lack of oxygen.[2] HSP90 is comprised of three functional domains: an NH_2-terminal ATP/ADP-binding domain, a client protein-binding middle domain, and a COOH-terminal dimerization domain.[1] More than 100 HSP90 client proteins have been identified; mainly signal transduction proteins such as kinases and transcription factors. Activation of signaling pathways mediated by these client proteins is necessary for the regulation of cell cycle progression, cell proliferation, and apoptosis. [3]

Chaperones have long been associated with the management of cellular stress, including neuronal stress. They are induced after an acute stressful event, such as heat, thus deriving their name of heat shock proteins. These effects are rapid in onset. In fact, changes in chaperone expression and the formation of epichaperomes have long been believed to be at the core of many diseases, including cancer and neurodegenerative diseases.

In comparison to individual chaperomes, the epichaperome is better suited to deal with the increased proteome demand induced by chronic stress. Epichaperomes function to preferentially associate with proteins and protein networks that maintain and drive the pathologic cellular phenotype. Through these interactions epichaperome networks propagate alterations in the proteome that are associated with and promoting of disease. This mechanism therefore represents a novel usage of the chaperome members in regulation of chronic stress such as in disease, where the cell responds to chronic stress not by an upregulation of the heat shock proteins and of other chaperones, but rather by the incorporation of individual chaperome units into the formation of epichaperomes.

It has been hypothesized that chronic neuronal stresses may also cause a re-sculpting of the chaperome machinery and its function, in a fashion similar to that seen in cancer. Specifically, it is thought that neuronal adaptation effects to the chaperome may become maladaptive when

molecular stress occurs chronically, such as in neurodegenerative diseases, and that chaperome re-sculpting into epichaperomes may be a potential mechanism by which the toxic proteome is propagated and promoted.

PU-AD has high specificity for epichaperomes but does not inhibit housekeeping HSP90 in chaperomes that maintain normal homeostasis. Specific targeting of epichaperome complexes containing HSP90 is possible due to its structural and biochemical nature which is distinct from that of housekeeping HSP90 in chaperomes. Epichaperomes are present in cells in multiple diseases including cancer and neurological disorders. Their role in maintaining cellular survival in diseases is dictated by the client proteins such as oncoproteins in cancer or mutated tau (and possibly other abnormal proteins) in some neurodegenerative diseases. Therefore, targeting the epichaperome in cancer results in cell death, and, in neurodegenerative diseases, neuronal survival, and most importantly, with no observed effects on normal cells.

2.1. Mechanism Of Action

PU-AD is a potent inhibitor of stress HSP90 as found in tumors and in other pathologies, such as in neurons undergoing a neurodegenerative transformation.[(9)] For example it binds stress HSP90 from MDA-MB-468 cancer cells and from the JNPL3 Alzheimer's brain derived (JNPL3 is a tau transgenic mouse model) with an affinity (EC_{50}) of 6.9 nM and 5 nM, respectively.

In cancer cells, the effects of PU-AD are seen at concentrations comparable to its observed EC_{50} for stress HSP90. An accepted measure for in cell inhibition of HSP90 is the analysis of aberrant proteins dependent on it for stability; when the function of HSP90 becomes impaired, client proteins become targeted for degradation by the proteasome. Along these lines, PU- AD degraded HER2 in the HER2-overexpressing cancer cell SKBR3 with a recorded IC_{50} of 8.2 nM.

In contrast, and as a testament to its specificity for stress HSP90, concentrations as high as 100 µM (one hundred micromolar; 2000 fold higher than its affinity for stress HSP90) and a constant exposure of 72 hours were needed to induce toxicity in normal neurons.

PU-AD follows the in vivo profile of PU-H71 with the exception that, unlike PU-H71, it optimally permeates the brain. To summarize, PU-AD rapidly distributes to tissues but also rapidly clears from plasma and normal tissues. In contrast, PU-AD demonstrates a slow clearance from stress HSP90 expressing tumors (and other pathologic tissues, such as brain regions afflicted by neurodegeneration). This unique behavior of PU-AD provides the rationale for its favorable therapeutic index. It also provides the rationale for the use of a ^{124}I- labeled PU-AD as a PET imaging agent to detect tissues associated with stress HSP90 and to inform on the biodistribution and PK of PU-AD.

Refer to Investigator's Brochure for additional clinical details.

2.2. Study Rationale

The trial design accomplishes the standard first step for introducing a new radiolabeled drug, into human usage: testing the safety and in vivo behavior of the compound, in a pilot study.[1,2] This includes first-in-human data on the radiation dosimetry, metabolism, biodistribution, PK, and toxicity of ^{124}I-PU-AD at trace (microgram and millicurie) doses. The Investigators expect this pilot trial will provide the prerequisite human radiation dosimetry and PK data to justify clinical development of ^{124}I-PU-AD as an agent for PET imaging. The potential clinical applications of ^{124}I-PU-AD PET imaging are multiple, as shall be discussed.

The primary objective of the proposed pilot study is to obtain first in-human data on the on the PK, metabolism, biodistribution and radiation dosimetry of ^{124}I-PU-AD as a prerequisite step in the clinical development of ^{124}I-PU-AD as an imaging agent, as well as to inform parallel clinical development of the non-radioactive therapeutic compound PU-AD. ^{124}I-PU-AD and PU-AD are the same molecule, in chemical structure. The non-radioactive PU-AD therapeutic drug contains an endogenous iodine atom (the naturally-occurring stable isotope iodine-127). ^{124}I-PU-AD has the same molecular structure as PU-AD, with a positron-emitting iodine-124 atom in place of iodine-127. Hence, ^{124}I-PU-AD is perfectly-suited for use as a 'tracer' of PU-AD. Therefore, this pilot PET trial also obtains basic information applicable to the first-in-human evaluation of the therapeutic PU-AD compound as a 'microdose' study.[2] The ^{124}I-PU-AD PK data obtained in this first-in-human trial will be of immediate value to the Investigators for the design of a planned separate phase 1 PU-AD therapy trial.

The U.S. FDA has encouraged use of the microdose PET trial design as an opportunity to discover & address fundamental technical issues that may abort clinical drug development or encourage pursuit of more-promising avenues of research, avoiding a greater expenditure of resources. Additionally, microdose PET data offers data suitable for hypothesis-generating. For example, the radiation dosimetry data to be obtained for ^{124}I-PU-AD can be directly extrapolated to alternative iodine isotopes, including iodine-123-PU-AD, as a potential single-photon imaging agent.

Targeted imaging for targeted therapy —using radiolabeled forms of targeted therapeutic agents for PET imaging — is much advocated for modern drug development, by the National Cancer Institute and others.[3-6] The unique potential of PET microdose studies in development of drugs as therapeutic and/or diagnostic imaging agents is recognized by the U.S. FDA and others. An excellent review of published PET micro-dosing studies is provided by Wagner et al.[7]

PET-based microdose studies offer data that conventional plasma-based microdose studies cannot: notably, PET imaging of drug biodistribution, including tumor-targets and non-target tissues/organs. The Study Investigators have designed the trial to obtain important first in-human data to help them better-decide:

1. How to use PU-AD therapy, in its first phase 1 trial. This PET study will provide first in-human microdose data on PU-AD PK, metabolism, and biodistribution. This information will be of key value, aiding rational design of the phase 1 PU-AD therapy trials.

2. What directions should be taken for the next ^{124}I-PU-AD PET clinical trial? The data on PK, metabolism, and biodistribution (including radiation dosimetry) will determine if ^{124}I-PU-AD is a viable candidate for further development as a PET imaging drug.

In vivo PET imaging of ^{124}I-PU-AD in tumor-bearing mice provided visualization of intracranial tumors and differentiated HSP90-addicted tumors form those impervious to HSP90 therapy. Specific tumor uptake and retention of ^{124}I-PU-AD PET was confirmed by autoradiography, H&E staining and PU-AD PET imaging. Because of the specific interaction of PU-AD with the "stress HSP90" the method described here may provide a means for the non-invasive detection of human brain pathologies associated with "stress HSP90". Thus PU-AD PET may provide an answer to the question "what patients are more likely to benefit from PU-AD?". PU-AD PET is also a companion diagnostic for PU-AD; it may provide answers to important questions in drug development: "what dose of PU-AD should be administered for optimal target engagement?" "How often should PU-AD be administered for optimal target engagement?" "At what dose of PU-AD are systemic toxicities possible in an individual patient?"

To conclude, the introduction of non-invasive diagnostic tools, such as the one presented here for stress HSP90, will likely spur more research in this area and ultimately move the field towards an era when such methods will become common practice in oncology. Ultimately, the approaches outlined hold out the promise of accurately guiding clinical decision-making on an individualized basis, fulfilling a quest of modern oncology.

2.2.1. Rationale for Alzheimer's Disease

Clinical biomarker research on behalf of the Alzheimer's Disease (AD) patient community addresses an urgent, unmet need of paramount importance in the aging U.S. population. Development of objective biomarker assays is essential to the yet-unrealized clinical goal of developing an effective treatment for AD. In particular, current therapeutic efforts are focusing upon patients with preclinical and early-stage AD – but current neuroradiologic imaging cannot accurately diagnose or evaluate preclinical or early-stage AD.[(12)]

MSKCC has developed ^{124}I-PU-AD, a novel epichaperome-targeted small molecule radiotracer for detection and evaluation of diseases in which overactivity of the epichaperome is key, notably including cancer and neurodegenerative diseases. [(18)] As discussed in the trial protocol and herein, the epichaperome is implicated the onset and progression of AD from its earliest stages. [(13)]

Clinical development of novel AD assays necessitates the study of AD patients in a safe manner. A small pilot study of patients with mild/moderate AD may provide a safe, logical first step in clinical development of ^{124}I-PU-AD PET: the patient with mild/moderate AD is fully-able to comply with the minimal, brief requirements of ^{124}I-PU-AD PET.

- Mild/moderate AD patients are emotionally and intellectually stable.
- Mild/moderate AD patients retain self-identity and recognize family and personal care-givers.
- Mild/moderate AD patients possess normal verbal communication ability and can follow verbal instructions.

Minimal, brief requirements of ^{124}I-PU-AD PET will be performed using a standard PET scanning approach. The AD patient rests supine on a scanner bed (with no use of restraints of the head or any other kind) with a legally authorized representative (LAR) present at all times; the patients are able to converse during each scan (duration of 30 minutes each). Standard clinical practice demonstrates the ease with which the mild/moderate AD population tolerates diagnostic neuroradiologic exams of similar duration, in enclosed scanners: MRI, F-18 fluorobetapir PET, F-18 FDG PET, C-11 PIB PET.

The PET scan can be terminated at any time, if the patient and/or his/her LAR so chooses. The patient's LAR and a research team member will be beside the patient, at all times, reassuring the patient and checking his/her comfort at regular intervals. The PET scanner contains an internal microphone so that the patient can be heard by the research team at all times.

The main objective of the clinical trial is a first in-human evaluation of tracer PK. Human subject participation in the PET trial is most-justified when the Investigators extract the maximal scientific information possible, from the first in-human data gathered from trial participants. Exploring the anticipated clinical applications for which ^{124}I-PU-AD PET is being developed – namely tumor imaging and AD imaging – are safe, logical exploratory objectives in the first in-human trial.

Clinical development of novel AD assays necessitates the study of AD patients in a safe manner. The mild/moderate AD patient population, in the Investigators' collective experience and by standard clinical practice with similar neuroradiologic exams, can easily tolerate the ^{124}I-PU-AD PET scan.

This study provides an appropriate first opportunity for exploring differences in ^{124}I-PU-AD brain uptake between patients with an established AD diagnosis versus patients with no known cognitive/CNS disorder. Gathering 'hypothesis-generating data' is recognized by the US FDA and others as a desirable goal in first in-human trials of novel radiotracers. The Investigators expect to observe distinct differences in the brain uptake of AD patients versus patients without cognitive dysfunction/CNS disease, as discussed below. Obtaining supportive preliminary data of this kind from a pilot study provides a standard first-step in clinical development of a novel radiotracer. Such preliminary data, from a small number of patients, can prove a major catalyst to clinical development of a novel radiotracer; notably, in the Investigators' collective experience, such preliminary data in grant applications can have a powerful positive impact upon funding decisions, and presentation of preliminary data in manuscripts and society meetings attracts academic and commercial collaborations. Of equal importance and anticipated positive impact, the non-radioactive form of the PU-AD molecule (identical in molecular structure to ^{124}I-PU-AD but with a stable iodine isotope) is under translational development as a novel therapeutic for AD and neurodegenerative disease. First in-human data on the microdose in vivo PK of ^{124}I-PU-AD will be invaluable to ongoing design of the first in-human PU-AD therapy trial; in particular, the PET trial data will inform rational selection of PU-AD therapeutic dose and schedule based upon the uptake and duration of retention of PU-AD-tracer in normal versus diseased tissues.

2.2.2. Non-Clinical Experience with PU-AD

To develop safer HSP90 inhibitors, MSKCC was the first to develop non-quinone containing, purine-based, ATP-competitive inhibitors of HSP90. These were designed to bind with higher specificity and affinity to the stress HSP90 over the normal cell housekeeping HSP90.

PU-AD has a favorable PK and PD profile, with target engaging concentrations being delivered to mice at non-toxic doses. PU-AD follows the in vivo profile of PU-H71 with the exception that, unlike PU-H71, it optimally permeates the brain. To summarize, PU-AD rapidly distributes to tissues but also rapidly clears from plasma and normal tissues. In contrast, PU-AD demonstrates a slow clearance from stress HSP90 expressing tumors (and

other pathologic tissues, such as brain regions afflicted by neurodegeneration).This unique behavior of PU-AD provides the rationale for its favorable therapeutic index. It also provides the rationale for the use of a ^{124}I-labeled PU-AD as a PET imaging agent to 1. detect tissues associated with stress HSP90 and 2. inform on the biodistribution and PK of PU-AD.

To assess acute toxicity of a single intravenous (IV) infusion of PU-AD, B6D2F1 mice (15 males and 15 females/group) were dosed IV once with either vehicle control or PU-AD at a dose of 2 mg/kg, which corresponds to 200x the maximum intended human dose for the PU-AD PET study. Ten mice /sex/group were sacrificed 24 hours after test article administration while the remaining mice were kept under observation for an additional 13 days before final sacrifice. Gross and complete necropsy was conducted on all mice and clinical chemistry and hematology analyses were performed. No mortality or morbidity was observed during the treatment or the observation period; in general, all mice looked healthy and displayed a normal behavior throughout. No significant weight loss was evident in any of the groups and hematology and clinical chemistry parameters were all within the norm. No abnormal findings were detected during gross necropsy, while complete histopathology evaluation is still pending. A complete safety toxicology report on this study will be included in the IND application.

To asses if chronic administration of PU-AD presented any toxicities, a preliminary study in 13 (6 male & 7 female) B6C3F1 mice was conducted. Mice were dosed IV 3 times per week for approximately 24 weeks with either vehicle control or PU-AD at a dose of 75 mg/kg. No mortality or morbidity was observed during the treatment or the observation period. All mice were sacrificed 24 hours after final test article administration. Complete necropsies and analyses of hematology and clinical chemistry were conducted on all mice. No significant weight loss was evident in any of the groups. All hematological or clinical chemistry findings were within normal parameters; no negative statistically significant differences were observed between vehicle and the test article. Histopathology was conducted in major organs to show no toxic changes induced by PU-AD (see below on select organs linked to tox findings with other HSP90 agents). Thyroid was evaluated because PU-AD contains an iodine.

2.2.3. Rationale for Dose and Schedule Selection

PU-AD will be prepared according to the pharmacy manual. ^{124}I-PU-AD is a radiolabeled form of PU-AD which contains stable iodine in the same position on the molecule, making ^{124}I-PU-AD a true tracer for the drug.

The predicted *effective dose* for humans, from a maximal 10 mCi of ^{124}I-PU-AD, based upon data extrapolated for animal studies, is 0.3 rem. This effective dose is similar to that received from the standard 12 mCi of ^{18}F-FDG administered to about 60 clinical patients a day at MSKCC. No patient has suffered an adverse event at MSKCC due to administration of these low effective doses of radiation. Expected dosimetry for this tracer is based on murine imaging studies. Allowing for considerations in isotope production, the maximal administered dose will be 10.0 mCi (range: 1.0-10.0 mCi), containing <100 µg of PU-AD.

^{124}I-PU-AD radiation dosimetry is a key part of this pilot trial, and ^{124}I-PU-AD radiation dosimetry analyses shall be ongoing during the trial. In brief, region-of-interest (ROI) image analysis of ^{124}I-PU-AD PET data allows organ dosimetry, calculated using the MIRD formalism as

implemented in the FDA-approved “OLINDA” software from Vanderbilt University. The quantity of ^{124}I is nontherapeutic and does not pose a radiation or health hazard to the patient, Center staff or the general public.

The PET/CT scanner acquires a low-dose CT image for anatomic localization of radioactive signals and attenuation-correction, for accurate quantification of tissue tracer-concentrations. The radiation dose is scaled to the body weight of the patient. The maximum absorbed radiation dose, from the low-dose CT, based on phantom measurements, is approximately 526 millirems (mrem). This trial involves 3 low-dose CT scans, per patient, for a total CT dose of ~ 1.6 rem, per patient. For patients who participate in the optional PET-CT scans, the total CT dose is ~ 2.1 mrem. This effective dose, from the low-dose CT scans, is comparable to the effective dose delivered by a single standard-dose axial torso CT (2.8 rem) or the effective dose from <2 helical torso CT scans (1.5 rem per helical torso CT).(11) The expected combined effective dose from the maximal four low dose CT scans plus a maximal 10 mCi tracer dose is projected to be 2.5 rem, less than the combined effective dose from two standard torso CT scans. The companion CT of the PET study is used for two key purposes: attenuation-correction & anatomic-localization of PET data. CT-based attenuation-correction is important for improving the accuracy of PET-based measurements of tissue tracer-levels. CT-based anatomic localization of in vivo tracer-uptake is key for accurate determination of tracer-biodistribution.

Refer to Investigator’s Brochure for additional clinical details.

3. OBJECTIVES AND ENDPOINTS

3.1. Objectives

Primary Objectives:

- Determine the PK, metabolism, biodistribution, and radiation dosimetry of ^{124}I-PU-AD in subjects with specific cancer types (solid malignancy, lymphoma, and/or myeloma) and/or Alzheimer’s Disease.

Secondary Objectives:

- Determine the safety of ^{124}I-PU-AD in this population, as determined through recording of adverse events.

3.2. Endpoints

Primary Endpoints:

- PK of ^{124}I-PU-AD in subjects with specific cancer types and/or Alzheimer’s disease.
- Metabolism, biodistribution, and radiation dosimetry as assessed by the collection of imaging and blood data in this patient population.

Secondary Endpoints:

- Safety of ^{124}I-PU-AD in subjects as assessed by the evaluation of AEs and SAEs.

4. STUDY DESIGN

4.1. Overall Design

This first in-human trial of the positron-emitting agent ^{124}I-PU-AD is an open-label pilot study. Up to 10 evaluable subjects who have active disease will be enrolled to evaluate the PK, metabolism, biodistribution, and radiation dosimetry of ^{124}I-PU-AD.

Following a 28-day screening period as outlined in Table 1, eligible subjects will return to the clinic on Day 1. A single dose of ^{124}I-PU-AD will be administered by IV injection to subjects. The formulation includes a microgram PU-AD dose of up to 10.0 mCi (minimal toxicity risk) with a predicted radiation exposure comparable to that of standard radiology exams. Subjects are prescribed a standard clinical oral iodine regimen to reduce thyroid exposure. Seven drops of saturating solution of potassium iodide (SSKI) will be given to the patient orally, daily, for two weeks; the first oral dose occurring the day before or at least 2 hours prior to the administration of ^{124}I-PU-AD.

For Alzheimer's Disease patients, the patient's individual LAR will assume responsibility for ensuring adherence to the daily SSKI dosing regimen prescribed, documenting the time and date of each ingested dose of SSKI in a study medication journal. The site staff will contact the responsible caregiver prior to ^{124}I-PU-AD tracer administration and on the 2nd day of ^{124}I-PU-AD PET scanning to ensure SSKI compliance. The prescribed daily dose of 7 SSKI droplets can be added to a glass of water for easier oral consumption, as needed.

Prior to ^{124}I-PU-AD PET studies, patients will be permitted to eat a light meal. For the ^{124}I-PU-AD PET study, two IV catheters (heparin-locked) will be placed in the subject for radiopharmaceutical administration and for blood sampling.
After ^{124}I-PU-AD tracer injection, PET scans will be performed at the following time-points: immediately post-injection; 2.5-4.5 (+/- 30 mins) hours, post-injection; and 1-2 days, post-injection. Optionally, in willing patients, and additional PET scan will be obtained 3-7 days post-injection. At each time-point, a 30-60 minute axial body image (spanning from skull vertex to proximal thigh regions) is acquired on a state-of-the-art PET-CT scanner. A low-dose CT will be obtained immediately-prior to PET imaging, at each time-point. A 30-60 minute scanning time-period is typical for clinical nuclear medicine diagnostic imaging studies. Subjects will be monitored visually and communication will be maintained directly between the patient and the radiology investigators, except during the CT, when communication will be maintained via a speaker system. The radiology investigators will evaluate patients during the early time-period, post-injection.

Serial blood samples will be obtained at approximately 1, 5, 15±5, 30±5, 60-90 (±30) minutes; and 2.5-4.5 (±30 mins) hours, post-injection. Serial blood samples shall also be obtained, weighed, and whole-blood activity counted. Blood samples will then be centrifuged and the plasma pipetted, weighed and counted to determine the plasma time activity concentration curves (% injected dose/liter), as well as for metabolite analysis of the ^{124}I-PU-AD compound (by radio-HPLC and/or TCA methodology). Free radioiodine in plasma specimens will be readily identifiable, in radio-HPLC analyses, by its characteristic elution time.
Between assays, the subject is free to engage in regular activities (ambulating, eating, drinking, toileting, etc.). During these breaks, the patient will be free to temporarily depart the clinic.

Subjects will be treated at the discretion of their physicians. Subjects will be evaluated to ensure that there are no clinically significant ongoing AEs prior to discharge

5. STUDY POPULATION

5.1. Eligibility Criteria

Subjects must meet all entry criteria prior to enrollment. Prospective approval of protocol deviations related to criteria, also known as protocol waivers or exemptions, are not permitted.

5.1.1. Inclusion Criteria

1. Subject is between 21-90 years old at time of consent..
2. Subject has negative serum pregnancy test for females of childbearing age (11-50 years) and/or lack child-bearing potential.
3. Subjects with a diagnosis of cancer and/or Alzheimer's Disease, meeting trial eligibility criteria as specified below for either disease.

   For subjects with cancer:
   a) Subjects with eligible histologic type of cancer. Eligible histologic types of cancer include solid malignancy, myeloma, and lymphoma.
   b) Cancer histology confirmed by pathology.
   c) Cancerous disease is radiologically-measurable or evaluable as defined by published tumor response criteria (including, but not limited to RECIST 1.1)

   For subjects with Alzheimer's Disease:
   d) Established diagnosis of mild-moderate Alzheimer's disease based upon neurological and neuropsychological evaluation following the National Institute on Aging – Alzheimer's disease Association criteria that recently revisited the NINCDS-ADRDA criteria.(14)
   e) Documentation of diagnosis of mild-moderate Alzheimer's disease, as above, by board-certified neurologist.
   f) Subject has an appointed health care proxy specifically designated for research consent and this appointment is documented.
   g) Subject has designated at-home caregiver(s) responsible for providing daily medication to the patient, who will document the patient's daily doses of SSKI oral medication, for 2 weeks as per study protocol.

4. Subjects who have both cancer and Alzheimer's Disease, subjects are considered eligible if they meet all eligibility requirements for either Alzheimer's Disease or cancer patients, as specified above.

5.1.2. Exclusion Criteria

1. Subject has history of allergic reaction to X-ray CT iodinated contrast medium.
2. Subject has hypersensitivity to iodide products.
3. Subject has known hyperthyroidism.
4. Subject has unacceptable pre-study organ function during screening defined as:
   a. Bilirubin > 1.5 x institutional upper limit of normal (ULN)
   b. AST/ALT >2.5 x ULN

   c. Albumin < 2 g/dl
   d. GGT > 2.5 x ULN (**IF** Alkaline phosphatase > 2.5 x ULN).
   e. Creatinine >1.5 x ULN or creatinine clearance < 60 mL/min.
5. Subject has history of acute major illness (i.e., unstable cardiovascular condition.)
6. Subject has inability to give consent personally or via appointed health care proxy.
7. Women who are pregnant or breastfeeding (and for 1 month after receiving the study drug).
8. Subject has any other condition or laboratory abnormality, or receives any other treatment(s) that may increase the risk associated with study participation or may interfere with the interpretation of study results in the judgment of the investigator.
9. Subject has concurrent participation in any interventional studies within 30 days of first dose of study drug.

6. TREATMENT

6.1. Study Drug

6.1.1. ^{124}I-PU-AD

^{124}I-PU-AD is the non-radioactive PU-AD therapeutic drug which contains an endogenous iodine atom (the naturally-occurring stable isotope iodine-127).

^{124}I-PU-AD and PU-AD are the same molecule, in chemical structure. The non-radioactive PU-AD therapeutic drug contains an endogenous iodine atom (the naturally-occurring stable isotope iodine-127). ^{124}I-PU-AD has the same molecular structure as PU-AD, with a positron-emitting iodine-124 atom in place of iodine-127.

^{124}I-PU-AD is a radiolabeled form of PU-AD which contains stable iodine in the same position on the molecule, making ^{124}I-PU-AD a true tracer for the drug. The projected ^{124}I-PU-AD has a radiotracer specific activity of >60 mCi per µmole or about <10µg of PU-AD per mCi of ^{124}I. Once prepared by the site, the final product will be evaluated to ensure that it passes the radiochemical purity specifications.

^{124}I-PU-AD will be supplied by MSKCC. Please reference the pharmacy manual for additional details.

6.2. PU-AD Administration

The final ^{124}I-PU-AD product is formulated in <5% ethanol and 0.9% saline in a volume of 5-10 mL. ^{124}I-PU-AD will be administered by IV injection over approximately 1 minute on Day 1. A dose up to 10.0 mCi (range: 1.0-10.0 mCi) ^{124}I-PU-AD will be administrered intravenously with the patient at rest.

The injection rate will be slowed further, if needed, based upon patient feedback. At the discretion of the study investigator, the ^{124}I-PU-AD formulation will be diluted with normal saline (0.9%) to a total volume of 40 cc and infused over a 20-40 minute period using a standard syringe pump device, slowing or pausing the infusion, as needed, based upon patient feedback.

Please refer to the pharmacy manual for more details.

6.3. Treatment Blinding

Not applicable; this is an open-label study.

6.4. Prior and Concomitant Medication

6.4.1. Prior Medication

Any medication (including over-the-counter medications) or therapy administered to the subject during the 30 days prior to consent will be recorded on the appropriate Case Report Form (CRF) page.

6.4.2. Concomitant Medication

Any medication (including over-the-counter medications) or therapy administered to the subject from the time of consent through the Optional Day 3-7 visit will be recorded on the appropriate CRF page. If the subject does not complete the Optional Day 3-7 visit, concomitant medications will be collected through Day 2.

6.4.3. Prohibited Concomitant Medications

The Investigators feel that participation in the ^{124}I-PU-AD PET trial is unlikely to interfere with ongoing therapy or regimens. Receipt of the microdose ^{124}I-PU-AD radiotracer, because of the tiny mass-amount received (<100 micrograms), is felt to pose no reasonable risk of direct interaction or secondary interference with other medications or the metabolism of other medications. The SSKI oral supplement has no contraindications or precautions regarding drug-drug interactions with AD medications.

7. STUDY SUPPLY

Samus or its designee will supply study drug to the pharmacies at all participating study centers.

Please reference the Pharmacy Manual for additional detail regarding study drug.

7.1. ^{124}I-PU-AD Supply, Package and Labeling

^{124}I-PU-AD clinical supplies (study drug) are supplied by MSKCC and the MSKCC nuclear medicine department.

^{124}I-PU-AD labels will not bear any statement that is false or misleading in any manner or represents that the study drug is safe or effective for the purposes for which it is being investigated. The content of the labeling will be in accordance with local regulatory specifications and requirements.

7.2. ^{124}I-PU-AD Storage and Preparation

All ^{124}I-PU-AD is to be stored in a locked area, accessible only to appropriate study personnel.

^{124}I-PU-AD will be stored and prepared in accordance with the MSKCC nuclear medicine SOPs.

7.3. Accountability

The Investigator or delegate will maintain accurate records of receipt and condition of study drug, including dates of receipt. In addition, accurate records will be kept of the date and time administered, quantity administered and the subject to whom study drug was administered. Any reasons for departure from the specified dispensing regimen must also be recorded.

A Clinical Research Associate (CRA) will review study drug accountability records during routine monitoring visits. At the completion of the study, there will be a final reconciliation of all study drug.

8. STUDY CONDUCT

8.1. Subject Identification, Randomization and Enrollment

All subjects screened for the study will be assigned a unique 5 digit identification (ID) number, including a 2-digit study center number and 3-digit subject ID number, starting with 001. The ID number will be used to distinguish subjects throughout their participation in the study and must be used on all study documentation related to that subject. Subject ID numbers will be assigned sequentially at each center.

Consented subjects who satisfy all entry criteria may be enrolled into the study. Consented subjects who fail to qualify for the study based on screening assessments are considered screen failures. Data corresponding to screen failures will NOT be recorded in the CRF. Screen Failure data will be maintained in the site's study file and the reason for the screen failure will be documented on the site screening log.

Randomization is not applicable to this study.

Subjects may only be rescreened if permission is granted by the medical monitor; however, ID numbers will not be re-used if a subject is a screened failure.

8.2. Discontinuation, Withdrawal and Replacement of Subjects

8.2.1. Discontinuation

The Investigator or Sponsor may discontinue study treatment for a given subject at any time. Reasons to discontinue study treatment will include, but not be limited to:

- Withdrawal of consent by the subject or refusal by the subject to continue treatment and/or study procedures.

- Subject experiences an unacceptable SAE/AE.
- Subject becomes pregnant.
- The Investigator feels it is in the subject's best interest to withdraw from the study.
- Subject fails to comply with protocol requirements
- Subject cannot tolerate/comply with the planned studies
- Technical problem with the PET cameras and imaging cannot be performed
- If the subject's primary physician and PI consider that further participation in the protocol would not be in the best interest of the patient
- If at any time the patient is found to be ineligible for the protocol as designated in the section on Eligibility Criteria, the subject will be removed from the study.
- Other reasons including protocol violations or non-compliance.

The reason for termination, date of ^{124}I-PU-AD dose, must be recorded on the Case Report Form (CRF) and source documents.

If the subject discontinues regardless of the reason, the final evaluations are to be performed as completely as possible.

8.2.2. Withdrawal from the Study

A subject may withdraw from the study at any time at his/her own request, or may be withdrawn at any time at the discretion of the investigator, Sponsor for safety, behavioral, compliance, or administrative reasons.

If the subject withdraws consent for disclosure of future information, the Sponsor may retain and continue to use any data collected before such a withdrawal of consent. If a subject withdraws from the study, he/she may request destruction of any samples taken and not tested, and the investigator must document this in the site study records.

8.2.3. Study Termination

The Sponsor reserves the right to close the study site or terminate the study at any time for any reason. Study sites will be closed upon study completion. A study site is considered closed when all required documents and study supplies have been collected and a study-site closure visit has been performed.

Reasons for the early closure of a study site by the Sponsor may include, but are not limited to:

- Failure of the investigator to comply with the protocol, the requirements of the IRB/IEC or local health authorities, the Sponsor's procedures, or GCP guidelines.
- Inadequate recruitment of subjects by the investigator.
- Discontinuation of further study treatment development.

9. STUDY ASSESSMENTS AND PROCEDURES

Planned time points for all assessments and procedures are provided in the Schedule of Assessments (Table 1).

9.1. Informed Consent

All subjects must provide written informed consent before any study specific procedures are performed in accordance with to ICH and applicable EC requirements.

9.2. Pharmacokinetic Measurements

Plasma concentrations of PU-AD will be determined from samples collected immediately following the infusion as specified in Table 1.

The date and actual time of sample collection will be recorded in the source documents and the CRF.

Samples will be analyzed by a central laboratory. Refer to the Laboratory Manual for details regarding sample processing, storage, and shipment.

9.3. Medical Imaging

Please reference the Imaging Manual for detail regarding medical imaging.

9.3.1. PET-CT scan

After ^{124}I-PU-AD tracer injection, PET scans will be performed at the following time-points: immediately, post-injection; 2.5-4.5 hours (+/- 30 mins), post-injection; and 1-2 days, post-injection. Optionally, in willing patients, an additional PET scan will be obtained 3-7 days post-injection.

At each time-point, a 30-60 minute axial body image (spanning from skull vertex to proximal thigh regions) is acquired on a state-of-the-art PET-CT scanner. A low-dose CT will be obtained immediately-prior to PET imaging, at each time-point. A 30-60 minute scanning time-period is typical for clinical nuclear medicine diagnostic imaging studies.

During positron emission tomography, subjects will be monitored visually and communication will be maintained directly between the patient and the radiology investigators, except during the CT, when communication will be maintained via a speaker system. The radiology investigators will evaluate patients during early time-period, post-injection.

9.3.2. Brain MRI

Subjects will have a standard non-contrast brain MRI 24-48 hours post-injection (i.e., during the 1-2 day post-injection PET time point). The brain MRI will allow for precise delineation of brain structures, which will be used in fusion PET/MRI analyses.

If a study subject has a suitable available brain MRI study that was performed within 2 months prior to enrollment, then the brain MRI need not be repeated.

9.4. Safety Assessments

Planned time points for all safety assessments are provided in the Schedule of Assessments (Table 1).

9.4.1. Pregnancy Test

A serum pregnancy test will be performed for all women of childbearing potential (Appendix 2). Pregnancy tests will be performed locally during screening. If a pregnancy is confirmed after study drug administration, the pregnancy will be reported and followed per Appendix 2. If a pregnancy is confirmed prior to study drug administration, the subject will be discontinued and will not receive the injection.

9.4.2. Physical Examinations

An abbreviated physical examination will be performed at the screening visit. During the abbreviated exam, the subject will be asked about changes in physical status and a symptom-directed physical examination will be accordingly conducted.

9.4.3. Protocol-Required Safety Laboratory Assessments

Blood samples will be collected and analyzed by a local laboratory according to the schedule in Table 1. Details regarding collection of samples, shipment of samples, reporting of results, laboratory normal ranges and alerting of abnormal values will be detailed by the local laboratory.

Investigators will be asked to comment on any abnormalities on the respective lab result page, including a notation of the clinical significance of each abnormal finding in the subject’s source documents. A laboratory abnormality may meet the criteria to qualify as an AE as described in this protocol and the CRF Completion Guidelines. In these instances, the AE corresponding to the laboratory abnormality will be recorded on the AE CRF.

Additional unscheduled laboratory evaluations may be done if clinically indicated.

9.4.4. Telephone Contact

On Day 30, a telephone call will be made in order to assess any AEs and changes in medications. A minimum of 3 attempts should be made and documented before documenting a subject as non-responsive.

9.5. Adverse Events and Serious Adverse Events

9.5.1. Definitions

9.5.1.1. Adverse Event

Adverse event means any untoward medical occurrence associated with the use of a drug in humans, whether or not considered drug-related.

An AE can be any unfavorable and unintended sign (i.e., an abnormal laboratory finding), symptom, or disease temporally associated with the use of a drug, and does not imply any judgment about causality.

Worsening of a pre-existing medical condition, should be considered an AE if there is either an increase in severity, frequency, or duration of the condition or an association with significantly worse outcomes.

Interventions for pretreatment conditions (i.e., elective cosmetic surgery) or medical procedures that were planned before study enrollment are not considered AEs.

The Investigator is responsible for reviewing laboratory test results and determining whether an abnormal value in an individual subject represents a significant change from baseline. In general, abnormal laboratory findings without clinical significance (based on the Investigator's judgment) should not be recorded as AEs; however, laboratory value changes requiring therapy or adjustment in prior therapy are considered AEs.

Adverse event means any untoward medical occurrence associated with the use of a drug in

A treatment emergent AE (TEAE) is defined as an AE that starts after the first dose of study drug or a pre-existing condition that worsens during treatment.

Only those AEs that were treatment emergent will be included in summary tables. All AEs, treatment emergent or otherwise, will be presented in subject data listings.

9.5.1.2. Serious Adverse Event

An SAE is any untoward medical occurrence that at any dose:

- is fatal
- is life-threatening (i.e., places the subject at immediate risk of death)
- results in persistent or significant disability/incapacity
- is a congenital anomaly/birth defect
- requires in-subject hospitalization or prolongation of existing hospitalization, unless hospitalization is for:
    - routine treatment or monitoring of the studied indication, not associated with any deterioration in condition (i.e., hospitalization for relapse treatment)
    - elective or pre-planned treatment for a pre-existing condition that is unrelated to the indication under study and has not worsened since the start of study drug
    - treatment on an emergency outpatient basis for an event not fulfilling any of the definitions of a SAE given above and not resulting in hospital admission
    - social reasons and respite care in the absence of any deterioration in the subject's general condition
    - is medically significant, i.e. defined as an event that jeopardizes the subject or may require medical or surgical intervention to prevent one of the outcomes listed above
- is an important medical event that may jeopardize the subject or may require medical intervention to prevent one of the outcomes listed above.

All SAEs should be submitted promptly to the Institutional Review Board/Independent Ethics Committee (IRB/IEC). The investigator must make an effort to obtain all hospital medical

records including discharge summary confirming final diagnosis. The death of a subject must be immediately (within 24 hours of Investigator becoming aware of the event) reported to the IRB. All serious and non-serious AEs should be thoroughly documented and followed out by the Investigator until the event resolves or deemed chronic by the Investigator. The event may be followed longer, if deemed necessary.

9.5.2. Adverse Events Assessment

The investigator and any designees are responsible for detecting, documenting, and recording events that meet the definition of an AE or SAE and remain responsible for following up AEs that are serious, considered related to the study dose or study procedures, or that caused the subject to discontinue the study.

The occurrence of AEs should be sought by non-directive questioning of the subject at each visit during the study. Adverse events may also be detected when they are volunteered by the subject during or between visits or through physical examination, laboratory test, or other assessments.

AE reporting should include the following information:

- the severity grade
- its relationship to (suspected/not suspected)
    - the study drug
- its duration (start and end dates or if continuing at final exam)
- whether it constitutes a serious adverse event

Adverse Events are graded according to the NCI CTCAE Version 4.03.

9.5.2.1. Severity

Each AE in this study will be assessed for severity. Severity will be assessed according to the NCI CTCAE Version 4.03. The NCI CTCAE displays Grades 1 through 5 with unique clinical descriptions of severity for each AE that are based on this general guideline.

- Mild (Grade 1): Asymptomatic or mild symptoms: clinical or diagnostic observations only; intervention not indicated.
- Moderate (Grade 2): Minimal, local, or non-invasive intervention indicated; limited age-appropriate instrumental activities of daily living.
- Severe (Grade 3): Severe or medically significant but not immediately life threatening: hospitalization or prolongation of hospitalization indicated; disabling; limiting self-care activities of daily living; incapacitating with inability to work or perform normal daily activity.
- Life-threatening (Grade 4): consequences: urgent intervention indicated.
- Death (Grade 5) related to an AE.

9.5.2.2. Relationship to Study Drug

The relationship of each AE to study drug is to be assessed by the Investigator. The Investigator should decide whether, in his or her medical judgment, there is a reasonable

possibility that the event may have been caused by the study drug. Items to be considered when assessing the relationship of an AE to the study drug are:

- Temporal relationship of the onset of the event to the initiation of the study drug.
- Course of the event, as applicable.
- If event is known to be associated with the study drug or with other similar drugs.
- Risk factors in the study subject known to increase the occurrence of the event.
- Presence of non-study, study drug-related factors that are known to be associated with the occurrence of the event.

9.5.2.3. Causality

The causality of each AE to study drug is to be assessed by the Investigator to determine whether there is a reasonable possibility that the study drug caused the AE using the categories presented in Table 2.

Table 2: Classifications for AE Causality

Classification	Definition
Related	There is an association between the event and the administration of investigational study drug, a plausible mechanism for the event to be related to the investigational study drug and causes other than the investigational study drug have been ruled out, and/or the event re-appeared on re-exposure to the investigational study drug.
Possibly related	There is an association between the event and the administration of the investigational study drug and there is a plausible mechanism for the event to be related to investigational study drug, but there may also be alternative etiology, such as characteristics of the subject's clinical status or underlying disease.
Unlikely related	The event is unlikely to be related to the investigational study drug and likely to be related to factors other than investigational study drug.
Not related	The event is related to an etiology other than the investigational study drug (the alternative etiology will be documented in the study subject's medical record).

9.5.2.4. Adverse Event Outcome

An AE will be followed until the investigator has determined and provided the final outcome. The outcome will be classified according to the categories shown in Table 3.

Table 3: Classifications for Outcome of an AE

Classification	Definition
**Recovered/Resolved**	Resolution of an AE with no residual signs or symptoms
**Recovered/ Resolved With Sequelae**	Resolution of an AE with residual signs or symptoms
**Not Recovered/Not Resolved (Continuing)**	Either incomplete improvement or no improvement of an AE, such that it remains ongoing
**Fatal**	Outcome of an AE is death. “Fatal” will be used when death is at least possibly related to the AE.
**Unknown**	Outcome of an AE is not known (i.e., a subject lost to follow-up)

9.5.3. Recording and Reporting of AE and SAE Information

9.5.3.1. Recording of Adverse Events

All SAEs will be collected and recorded from the time written informed consent is obtained through Day 30. All AEs will be collected and recorded from injection of study drug through Day 30. This includes AEs the subject reports spontaneously, those observed by the Investigator, and those elicited by the Investigator in response to open-ended questions during scheduled study visits.

All SAEs will be recorded and reported to the Sponsor or designee within 24 hours. The investigator will submit any updated SAE data to the Sponsor within 24 hours of it being available.

9.5.3.2. Reporting of Serious Adverse Events

The Investigator must report all SAEs within 24 hours of the Investigator’s discovery.

A completed SAE report is to be sent to the Medical Monitor’s attention within 24 hours of discovering the event. The initial report should include at least the following information:

- Subject’s ID number;
- Description and date of the event;
- Criterion for serious; and
- Preliminary assignment of causality to study drug.

The Medical Monitor will contact the Investigator via telephone for follow-up information regarding the SAE, as appropriate.

The Investigator, or designated party, should notify the appropriate IRB/EC of SAEs occurring at the study center and other AE reports received from the Sponsor, in accordance with local procedures and statutes.

9.5.3.3. Follow-up of AEs and SAEs

Once an AE is detected, it should be followed until its resolution or until it is judged to be permanent, and assessment should be made at each visit (or more frequently, if necessary) of any changes in severity, the suspected relationship to the study drug, the interventions required to treat it, and the outcome.

Investigators are not obligated to actively seek AE or SAE in former study subjects; however, if the investigator learns of any SAE, including death, at any time after a subject has been discharged from the study, and he/she considers the event to be reasonably related to the study treatment or study participation, the investigator must promptly notify the Sponsor.

9.5.3.4. Regulatory Reporting Requirements for SAEs

Prompt notification by the investigator to the Sponsor of a SAE is essential so that legal obligations and ethical responsibilities towards the safety of subjects and the safety of a study treatment under clinical investigation are met.

The Sponsor has a legal responsibility to notify both the local regulatory authority and other regulatory agencies about the safety of a study treatment under clinical investigation. The Sponsor will comply with country-specific regulatory requirements relating to safety reporting to the regulatory authority, (IRB/IEC), and investigators. Investigator safety reports must be prepared for suspected unexpected serious adverse reactions (SUSAR) according to local regulatory requirements and Sponsor policy and forwarded to investigators as necessary.

An investigator who receives an investigator safety report describing a SAE or other specific safety information (i.e., summary or listing of SAEs) from the Sponsor will review and then file it along with the Investigator's Brochure and will notify the IRB/IEC, if appropriate according to local requirements.

9.5.4. Pregnancy

Details of all pregnancies in female subjects and female partners of a male subject will be collected through 30 days after the injection of ^{124}I-PU-AD.

If a pregnancy is reported, the investigator should inform the Sponsor within 24 hours of learning of the pregnancy. Every effort should be made to gather information regarding the pregnancy outcome until 8 weeks post-partum. It is the Investigator's responsibility to obtain and report all pregnancy information to the sponsor.
Abnormal pregnancy outcomes (i.e., spontaneous abortion, fetal death, stillbirth, congenital anomalies, and ectopic pregnancy) are considered SAEs.

10. STATISTICAL CONSIDERATIONS

10.1. Sample Size Determination

The microdose first in human trial design, advocated by the FDA, employs a small pilot study to obtain preliminary first in-human data for Investigators to use in deciding whether further investment in development of a novel investigational compound, such as a diagnostic imaging agent, seems warranted. This is a small pilot study with a sample size of 10 subjects. This study will provide an opportunity to obtain initial observational data regarding radiotracer PK, metabolism, biodistribution, and radiation dosimetry – a major determinant of the probable clinical potential of a new diagnostic radiotracer – and, equally importantly, to explore iterative adjustments to the PET and blood radioassay methodology – identifying any technical issues and learning, for the first time, in iterative fashion over the 10 subjects in the study, how to perform a PET scan with the novel tracer, based upon its pharmacokinetics, metabolism, and biodistribution that are unknown, in human subjects, at present.

No other statistical considerations were implemented in choosing a sample size for this study. The small sample size is not suitable or intended for hypothesis testing. Summary data will be presented for clinical interpretation.

10.2. Populations for Analyses

For purposes of analysis, the following populations are defined:

Population	Description
Safety Analysis Set	Enrolled subjects who have taken the dose of study drug.
Pharmacokinetic Analysis Set	Subjects in the Safety Analysis Set with no major deviations related to study drug intake (i.e., vomiting), for whom the primary PK data is considered sufficient and interpretable.

10.3. Statistical Analyses

To preserve the integrity of the statistical analysis and study conclusions, the SAP will be developed and finalized before database lock and will describe the subject populations to be included in the analyses, and procedures for accounting for missing, unused, and spurious data. Details regarding the statistical methods and definitions will be provided in the SAP. All statistical analyses will be performed after the database is locked. Statistical analyses will be performed using Version 9.3 or higher of SAS® (SAS Institute, Cary, NC 27513).

10.3.1. Primary Analyses

The primary goal of this study is to study the first in-human PK, metabolism, biodistribution, and radiation dosimetry of ^{124}I-PU-AD. The primary analyses will be performed using the Pharmacokinetic Analysis Set.

Complete details can be found in the SAP.

10.3.1.1. Pharmacokinetic and Metabolites Analyses

Blood/serum analyses identify and quantify the presence of metabolites versus intact compound. The blood-based datasets shall be used to quantify the characterization of tracer-metabolism. This data shall be used to derive the quantitative PK parameters of tracer-uptake, -clearance and tracer-excretion. PK parameters of PU-AD and its metabolite (including, but not limited to C_{max}, T_{max}, $AUC_{0\text{-}24\ hr}$, AUC_{inf}, CL, and $t_{1/2}$) will be analyzed descriptively.

10.3.1.2. Biodistribution Analyses

^{124}I-PU-AD is injected as a single intravenous bolus. The patient then undergoes PET and blood assays, at multiple time-points; the PET and blood data is used to quantify tracer-biodistribution, measuring the amount of radioactivity present in the blood and bodily regions-of-interest, at each time-point (expressed, e.g., in terms of percentage injected-dose per unit tissue volume or mass). The biodistribution parameters of ^{124}I-PU-AD will be summarized using medians as well as graphically for each bodily region.

10.3.1.3. Radiation Dosimetry Analyses

The PK parameters are used to derive the radiation dosimetry parameters of absorbed-dose and effective-dose. The radiation dosimetry parameters will be summarized descriptively.

10.3.2. Secondary Analyses

10.3.2.1. Safety

Evaluation of safety will be performed on the Safety Analysis Set. Safety data to be evaluated include AEs and SAEs. Note that AEs will be observed for all patients through Day 2, and thereafter will be collected from telephone interviews through Day 30.

Toxicities will be assessed according to the National Cancer Institute CTC Scale (Version 4.0). In particular, subjects will be evaluated per standard of care for thyroid abnormalities approximately 6-12 months after study agent administration, by a serum assay of thyroid stimulating hormone (TSH). A baseline serum TSH will be obtained within 4 weeks prior to the ^{124}I-PU-AD injection. Thyroid ^{124}I uptakes doses will be measured.

AEs will be coded using MedDRA (Version 15.0 or higher). An overall summary of AEs will be provided, as well as summaries of treatment-emergent AEs (TEAE) by MedDRA system organ class (SOC) and preferred term (PT). TEAEs by SOC and PT will be provided overall, for treatment-related TEAEs, by maximal severity, and by study day of onset.

10.3.3. Other Analyses

Subject Disposition

Subjects in each analysis set, as well as subjects who complete the study, and subjects who prematurely discontinue from the study will be summarized using descriptive statistics. In addition, for subjects who prematurely discontinue from the study, the reasons for discontinuation will be summarized.

Demographic and Baseline Characteristics

Descriptive summaries of demographic and baseline characteristics will be presented for the Safety Analysis Set.

Prior and Concomitant Medication

Prior and concomitant medications will be coded using the World Health Organization (WHO) drug dictionary (WHODRUG/2006QA or newer version). Prior and concomitant medications will be listed and summarized by preferred drug name.

11. REGULATORY AND ETHICAL CONSIDERATIONS

11.1 Good Clinical Practice

This study will be conducted according to the protocol and in compliance with ICH GCP, the ethical principles stated in the Declaration of Helsinki, and other applicable regulatory requirements.

The Investigator confirms this by signing the protocol.

11.2 Informed Consent Process

Written informed consent, in compliance with 21 Code of Federal Regulations (CFR) § 50 will be obtained from each subject prior to undergoing any protocol-specific tests or procedures.

The investigator or his/her representative will explain the nature of the study to the subject or his/her LAR and answer all questions regarding the study.

Subjects must be informed that their participation is voluntary. Subjects or their LAR will be required to sign a statement of informed consent that meets the requirements of 21 CFR 50, local regulations, ICH guidelines, Health Insurance Portability and Accountability Act (HIPAA) requirements, where applicable, and the IRB/IEC or study center.

The medical record must include a statement that written informed consent was obtained before the subject was enrolled in the study and the date the written consent was obtained. The authorized person obtaining the informed consent must also sign the ICF.

Subjects must be re-consented to the most current version of the ICF(s) during their participation in the study unless otherwise specified by the IRB/IEC. A copy of the ICF(s) must be provided to the subject or the subject's LAR.

11.3 Institutional Review Board

Federal regulations and ICH require that approval be obtained from an IRB/EC prior to participation of subjects in research studies. Prior to the study onset, the protocol, any protocol amendments, ICFs, advertisements to be used for subject recruitment, and any

other written information regarding this study to be provided to a subject, must be approved by the IRB/EC.

All IRB/EC approvals must be dated and signed by the IRB/EC Chairperson or designee and must identify the IRB/EC by name and address, the clinical protocol by title and/or protocol number, and the date approval or favorable opinion was granted for the clinical research.

No drug will be released to the site to dose a subject until written IRB/EC authorization has been received by Samus or designee.

The Investigator is responsible for obtaining continuing review of the clinical research at least annually or more often if specified by the IRB/EC. The Investigator must supply Samus or designee with written documentation of the approval of the continued clinical research.

The Investigator, Sponsor, or designee as applicable, will make all attempts to ensure that the IRB/EC is constituted and operates in accordance with Federal and ICH GCP and any local regulations.

11.4 Publication Policy

All manuscripts, abstracts, or other modes of presentation arising from the results of the study must be reviewed and approved in writing by the Sponsor, in advance of submission. The review is aimed at protecting the Sponsor's proprietary information existing either at the date of the commencement of the study or generated during the study.

12. STUDY MANAGEMENT

12.1. Data Quality Assurance

This study will be organized, performed, and reported in compliance with the protocol, Standard Operating Procedures, working practice documents, and applicable regulations and guidelines. Site audits may be made periodically by the Sponsor's or the CRO's qualified compliance auditing team, which is an independent function from the study team responsible for conduct of the study.

12.2. Case Report Forms and Source Documentation

The Investigator and designees agree to maintain accurate CRFs and source documentation as part of case histories. Source documents are the originals of any documents used by the Investigator or sub investigator or hospital/institution that allow verification of the existence of the subject and substantiate the integrity of the data collected during the study.

Samus or designee will provide CRFs to the study center. Case Report Forms will be completed for each subject. It is the Investigator's responsibility to ensure the accuracy, completeness, and timeliness of the data reported in the subject's CRF. Source documentation supporting the CRF data should indicate the subject's participation in the study and should document the dates and details of informed consent, study procedures, AEs, and subject status.

The Investigator, or designated representative, should complete the CRF as soon as possible after information is collected/data are available, preferably on the same day that a subject is seen for an examination, treatment, or any other study procedure. Any outstanding entries must be completed immediately after the final examination. An explanation should be given for all missing data.

The Investigator must sign and date the Investigator's Statement at the end of the CRF to endorse the recorded data.

12.3. Monitoring

A clinical research associate (CRA), or other representative of the Sponsor, will conduct a study center visit to verify the qualifications of each Investigator, inspect study center facilities, and inform the Investigator of responsibilities and procedures for ensuring adequate and correct study documentation.

During the course of the study, the CRA will make study center visits to review protocol compliance, compare CRFs and individual subjects' medical records, assess drug accountability, and ensure that the study is being conducted according to pertinent regulatory requirements in respect to GCP. Case Report Forms will be verified with source documentation. The review of medical records will be performed in a manner to ensure that subject confidentiality is maintained.

The Investigator will allow the CRA to inspect the clinical, laboratory, and pharmacy facilities to assure compliance with GCP and local regulatory requirements. The CRFs and subject's corresponding original medical records (source documents) are to be fully available for review by the Sponsor's representatives at regular intervals. These reviews verify adherence to study protocol and data accuracy in accordance with local regulations.

Monitoring visits to each site will be conducted by the assigned CRA as described in the monitoring plan.

12.4. Inspections

Regulatory authorities and/or quality assurance personnel from Samus, or its designated representative, may wish to carry out such source data checks and/or in-center audit inspections. The Investigator assures Samus of the necessary support at all times. In the event of an audit, the Investigator agrees to allow the Sponsor's representatives and any regulatory agencies access to all study records.

12.5. Financial Disclosure Reporting Obligations

Investigators and sub-investigators are required to provide financial disclosure information to the Sponsor to permit the Sponsor to fulfill its regulatory obligation. A financial disclosure form will be completed by Investigators and sub-investigators prior to starting the study and at the end of the study. Investigators and sub-investigators must commit to promptly updating the information if any relevant changes occur during the study and for a period of 1 year after the completion of the study.

12.6. Archiving Study Records

Essential documents should be retained for a minimum of 2 years after the last approval of a marketing application in an ICH region and until there are no pending or contemplated marketing applications in an ICH region or at least 2 years have elapsed since the formal discontinuation of clinical development of the study drug. However, these documents should be retained for a longer period if required by the applicable local requirements.

ICH requires that subject identification codes be retained for at least 15 years after the completion or discontinuation of the study.

13. REFERENCES

1. Workman, P., F. Burrows, L. Neckers, *et al.* (2007). Drugging the cancer chaperone HSP90: combinatorial therapeutic exploitation of oncogene addiction and tumor stress. Ann N Y Acad Sci. 1113:202-216.

2. Whitesell, L., and Lindquist, S.L. (2005). HSP90 and the chaperoning of cancer. Nat. Rev. Cancer 5, 761-772.

3. Chiosis, G. (2006). Discovery and development of purine-scaffold HSP90 inhibitors. Curr Top Med Chem. 6:1183-1191.

4. Kamal A, Thao L, Sensintaffar J, Zhang L, Boehm MF, Fritz LC, et al. A high-affinity conformation of HSP90 confers tumour selectivity on HSP90 inhibitors. Nature 2003;425:407-410.

5. Kucine, N., S. Marubayashi, N. Bhagwat, E. Papalexi, P. Koppikar, M. Sanchez Martin, L. Dong, M. S. Tallman, E. Paietta, K. Wang, J. He, D. Lipson, P. Stephens, V. Miller, J. M. Rowe, J. Teruya-Feldstein, C. G. Mullighan, A. A. Ferrando, A. Krivtsov, S. Armstrong, L. Leung, S. O. Ochiana, G. Chiosis, R. L. Levine, and M. Kleppe. "Tumor-specific HSP90 Inhibition as a Therapeutic Approach in JAK-mutant Acute Lymphoblastic Leukemias." Blood 126.22 (2015): 2479-483.

6. Taldone, Tony, Stefan O. Ochiana, Pallav D. Patel, and Gabriela Chiosis. "Selective Targeting of the Stress Chaperome as a Therapeutic Strategy." Trends in Pharmacological Sciences 35.11 (2014): 592-603.

7. Culjkovic-Kraljacic, B., T. M. Fernando, R. Marullo, N. Calvo-Vidal, A. Verma, S. Yang, F. Tabbo, M. Gaudiano, H. Zahreddine, R. L. Goldstein, J. Patel, T. Taldone, G. Chiosis, M. Ladetto, P. Ghione, R. Machiorlatti, O. Elemento, G. Inghirami, A. Melnick, K. L. B. Borden, and L. Cerchietti. "Combinatorial Targeting of Nuclear Export and Translation of RNA Inhibits Aggressive B-cell Lymphomas." Blood 127.7 (2016): 858-68.

8. Shrestha, Liza, HardikÂ J. Patel, and Gabriela Chiosis. "Chemical Tools to Investigate Mechanisms Associated with HSP90 and HSP70 in Disease." Cell Chemical Biology 23.1 (2016): 158-72.

9. Corben, Adriana D., Mohammad M. Uddin, Brooke Crawford, Mohammad Farooq, Shanu Modi, John Gerecitano, Gabriela Chiosis, and Mary L. Alpaugh. "Ex Vivo Treatment Response of Primary Tumors And/or Associated Metastases for Preclinical and Clinical Development of Therapeutics." Journal of Visualized Experiments 92 (2014): n. pag.

10. Nayar U, Lu P, Goldstein RL, Vider J, Ballon G, Rodina A, Taldone T, Erdjument-Bromage H, Chomet M, Blasberg R, Melnick A, Cerchietti L, Chiosis G, Wang YL, Cesarman E. Targeting the HSP90- associated viral oncoproteome in gammaherpesvirus- associated malignancies. Blood. 2013 Oct 17. 122(16): 2837-47.

11. Kralovics R, *et al.* A gain-of-function mutation of JAK2 in myeloproliferative disorders. N Engl J Med. 2005;352(17):1779–1790.

12. Lista S et al. Evolving Evidence for the Value of Neuroimaging Methods and Biological Markers in Subjects Categorized with Subjective Cognitive Decline. J Alzheimers Dis. 2015 48 Suppl 1:S171-91.

13. Carman, Kishinevsky, Koren, Lou, Chiosis. Chaperone-Dependent Neurodegeneration: A Molecular Perspective on Therapeutic Intervention. Journal of Alzheimer's Disease & Parkinsonism [01 Apr 2013, 2013 (Suppl 10)]

14. McKhann, et al. The Diagnosis of Dementia Due to Alzheimer's Disease: Recommendations from the National Institute on Aging – Alzheimer's Association Workgroups on Diagnostic Guidelines for Alzheimer's Disease. Alzheimers Dement 2011;May;7(3):263-9. doi: 10.1016/j.jalz.2011.03.005.

14. APPENDICES

Appendix 1: Abbreviations and Trademarks

AE	Adverse event
ALT	Alanine transaminase
ANC	Absolute Neutrophil Count
AST	Aspartate transaminase
AUC	Area under the curve
CFR	Code of Federal Regulations
Cmax	Maximum plasma concentration
Cmin	Minimum plasma concentration
CRA	Clinical Research Associate
CRF	Case Report Form
CT	Computerized Tomography
CTCAE	Common Terminology Criteria for Adverse Events
CSR	Clinical Study Report
FDA	Food and Drug Administration
GCP	Good Clinical Practice
HCG	Human chorionic gonadotropin
HIPAA	Health Insurance Portability and Accountability Act
HSP	Heat Shock Protein
IB	Investigator’s Brochure
ICF	Informed Consent Form
ICH	International Conference on Harmonisation
IEC	Independent Ethics Committee
IND	Investigational New Drug
IRB	Institutional Review Board
IV	Intravenous
MRI	Magnetic Resonance Imaging
MSKCC	Memorial Sloan Kettering Cancer Center
NCI	National Cancer Institute
PET	Positron Emission Tomography
PK	Pharmacokinetic
PT	Preferred Term

SAE	Serious Adverse Event
SAP	Statistical Analysis Plan
SOC	System Organ Class
TEAE	Treatment-emergent Adverse Events
t_{max}	Time to maximum plasma concentration
WHO	World Health Organization

Appendix 2: Contraceptive Guidance & Collection of Pregnancy Information

Definitions
Woman of Childbearing Potential: *A woman is considered fertile following menarche and until becoming post-menopausal unless permanently sterile (see below).*

Women in the following categories are not considered of childbearing potential

1. Premenarchal
2. Premenopausal female with 1 of the following:
   - Documented hysterectomy, bilateral salpingectomy, bilateral oophorectomy
3. Postmenopausal female:
   - A postmenopausal state is defined as no menses for 12 months without an alternative medical cause. A high follicle stimulating hormone (FSH) level in the postmenopausal range may be used to confirm a postmenopausal state in women not using hormonal contraception or hormonal replacement therapy (HRT). However, in the absence of 12 months of amenorrhea, a single FSH measurement is insufficient.
   - Females on HRT and whose menopausal status is in doubt will be required to use one of the non-hormonal highly effective contraception methods if they wish to continue their HRT during the study. Otherwise, they must discontinue HRT to allow confirmation of postmenopausal status before study enrollment.

CONTRACEPTION GUIDANCE
Female subjects of childbearing potential are eligible to participate if they agree to use a highly effective method of contraception consistently and correctly as described below:

Highly Effective Contraceptive Methods That Are User Dependent

1. Combined (estrogen and progestogen containing) hormonal contraception associated with inhibition of ovulation (Oral, Intravaginal, Transdermal)
2. Progestogen only hormonal contraception associated with inhibition of ovulation (Oral, Injectable)

Highly Effective Methods That Are User Independent

1. Implantable progestogen only hormonal contraception associated with inhibition of ovulation (Intrauterine device (IUD), Intrauterine hormone-releasing system (IUS), Bilateral tubal occlusion)
2. **Vasectomized partner** *A vasectomized partner is a highly effective contraception method provided that the partner is the sole male sexual partner of the woman of childbearing potential and the absence of sperm has been confirmed. If not, an additional highly effective method of contraception should be used.*
3. **Sexual abstinence** *Sexual abstinence is considered a highly effective method only if defined as refraining from heterosexual intercourse during the entire period of risk associated with the study treatment.*

Male subjects with female partners of childbearing potential are eligible to participate if they agree to ONE of the following:

- *Abstinence as their usual and preferred lifestyle (abstinent on a long term and persistent basis) and agree to remain abstinent*
- *Agree to use a male condom plus partner use of a contraceptive method.*

Appendix 3: Investigator Statement

I have read this protocol and agree to conduct this study in accordance with all stipulations of the protocol and in accordance with International Conference on Harmonisation of Technical Requirements for Registration of Pharmaceuticals for Human Use (ICH) and all applicable local Good Clinical Practice (GCP) guidelines, including the Declaration of Helsinki.

______________________________   ______________________________

Principal Investigator's Signature   **Date of Signature**

____

Principal Investigator's Printed Name
