# Supplementary material for: Chemical tools for epichaperome-mediated interactome dysfunctions of the central nervous system
Source: Nat Commun. 2021 Aug 3;12:4669. doi: 10.1038/s41467-021-24821-2 (PMC8333062; doi:10.1038/s41467-021-24821-2)
Supplement: Supplementary file 3 — Reporting Summary [file 41467_2021_24821_MOESM3_ESM.pdf]

nature research

Corresponding author(s): Gabriela Chiosis and Fumiko Shimizu

Last updated by author(s): May 17, 2021

Reporting Summary

Nature Research wishes to improve the reproducibility of the work that we publish. This form provides structure for consistency and transparency in reporting. For further information on Nature Research policies, see Authors & Referees and the Editorial Policy Checklist .

Statistics

For all statistical analyses, confirm that the following items are present in the figure legend, table legend, main text, or Methods section.

n/a	Confirmed	
[ ]	[x]	The exact sample size (*n*) for each experimental group/condition, given as a discrete number and unit of measurement
[ ]	[x]	A statement on whether measurements were taken from distinct samples or whether the same sample was measured repeatedly
[ ]	[x]	The statistical test(s) used AND whether they are one- or two-sided<br>*Only common tests should be described solely by name; describe more complex techniques in the Methods section.*
[x]	[ ]	A description of all covariates tested
[ ]	[x]	A description of any assumptions or corrections, such as tests of normality and adjustment for multiple comparisons
[ ]	[x]	A full description of the statistical parameters including central tendency (e.g. means) or other basic estimates (e.g. regression coefficient) AND variation (e.g. standard deviation) or associated estimates of uncertainty (e.g. confidence intervals)
[ ]	[x]	For null hypothesis testing, the test statistic (e.g. *F*, *t*, *r*) with confidence intervals, effect sizes, degrees of freedom and *P* value noted<br>*Give P values as exact values whenever suitable.*
[x]	[ ]	For Bayesian analysis, information on the choice of priors and Markov chain Monte Carlo settings
[x]	[ ]	For hierarchical and complex designs, identification of the appropriate level for tests and full reporting of outcomes
[ ]	[x]	Estimates of effect sizes (e.g. Cohen's *d*, Pearson's *r*), indicating how they were calculated

Our web collection on statistics for biologists contains articles on many of the points above.

Software and code

Policy information about availability of computer code

Data collection

As described in the Methods, the identity and purity of each product was characterized by MS, HPLC, TLC, and NMR. 1H/13C NMR spectra were recorded on either a Bruker 400, 500 or 600 MHz instrument. High resolution mass spectra were recorded on a Waters LCT Premier system. Low resolution mass spectra were obtained on Waters Acquity Ultra Performance LC with electrospray ionization and SQ detector. HPLC analysis was done on Waters Autopurification system with PDA, MicroMass ZQ and ELSD detector and a reversed phase column (Waters X-Bridge C18, 4.6 x 150 mm, 5 µm) eluted with water/acetonitrile gradients, containing 0.1% TFA. FP measurements were performed on an Analyst GT instrument (Molecular Devices, Sunnyvale, CA). General SEP II was performed by Caliper Life Sciences (now PerkinElmer). Radioactivity metabolite analysis was performed using Shimadzu (Columbia, Maryland USA) HPLC system equipped with binary pumps LC-20AB, UV detector and sodium iodide radioactivity detector connected to flow-ram (Lab Logic, Tampa, FL) and 250 x 4.6 mm Phenomenex Luna (Torrance, CA) C-18 HPLC column (10 µM, 100 A). For metabolite characterization, samples were analyzed by LC-MS using positive-ion electrospray ionization and reverse-phase chromatography in full-scan mode with MS/MS product-ion spectra generated for detected metabolite masses. This was performed using the MS2 scan mode of the G6410B mass spectrometer to produce MS/MS product-ion spectra for masses detected at sufficient intensities. Chromatography was performed using a ZORBAX Eclipse XDB-C18 4.6 x 50 mm, 5 µm, column, with gradient elution at 1mL /min-1. Absorbance was measured by UV spectroscopy on the GT Analyst (Molecular Devices) plate reader at 260 nm. Radiation (10 Gy) was delivered via X-RAD 225C irradiator (Precision X-Ray Inc., North Branford, CT). CD105+ tumor endothelial cells were isolated by a MoFlo cell sorter (Beckman Coulter, Pasadena, CA). I-131 was measured in a scintillation g-counter (Perkin Elmer 1480 Wizard 3 Auto Gamma counter, Waltham, MA) using a 260-340 keV energy window. For mouse PET imaging, small-animal PET scanner (Model # Focus 120 microPET; Siemens Medical Solutions, USA, formerly Concorde Microsystems, Knoxville, TN)) was used. LC-MS/MS (6410, Agilent Technologies) was used to measure concentrations of PU-HZ151 in mouse plasma and tissues. PET-CT in human patients was performed using an integrated PET-CT scanner (Discovery DSTE, GE Healthcare) and MRI was obtained using a 1.5 T magnet (Signa HDxt, GE Healthcare, Milwaukee, WI). For quantification of radioprobe clearance and metabolism, plasma samples were radioassayed by well counter and radioHPLC using standard techniques. Activity in whole blood and plasma specimens was radioassayed by well counter (1480 WIZARD® 3" Automatic Gamma Counter, PerkinElmer, Shelton, Connecticut USA).

Data analysis

Prism v7and9 was used for statistical testings (t-tests and ANOVA). ImageJ (versions 1.4 and 1.52) was used for western blot quantification. SoftMaxPro6 was used for fluorescence polarization data analyses. Calculated physicochemical properties were obtained using standard commercial and freeware packages: Schrödinger software suite release 2018-1 (QikProp and Epik), ChemAxon Marvin suite (MarvinSketch 20.19) and VCCLAB (ALOGPS 2.1). The 2D structures of all the title compounds were generated by using ChemBioDraw Ultra 12.0. LigPrep module implemented in Schrödinger was used to generate energy minimized 3D structures. Acquired flow cytometry data was analysed using FlowJo v10.7 (FlowJo LLC.). Quantification and %ID g-1 values were calculated by manually drawing regions of interests in three different frames and determining the average values using ASI Pro VM™ MicroPET Analysis software (Siemens Medical Solutions, Knoxville, TX). For precise localization of tracer uptake to specific neuroanatomical structures, PET brain images were fused to MRI brain images using the Integrated Registration application of the AW Suite software package (GE Healthcare, Milwaukee, Wisconsin, USA). The software used for the fits and to derive the PK parameters was EXCEL 365 (Microsoft Corp, Redmon, WA). Relative count intensity of the autoradiography sections in each image was quantified using ImageJ 1.47u processing software.

For manuscripts utilizing custom algorithms or software that are central to the research but not yet described in published literature, software must be made available to editors/reviewers. We strongly encourage code deposition in a community repository (e.g. GitHub). See the Nature Research guidelines for submitting code & software for further information.

Data

Policy information about availability of data

All manuscripts must include a data availability statement. This statement should provide the following information, where applicable:

- Accession codes, unique identifiers, or web links for publicly available datasets
- A list of figures that have associated raw data
- A description of any restrictions on data availability

The source data underlying all main and supplementary figures are provided as a Source Data file. All data generated or analyzed during this study are included in this published article (and its supplementary information files).

Field-specific reporting

Please select the one below that is the best fit for your research. If you are not sure, read the appropriate sections before making your selection.

[x] Life sciences   [ ] Behavioural & social sciences   [ ] Ecological, evolutionary & environmental sciences

For a reference copy of the document with all sections, see nature.com/documents/nr-reporting-summary-flat.pdf

Life sciences study design

All studies must disclose on these points even when the disclosure is negative.

Sample size

No statistical methods were used to predetermine sample size for in vitro but these are similar to those generally employed in the field. For mouse studies, sample sizes were determined by magnitude and consistency of measurable differences in a pilot study we conducted. Sample sizes for in vitro and in vivo experiments are fully disclosed in the manuscript. For the clinical study, this was an exploratory pilot study. Sample was not estimated according to statistical power calculation. In vitro and in vivo experiments were performed in at least 3 biological replicates to sufficiently detect statistical significance, and the number of technical replicates for in vitro studies were determined according to manufacturers' instructions for data acquisitions.

Data exclusions

No data were excluded from the analyses. For the clinical study (NCT03371420), the first enrolled patient consented but withdrew prior to participating in the study due to disease worsening. For the third enrolled patient we had a failure in 124I-PU-HZ151 production and the patient could not complete the study. Exclusion Criteria for participation in NCT03371420: Subject has unacceptable pre-study organ function during screening defined as:

Bilirubin > 1.5 x institutional upper limit of normal (ULN)

AST/ALT >2.5 x ULN

Albumin < 2 g/dl

GGT > 2.5 x ULN (IF Alkaline phosphatase > 2.5 x ULN)

Creatinine >1.5 x ULN or creatinine clearance < 60 mL/min.

Subject has history of acute major illness (i.e., unstable cardiovascular condition.)

Subject has concurrent participation in any interventional studies within 30 days of first dose of study drug.

Replication

All in vitro and in vivo experiments were performed, in at least 3 biological replicates, with biological and technical replicates which are fully disclosed in the manuscript. Several alternative methods were used to validate observations. Experiments were also replicated through multiple cohort analyses. Results shown are representative of several independently performed experiments (see figure legends, at least 3). There were no findings that could not be replicated or reproduced. The data in the clinical analyses were derived from a clinical trial; therefore, experimental replication was not feasible.

Randomization

Mice were randomized prior to treatment with vehicle control or compound. For in vitro experiments, samples were allocated into experimental groups by randomization. For the clinical PET imaging study randomization does not apply. This is a single group assignment diagnostic study (ClinicalTrials.gov Identifier: NCT03371420).

Blinding

Investigators were not blinded to group allocation during data collection and/or analysis for the preclinical studies. All values were determined by methods that are independent of operator bias. Mice treatments were not blinded since most of the experiments required daily treatments and treatment groups and mice cage numbers had to be known for investigators. For the clinical study, outcome assessment for

the primary endpoint (blood PK parameters) was performed by strictly blinded scientists. Blinding was not relevant to the remaining experiments described in the study due to primary investigators performing experiments from start to finish due to technicality required.

Reporting for specific materials, systems and methods

We require information from authors about some types of materials, experimental systems and methods used in many studies. Here, indicate whether each material, system or method listed is relevant to your study. If you are not sure if a list item applies to your research, read the appropriate section before selecting a response.

Materials & experimental systems

n/a	Involved in the study
☐	☒ Antibodies
☐	☒ Eukaryotic cell lines
☒	☐ Palaeontology
☐	☒ Animals and other organisms
☐	☒ Human research participants
☐	☒ Clinical data

Methods

n/a	Involved in the study
☒	☐ ChIP-seq
☐	☒ Flow cytometry
☐	☒ MRI-based neuroimaging

Antibodies

Antibodies used

All antibodies and relevant information is provided in the Methods. Antibodies are listed below with target, clone, supplier (Biolegend unless otherwise stated), dilution and catalog number: HSP90β (H90-10) (SMC-107; RRID:AB_854214; 1:3000) and HSP110 (SPC-195; RRID:AB_2119373; 1:2000) were purchased from StressMarq; HSP90α (ab2928; RRID:AB_303423; 1:6000) and Olig2 (ab109186; RRID:AB_10861310; 1:1000) from Abcam; β-actin (A1978; RRID:AB_476692; 1:3000) from Sigma-Aldrich; p53 (2524; RRID:AB_331743; 1:2000), PTEN (9188; RRID:AB_2253290; 1:1000), Bcl-2 (15071; RRID:AB_2744528; 1:1000), EGFR (2085; RRID:AB_1903953; 1:1000), PDGFRα (3174; RRID:AB_2162345; 1:1000), p-AKT (Ser473) (4060; RRID:AB_2315049; 1:1000), p-AKT (Thr308) (13038; RRID:AB_2629447; 1:1000), AKT (4691; RRID:AB_915783; 1:2000), p-ERK1/2 (Thr202/Tyr204) (4370; RRID:AB_2315112; 1:2000), ERK 1/2 (9102; RRID:AB_330744; 1:4000), Bim (2933; RRID:AB_1030947; 1:1000), Bad (9268; RRID:AB_10695002; 1:1000), Bid (2002; RRID:AB_10692485; 1:1000), Bcl-xL (2764; RRID:AB_2228008; 1:1000), Bcl-w (2724; RRID:AB_10691557; 1:1000), Mcl-1 (5453; RRID:AB_10694494; 1:1000), GAPDH (5174; RRID:AB_10622025; 1:5000), RB (9309; RRID:AB_823629; 1:1000), p-RB (Ser807/811) (8516; RRID:AB_11178658; 1:1000), NF1 (14623; RRID:AB_2798543; 1:1000), HOP (5670; RRID:AB_10828378; 1:2000), EGFR (4267; RRID:AB_2246311; 1:1000), cleaved Caspase-3 (9661; RRID:AB_2070042; 1:100), Pgp, also called multi-drug resistance protein 1 (MDR1) (12683; RRID:AB_2715689; 1:1000) and CDC37 (4793; RRID:AB_10695539; 1:2000) from Cell Signaling Technology; HSP70 (ADI-SPA-810; RRID:AB_10616513; 1:2000), HOP (ADI-SRA-1500; RRID:AB_10618972; 1:2000) and HSC70 (ADI-SPA-815; RRID:AB_10617277; 1:4000) from Enzo and cleaved PARP, p85 fragment (G7341; RRID:AB_430876; 1:1000) from Promega; Ki67 (GA62661-2; RRID:AB_2687921; 1:100) and CD105 (1:100, Dako) (M3527; RRID:AB_2099044; 1:100) from Dako; CD31 (550274; RRID:AB_393571; 1:100) and FITC-conjugated CD105 antibody (561443; RRID:AB_10714629; 1:20) from BD Biosciences.

Validation

All antibodies are commercially available and have been validated by the manufacturer. Supporting publications are found on the manufacturer's site. Relevant positive and negative controls were used to further validate several antibodies as indicated in the relevant figures. Antibodies have been validated either from prior reports and studies or validated by the manufacturer as stated on the website from the catalog numbers listed above or published references on the manufacturers' websites. Manufacturer states the antibody has been validated for intended use. Manufacturer citation are listed in manufacturer website for each specific antibody.

Eukaryotic cell lines

Policy information about cell lines

Cell line source(s)

The MDA-MB-468 (HTB-132; RRID:CVCL_0419), Kasumi-1 (CRL-2724; RRID:CVCL_0589), HepG2 (HB-8056; RRID:CVCL_0027), and ASPC1 (CRL-1682; RRID:CVCL_0512) and HEK-193 (CRL-1573; RRID:CVCL_0045) human cancer cell lines were obtained from the American Type Culture Collection.

Authentication

Cell were authenticated using short tandem repeat profiling

Mycoplasma contamination

Cells were routinely tested for mycoplasma and were found to be negative

Commonly misidentified lines
(See ICLAC register)

none was used

Animals and other organisms

Policy information about studies involving animals; ARRIVE guidelines recommended for reporting animal research

Laboratory animals

All animal studies were conducted in compliance with MSKCC's guidelines and under Institutional Animal Care and Use Committee approved protocols #05-11-024 and #04-03-009. Athymic nude mice (Hsd:Athymic Nude-Foxn1nu, female, 20-25 g, 6 weeks old; RRID:MGI:5652489) and B6D2F1 mice (male, 4-5 wks, Jackson Laboratory; RRID:IMSR_JAX:100006) were allowed to

acclimatize at the MSKCC vivarium for 1 week prior to experiments. Mice were housed in ventilated cage enclosures in an environment maintained at 50% humidity with ambient temperatures range between 66 °F and 78 °F and 12h day/light cycles. Mice were provided with food and water ad libitum. All mice in all studies were observed for clinical signs at least once daily.

Wild animals: The study did not involve wild animals

Field-collected samples: none

Ethics oversight: All procedures were approved by the MSKCC Institutional Animal Care and Use Committee.

Note that full information on the approval of the study protocol must also be provided in the manuscript.

Human research participants

Policy information about studies involving human research participants

Population characteristics: Patients with solid malignancy, myeloproliferative neoplasm, myeloma, and/or lymphoma (histology confirmed by MSKCC Department of Pathology). Disease is measurable or evaluable as defined by RECIST (1.1 or original version) or other tumor response criteria from an MSKCC IRB-approved clinical research protocol.
Patients have established diagnosis of mild-moderate Alzheimer's Disease by board-certified neurologist (MSKCC or non-MSKCC) based upon neurological and neuropsychological evaluation following the National Institute on Aging- Alzheimer's Disease Association criteria that recently revisited the NINCDS-ADRDA criteria
Ages Eligible for Study: 18 Years to 90 Years (Adult, Older Adult)
Sexes Eligible for Study: All
Given the diverse population of patients at MSKCC, minorities and women patients had full access to this study and were fairly represented in the accrual.

Recruitment: Patients were screened by trained personnel, and the principal investigator was responsible for evaluating and confirming eligibility. Informed consent and HIPAA compliance forms were obtained from all subjects prior to their enrollment. Any patient who met the inclusion/exclusion criteria as detailed in the study protocol was considered for enrollment, therefore, to the best of our knowledge, there was no selection bias during recruitment.

Ethics oversight: The microdose 124I-PU-AD PET-CT (Dunphy, M. PET Imaging of Subjects Using 124I-PU-AD available from: http://clinicaltrials.gov; NCT03371420) and 124I-PU-H71 PET-CT (Dunphy, M. PET Imaging of Cancer Patients Using 124I-PUH71: A Pilot Study available from: http://clinicaltrials. gov; NCT01269593) were approved by the institutional review board, and conducted under an exploratory investigational new drug (IND) application approved by the US Food and Drug Administration.

Note that full information on the approval of the study protocol must also be provided in the manuscript.

Clinical data

Policy information about clinical studies

All manuscripts should comply with the ICMJEguidelines for publication of clinical research and a completedCONSORT checklist must be included with all submissions.

Clinical trial registration: NCT03371420 and NCT01269593

Study protocol: https://clinicaltrials.gov/

Data collection: NCT03371420: Five patients were enrolled and data collected at Memorial Sloan Kettering Cancer Center between 9/21/2016 and 7/21/2017, at which time the IND and trial sponsorship changed to Samus Therapeutics. NCT01269593: as reported in Dunphy et al. Clin Cancer Res; 2020 Oct 1;26(19):5178-5187. doi: 10.1158/1078-0432.CCR-19-3704. 124I-PU-HZ151 and 124I-PU-H71 were synthesized in-house by the institutional cyclotron core facility at high specific activity. Analyses of the epichaperome by positron emission tomography (PET-CT) were performed as previously reported. In brief, research PET-CT was performed using an integrated PET-CT scanner (Discovery DSTE, GE Healthcare). CT scans for attenuation correction and anatomic coregistration were performed before tracer injection. Patients received 185 megabecquerel (MBq) of 124I-PU-HZ151 or 124I-PU-H71 by peripheral vein over two minutes. PET data were reconstructed using a standard ordered subset expected maximization iterative algorithm. Emission data were corrected for scatter, attenuation, and decay. PET scans were performed 0.5, 3 and 24 h after tracer administration. For blood PK, all patient blood samples had a valid chain of custody from venipuncture through onsite specimen analysis and disposal. For [124I]-PU-HZ151 injection and blood sampling, separate peripheral intravenous lines were placed. Blood samples were collected 1, 5, 15, 30, 60-90 and 150-270 min post injection.

Outcomes	Primary Outcome Measures :<br>Pharmacokinetic profile of 124I-PU-AD: area under the curve (AUC) [Time Frame: 1 week]<br>Pharmacokinetic profile of 124I-PU-AD: maximum plasma concentration (Cmax) [Time Frame: 1 week]<br>Pharmacokinetic profile of 124I-PU-AD: trough plasma concentration (Cmin) [Time Frame: 1 week]<br>Pharmacokinetic profile of 124I-PU-AD: plasma half-life (T1/2) [Time Frame: 1 week]<br>Pharmacokinetic profile of 124I-PU-AD: time to maximum plasma concentration (Tmax) [Time Frame: 1 week]<br><br>Secondary Outcome Measures :<br>Incidence of adverse events [Time Frame: 30 days]<br>Safety of 124I-PU-AD in subjects as assessed by evaluation of the incidence, nature, and severity of adverse events and serious adverse events.

Flow Cytometry

Plots

Confirm that:

- [x] The axis labels state the marker and fluorochrome used (e.g. CD4-FITC).
- [x] The axis scales are clearly visible. Include numbers along axes only for bottom left plot of group (a 'group' is an analysis of identical markers).
- [x] All plots are contour plots with outliers or pseudocolor plots.
- [x] A numerical value for number of cells or percentage (with statistics) is provided.

Methodology

Sample preparation	Single cell suspension was prepared from a freshly resected GBM tumor as described in Online methods and CD105+ tumor endothelial cells (ECs) were isolated by a MoFlo cell sorter (Beckman Coulter, Pasadena, CA) using a FITC-conjugated CD105 antibody (1:20, BD Biosciences).
Instrument	MoFlo cell sorter (Beckman Coulter, Pasadena, CA)
Software	FowJo version 10
Cell population abundance	An aliquot of the CD105+ post-sort fraction was analyzed by a fluorescence microscope. The purity of CD105+ cells was 98%.
Gating strategy	FSC/SSC gates were set to exclude debris in the sample. CD105-FITC signal (FL1: green channel) was plotted against cell autofluorescence (FL2: orange channel) to allow a clear separation of CD105+ vs CD105- cells on a two-dimensional plot.

- [x] Tick this box to confirm that a figure exemplifying the gating strategy is provided in the Supplementary Information.

Magnetic resonance imaging

Experimental design

Design type	Standard brain MRI obtained as part of routine patient care. MRI brain were performed per institutional standard of care according to the standardized brain tumor imaging protocol (https://pubmed.ncbi.nlm.nih.gov/26250565/)
Design specifications	Experimental design specifications are not applicable as the brain MRI was obtained as standard-of-care (ie, routine brain MRI is a common, non-experimental procedure in the hospital setting). Standard brain MRI can vary in technical details; the technical details of the MRI procedure (ie, image acquisition protocol) employed for the MRI scans used in this research project are therefore provided below (under Acquisition, Preprocessing, Statistical Modeling).
Behavioral performance measures	N/A. The brain MRI was performed for tumor imaging as part of cancer patient care. Any quantitative analysis to correlate behavioral performance was not part of the study scale. Therefore, behavioral performance was not measured.

Acquisition

Imaging type(s)	Structural, diffusion, perfusion
Field strength	1.5T
Sequence & imaging parameters	Images were acquired using 5-mm slice thickness with no interslice gap (repetition time (TR) / echo time (TE) = 500/10 msec, matrix 256×256). Gadopentetate dimeglumine (Magnevist, HealthCare Pharmaceuticals Inc.) was injected though a peripheral angiocatheter at a standard dose (0.2 mL/kg body weight, 40 mL, 2 cc/sec).
Area of acquisition	whole brain

Diffusion MRI ☒ Used ☐ Not used

Parameters: Single shell DWI. 3 gradient directions applied. b=0,1000 s/mm2. Cardiac gating was not applied.

Preprocessing

Preprocessing software	For precise localization of tracer uptake to specific neuroanatomical structures, PET brain images were fused to MRI brain images using the Integrated Registration application of the AW Suite software package (GE Healthcare, Milwaukee, Wisconsin, USA) and using the standard T1 and T2 weighted sequences, for which no pre-processing is required. ADC maps were generated, as part of routine MRI protocol, although (for clarity) these were not used in the PET/MRI display and analysis. Again, MRI images (T1 and T2 sequences specifically) were used only for anatomic localization of radiotracer uptake (from fused PET image data). For sake of answering question, ACD maps were generated using ReadyView (GE software) that applies background noise and artifact removal.
Normalization	MRI brain were performed per institutional standard of care according to the standardized brain tumor imaging protocol (https://pubmed.ncbi.nlm.nih.gov/26250565/). For precise localization of tracer uptake to specific neuroanatomical structures, PET brain images were fused to MRI brain images using the Integrated Registration application of the AW Suite software package (GE Healthcare, Milwaukee, Wisconsin, USA). ADC maps were generated, as part of routine MRI protocol, although these were not used in the PET/MRI analysis. For sake of answering question, ACD maps were generated using ReadyView (GE software). No normalization is involved between subjects.
Normalization template	MRI brain were performed per institutional standard of care according to the standardized brain tumor imaging protocol (https://pubmed.ncbi.nlm.nih.gov/26250565/). For precise localization of tracer uptake to specific neuroanatomical structures, PET brain images were fused to MRI brain images using the Integrated Registration application of the AW Suite software package (GE Healthcare, Milwaukee, Wisconsin, USA). ADC maps were generated, as part of routine MRI protocol, although these were not used in the PET/MRI analysis. For sake of answering question, ACD maps were generated using ReadyView (GE software). No normalization was involved between subjects, as no group analysis attempting to extract a common result from the study group was performed.
Noise and artifact removal	MRI brain were performed per institutional standard of care according to the standardized brain tumor imaging protocol (https://pubmed.ncbi.nlm.nih.gov/26250565/). For precise localization of tracer uptake to specific neuroanatomical structures, PET brain images were fused to MRI brain images using the Integrated Registration application of the AW Suite software package (GE Healthcare, Milwaukee, Wisconsin, USA). ADC maps were generated, as part of routine MRI protocol, although these were not used in the PET/MRI analysis. For sake of answering question, ACD maps were generated using ReadyView (GE software) that applies background noise and artifact removal.
Volume censoring	MRI brain were performed per institutional standard of care according to the standardized brain tumor imaging protocol (https://pubmed.ncbi.nlm.nih.gov/26250565/). Volume censoring is not applicable when the DWI is relatively short scan and it does not require multiphase volumetric acquisition.

Statistical modeling & inference

Model type and settings	Statistical modeling & inference is not part of standard MRI software data processing, nor was any statistical analysis performed, comparing MRI data between patients. The MRI scans were used solely for the purpose of precise localization of radiotracer uptake to specific neuroanatomical structures, on software-based MRI/PET fusion image display.
Effect(s) tested	Not applicable because there was no statistical analysis performed, comparing MRI data between patients. The MRI scans were used solely for the purpose of precise localization of radiotracer uptake to specific neuroanatomical structures, on software-based MRI/PET fusion image display.

Specify type of analysis: ☐ Whole brain ☐ ROI-based ☐ Both

Statistic type for inference (See Eklund et al. 2016)	Not applicable because there was no statistical analysis performed, comparing MRI data between patients. The MRI scans were used solely for the purpose of precise localization of radiotracer uptake to specific neuroanatomical structures, on software-based MRI/PET fusion image display.
Correction	Not applicable because there was no statistical analysis performed, comparing MRI data between patients. The MRI scans were used solely for the purpose of precise localization of radiotracer uptake to specific neuroanatomical structures, on software-based MRI/PET fusion image display.

Models & analysis

n/a	Involved in the study
☒	☐ Functional and/or effective connectivity
☒	☐ Graph analysis
☒	☐ Multivariate modeling or predictive analysis
